# Supplementary material for: Bubble-PAPR: a phase 1 clinical evaluation of the comfort and perception of a prototype powered air-purifying respirator for use by healthcare workers in an acute hospital setting
Source: BMJ Open. 2023 May 8;13(5):e066524. doi: 10.1136/bmjopen-2022-066524 (PMC10174029; doi:10.1136/bmjopen-2022-066524)
Supplement: Supplementary data [file bmjopen-2022-066524supp002.pdf]

Table S1. (Supplemental) Proposed framework for the safer adoption of a MacGyvered device. Adapted from Turner and colleagues.[7]

1. Define the problem and rule out the suitability of existing solutions
2. List benchmark safety indices for the device
3. Seek broader feedback from all stakeholders on the design's utility and potential pitfalls.
4. Perform laboratory-based and in situ simulations.
5. Introduce into low-risk clinical settings after local due process and patient consent.
6. Introduce into higher-risk clinical settings with a discrete group of trained 'super-users'.
7. Encourage an iterative cycle of feedback, review, re-design and improvement.
8. **Do not:** adopt, publish, endorse or disseminate via social media a MacGyvered device without data to support safety.

McGrath et al. Supplemental Tables

Table S2. (Supplemental). Lab-based testing of the Bubble PAPR prior to clinical evaluation

All of the bench tests detailed below were carried out by the Electrical and Biomedical Engineering (EMBE) team based at Wythenshawe Hospital (MFT), the University of Manchester Mechanical Aerospace and Civil Engineering team (UoM) or by INSPEC International, Salford, UK. A PAPR unit was supplied, and the Instructions for Use were followed by the independent tester, with a judgement made if they fulfilled particular requirements. Some requirements are supplemented by the qualitative or quantitative data collected in the questionnaires. Standards used were British Standard EN12941 (BS, 2008) and the European Regulations for Respiratory Protective Equipment EU2016/425 (ER, 2016).

Relevant section of standard	Standard detail	Test location	Test detail	Results/notes	Pass/Fail
BS 6.1.1<br>ER 1.3.2	Suitable resistance to wear and tear	MFT	PAPR units inspected after 1 week of continual use.<br>Images taken before and after.	Opinion. Baseline inspection +/- photograph. Review after 1 week	Pass 14/3/21
BS 6.1.4<br>ER 1.2.1.2	No sharp edges	MFT	Visual and physical inspection<br>Reports from staff evaluation	Opinion. Baseline inspection +/- photograph. Review after 1 week	Pass 14/3/21
BS 6.3.2	Fits a range of head sizes	MFT	Ten participants will undergo fit testing.<br>These participants will have height, weight and head circumference measured as part of this standard process.	Fit test data shared with EBME team. All fit factors >500 as per BS EN 12941 standard.	Pass 25/2/21
BS 6.3.3.1	Does not distort vision	MFT	The optical area appears transparent.	Inspection by EBME team	Pass 25/2/21
BS 6.3.3.2	Permits appropriate field of view	MFT	Reports from staff evaluation	Review of results from initial staff evaluations	Pass 14/3/21

McGrath et al. Supplemental Tables

ER2.3					
Relevant section of standard	Standard detail	Test location	Test detail	Results/notes	Pass/Fail
BS 6.9	Cannot reverse airflow	MFT	Normal use.<br>Simulate blocked filter and blocked air duct.<br>Flowmeter.		Pass 14/3/21
BS 6.9	Battery safe – protection from short circuit	MFT	EBME check on battery packs (suitable for purpose / recommended packs)	As per manufacturer documentation. Will not be separately tested.	Pass 25/2/21
ER2.12	Appropriate markings	MFT	Yoke manufactured with section for appropriate sticker		Pass 25/2/21
ER3.10.1	Appropriate training provided	MFT	Instructions for use provided.<br>Training videos provided.	Instructions for Use provided to EBME.<br>Training videos available.	Pass 25/2/21
No specific clause	Cleanable	MFT	Specification of foam material for yoke details cleaning methods, durability and material fatigue.	Specification provided to EBME.	Pass 25/2/21
BS 6.16	Mass shall not exceed 5kg. A maximum of 1.5kg shall be carried on the head.	MFT	Weigh the assembled Bubble PAPR	Assembled Bubble PAPR Weight = 1.4kg	Pass 14/3/21

McGrath et al. Supplemental Tables

Relevant section of standard	Standard detail	Test location	Test detail	Results/notes	Pass/Fail
BS 6.1.3	Repeated cleaning and disinfection – does not deteriorate	MFT	PAPR units are inspected after 1 week of continual use. Images taken before and after.<br><br>Specification of foam material for yoke details cleaning methods, durability and material fatigue.	Spec sheet and review after 1 week of use.	Pass 14/3/21
BS 6.4	Ingress protection test for 10 test subjects – using either or both methods (bitter and/or particulometer).	MFT	10 users will undergo fit testing with particulometer.	Fit test data shared with EBME team. All fit factors >500 as per BS EN 12941 standard.	Pass 25/2/21
ER 3.10.2	Appropriate protection to eye and skin irritants	MFT			

McGrath et al. Supplemental Tables

Relevant section of standard	Standard detail	Test location	Test detail	Results/notes	Pass/Fail
BS 6.2 (6.4)	Repeated after hood/yoke is conditioned at maximum specified temperature and humidity.<br><br>Complete unit is stored for 72 +/- 1 hours at the upper extreme of temperature and humidity specified by the manufacturer. Unit is allowed to return to ambient conditions for 4 hours, then stored for 72 +/- 1 hours at the lower extreme of temperature and humidity.	MFT	Unit is subjected to particulometer fit testing after appropriate temperature conditioning.	*Conditioning beyond use on the ICU should not be required for the MFT in-house evaluation*<br><br>All fit tests took place on the Acute ICU at Wythenshawe	Pass 25/2/21
BS 6.5	Positive pressure inside the hood remains below 5mbar	MFT	1 user and 1 dummy test head setup. Pressure measurement inside hood during regular use.	Measured pressure below 5mBar	Pass 14/3/21
BS 6.6.2	Exceeds manufacturer's minimum specified airflow for a period of at least 4 hours.<br><br>(The UK/EU regs do not specify a minimal flow. US regulations do, but this is not immediately relevant)	MFT, INSPEC	Test head and flow meter.<br><br>*Note the flow meter arrangement is slightly complex as the measurement itself can interfere with flow.*	Breathe Safety report reviewed.	Pass 25/2/21

McGrath et al. Supplemental Tables

Relevant section of standard	Standard detail	Test location	Test detail	Results/notes	Pass/Fail
BS 6.7	Check function of minimum airflow indicator.	MFT, INSPEC	Apply different flow rates to the yoke measured by external flow meter and evaluate performance of the minimum airflow indicator in units.	Breathe Safety report reviewed.	Pass 25/2/21
EN 143<br>BS 6.8	Clogging of filter	MFT, INSPEC	Flow through filter and yoke tested after 4 hours of use with an external flow meter.	Breathe Safety report reviewed.	Pass 25/2/21
6.13	The carbon dioxide content of the inhalation air (dead space inside the hood) shall not exceed an average of 1% by volume.<br><br>There is a specific test-rig setup for this. A physiological surrogate model should provide adequate assurance that the CO_2 content inside the hood is <1% during normal use.	MFT, INSPEC	Oxygen and carbon dioxide (gas analysis) inside the hood measured as partial pressures and/or percentages using MFT EBME equipment.	Breathe Safety report reviewed.	Pass 25/2/21

McGrath et al. Supplemental Tables

Relevant section of standard	Standard detail	Test location	Test detail	Results/notes	Pass/Fail
BS 6.15	Exhalation means (valve) maximum flowrate and safe operation:<br><br>Hood performs adequately during normal use. Specifically; exhalation means:<br>• functions and can be replaced (new hood)<br>• functions in orientations encountered during normal use<br>• is protected against dirt and mechanical damage	MFT	Test subject wears hood.<br><br>Eventually, this section is supplemented with reports from staff evaluation.	Review of initial feedback from users in sim setting	Pass 14/3/21
BS 6.15	Exhalation means (valve) maximum flowrate and safe operation:<br><br>Continuous flow rate of 300 +/- 15 L/min is applied for a period of 60+/- 6 secs.	MFT	Flow generator (ventilator) and flow meter.<br><br>Visual inspection of exhalation valve.	Opinion of EBME team.<br>The flow generator works as described with the test flowmeter supplied.<br>Exhalation Valve inspection pass.	Pass 14/3/21

McGrath et al. Supplemental Tables

Table S3. (Supplemental). Pilot data. Q14 & Q17 are Likert Scale items (rated 1-7) and Q8-11 are Visual Analogue Scale items (rated 0-100).

	Current PPE						BUBBLE-PAPR					
	Q14 Safe to wear	Q17 Comfort to wear	Q8 Speak clearly to colleagues	Q9 Be heard by colleagues	Q10 Speak clearly to patients	Q11 Be heard by patients	Q14 Safe to wear	Q17 Comfort to wear	Q8 Speak clearly to colleagues	Q9 Be heard by colleagues	Q10 Speak clearly to patients	Q11 Be heard by patients
Participant												
1	3	3	10	20	0	0	4	5	75	75	80	80
2	4	2	15	20	10	30	7	5	85	80	60	70
3	4	4	20	20	30	30	5	6	90	90	90	90
4	5	2	25	25	10	10	6	6	95	95	95	85
5	5	4	33	40	25	25	6	5	75	70	85	90
6	6	3	30	25	30	30	6	5	65	70	55	66
7	7	2	20	30	25	25	7	5	70	70	65	65
8	4	3	45	50	20	30	7	6	90	95	90	90
Mean	4.8	2.9	24.8	28.8	18.8	22.5	6.0	5.4	80.6	80.6	77.5	79.5
SD	1.3	0.8	11.1	10.9	10.9	11.3	1.1	0.5	10.8	11.2	15.4	11.0

McGrath et al. Supplemental Tables

Table S4. (Supplemental) Fit testing data from the first 10 participants.

Test protocol HSE INDG 479. Pass level set at a fit factor of 500.

Subject	Self-reported height	Self-reported weight	BMI	Normal Breathing 1	Deep Breathing	Head Side to Side	Head Up and Down	Talking	Bending at the waist	Normal Breathing 2	Overall Fit Factor
**1**	1.86	74	21.4	79705	43647	125478	11125	107899	1339	76152	**7757**
**2**	1.95	75	19.7	53792	52343	59440	51673	52733	50433	45961	**52075**
**3**	NR	NR	NR	38867	36699	37097	41474	39500	36884	37465	**38217**
**4**	1.82	65	19.6	17745	6622	3149	5028	31996	30520	31326	**8539**
**5**	1.65	55	20.2	24945	25215	3885	8097	28877	29107	24393	**12268**
**6**	1.67	58	20.8	24617	25608	25581	25225	20107	1924	23517	**9088**
**7**	1.52	47	20.3	28747	30700	33203	15275	31671	8327	26041	**19829**
**8**	1.65	65	23.9	27282	31318	34900	9697	29093	3514	25770	**12544**
**9**	1.83	115	34.3	11182	1123	11028	24524	24692	2537	23704	**4408**
**10**	1.59	75	29.7	25760	3419	16125	2433	25523	1552	26154	**4588**
**Mean values**				**33264**	**25669**	**34989**	**19455**	**39209**	**16614**	**34048**	**16931**

McGrath et al. Supplemental Tables
