# Supplementary material for: Bubble-PAPR: a phase 1 clinical evaluation of the comfort and perception of a prototype powered air-purifying respirator for use by healthcare workers in an acute hospital setting
Source: BMJ Open. 2023 May 8;13(5):e066524. doi: 10.1136/bmjopen-2022-066524 (PMC10174029; doi:10.1136/bmjopen-2022-066524)
Supplement: Supplementary data [file bmjopen-2022-066524supp003.pdf]

Table S5. Rating scales, summary results and comparisons across all of the questionnaire domains.

	PPE	Q1 Confidence in donning	Q2 Confidence in donning without dislodging other PPE	Q3 Wear with glasses/goggles	Q4 Protect yourself from respiratory infection	Q5 Protect patient from infection from you	Q6 Safely care for your patient	Q7 Safely roll patient	Q8 Speak to staff	Q9 Be heard by staff	Q10 Speak to patient	Q11 Be heard by patient	Q12 Doff safely	Q13 Doff without dislodging glasses
Rating scale	From:	0 - no confidence	0 - no confidence	0 - no confidence	0 - no confidence	0 - no confidence	0 - no confidence	0 - no confidence	0 - no confidence	0 - no confidence	0 - no confidence	0 - no confidence	0 - no confidence	0 - no confidence
	To:	10 - fully confident	10 - fully confident	10 - fully confident	10 - fully confident	10 - fully confident	10 - fully confident	10 - fully confident	10 - fully confident	10 - fully confident	10 - fully confident	10 - fully confident	10 - fully confident	10 - fully confident
RPE type	FFP3	8.9 (1.4) [3 - 10]	8.3 (2) [2 - 10]	6.9 (2.6) [2 - 10]	8.2 (1.6) [4 - 10]	8.2 (1.7) [2 - 10]	8.4 (1.4) [5 - 10]	8.2 (1.8) [2 - 10]	5.1 (2.4) [1 - 10]	4.9 (2.3) [1 - 10]	4.8 (2.4) [1 - 10]	4.7 (2.5) [1 - 10]	8.1 (1.9) [2 - 10]	6.2 (2.5) [1 - 10]
	Bubble	7.4 (1.8) [3 - 10]	7.7 (1.8) [2 - 10]	7.6 (1.9) [3 - 10]	8.6 (1.6) [3 - 10]	8.5 (1.8) [2 - 10]	8.0 (2) [2 - 10]	7.8 (2.2) [2 - 10]	7.5 (2.4) [1 - 10]	7.1 (2.3) [1 - 10]	7.8 (2.1) [2 - 10]	7.4 (2.4) [1 - 10]	8.0 (1.8) [2 - 10]	7.8 (1.9) [2 - 10]
Comparison	Mean difference	-1.48	-0.55	0.7	0.43	0.3	-0.42	-0.42	2.38	2.16	2.99	2.7	-0.1	1.66
	95% CI	-1.9 to -0.99	-1.12 to 0.02	-0.00 to 1.40	-0.04 to 0.89	-0.18 to 0.78	-0.91 to 0.07	-0.98 to 0.15	1.66 to 3.11	1.45 to 2.88	2.36 to 3.62	1.97 to 3.43	-0.63 to 0.43	0.98 to 2.34
		Favours FFP3	No difference	Favours Bubble	No difference	No difference	No difference	No difference	Favours Bubble	Favours Bubble	Favours Bubble	Favours Bubble	No difference	Favours Bubble
	Adjusted p	<0.001	0.058	0.049	0.070	0.217	0.092	0.144	<0.001	<0.001	<0.001	<0.001	0.711	<0.001

	PPE	Q14 How safe does it feel	Q15 Worried about own health	Q16 Worried others health	Q17 Comfortable	Q18 Don ease	Q19 Doff ease	Q20a Restricted communication	Q20b Vision distorted with Bubble	Q20c Read monitors, computers, and notes with Bubble	Q21 Pressure marks on head/face	Q22 Pain on head/face	Q23 Leave clinical area early due to RPE
Rating scale	From:	1 - very unsafe	1 - not worried at all	1 - not worried at all	1 - very uncomfortable	1 - not at all easy	1 - not at all easy	1 - not at all restricted	1 - not at all affected	1 - clear at all times	1 - never	1 - never	1 - never
	To:	7 very safe	7 - very worried	7 - very worried	7 - very comfortable	7 - very easy	7 - very easy	7 - very restricted	7 - very affected	7 - not clear at all	7 - always	7 - always	7 - always
RPE type	FFP3	5.4 (1.0) [3 - 7]	3.2 (1.5) [1 - 7]	3.2 (1.5) [1 - 7]	3 (1.4) [1 - 6]	4.9 (1.3) [2 - 7]	5.1 (1.3) [2 - 7]	5.4 (1.4) [1 - 7]	-	-	5.8 (1.4) [1 - 7]	5.3 (1.4) [1 - 7]	3.6 (1.6) [1 - 7]
	Bubble	6.2 (0.9) [3 - 7]	2.3 (1.6) [1 - 7]	2.3 (1.7) [1 - 7]	5.6 (1.6) [1 - 7]	5.5 (1.4) [2 - 7]	5.7 (1.2) [2 - 7]	3.9 (1.7) [1 - 7]	3.2 (1.9) [1 - 7]	5.6 (1.5) [2 - 7]	1.3 (0.8) [1 - 6]	1.4 (0.9) [1 - 6]	1.5 (1) [1 - 6]
Comparison	Mean difference	0.73	-0.92	-0.93	2.68	0.62	0.63	-1.49	-	-	-4.54	-3.99	-2.13
	95% CI	0.45 to 0.99	-1.36 to -0.49	-1.36 to -0.48	2.23 to 3.14	0.21 to 1.02	0.26 to 0.99	-1.95 to -1.04	-	-	-4.90 to -4.17	-4.35 to -3.63	-2.51 to - 1.75
		Favours Bubble	Favours Bubble	Favours Bubble	Favours Bubble	Favours Bubble	Favours Bubble	Favours Bubble	No comparator	No comparator	Favours Bubble	Favours Bubble	Favours Bubble
	Adjusted p	<0.001	<0.001	<0.001	<0.001	0.003	0.002	<0.001	-	-	<0.001	<0.001	<0.001
