# Supplementary material for: Bubble-PAPR: a phase 1 clinical evaluation of the comfort and perception of a prototype powered air-purifying respirator for use by healthcare workers in an acute hospital setting
Source: BMJ Open. 2023 May 8;13(5):e066524. doi: 10.1136/bmjopen-2022-066524 (PMC10174029; doi:10.1136/bmjopen-2022-066524)
Supplement: Supplementary data [file bmjopen-2022-066524supp001.pdf]

BMJ Publishing Group Limited (BMJ) disclaims all liability and responsibility arising from any reliance placed on this supplemental material which has been supplied by the author(s)

Figure S1. (Supplemental) Participant job roles.

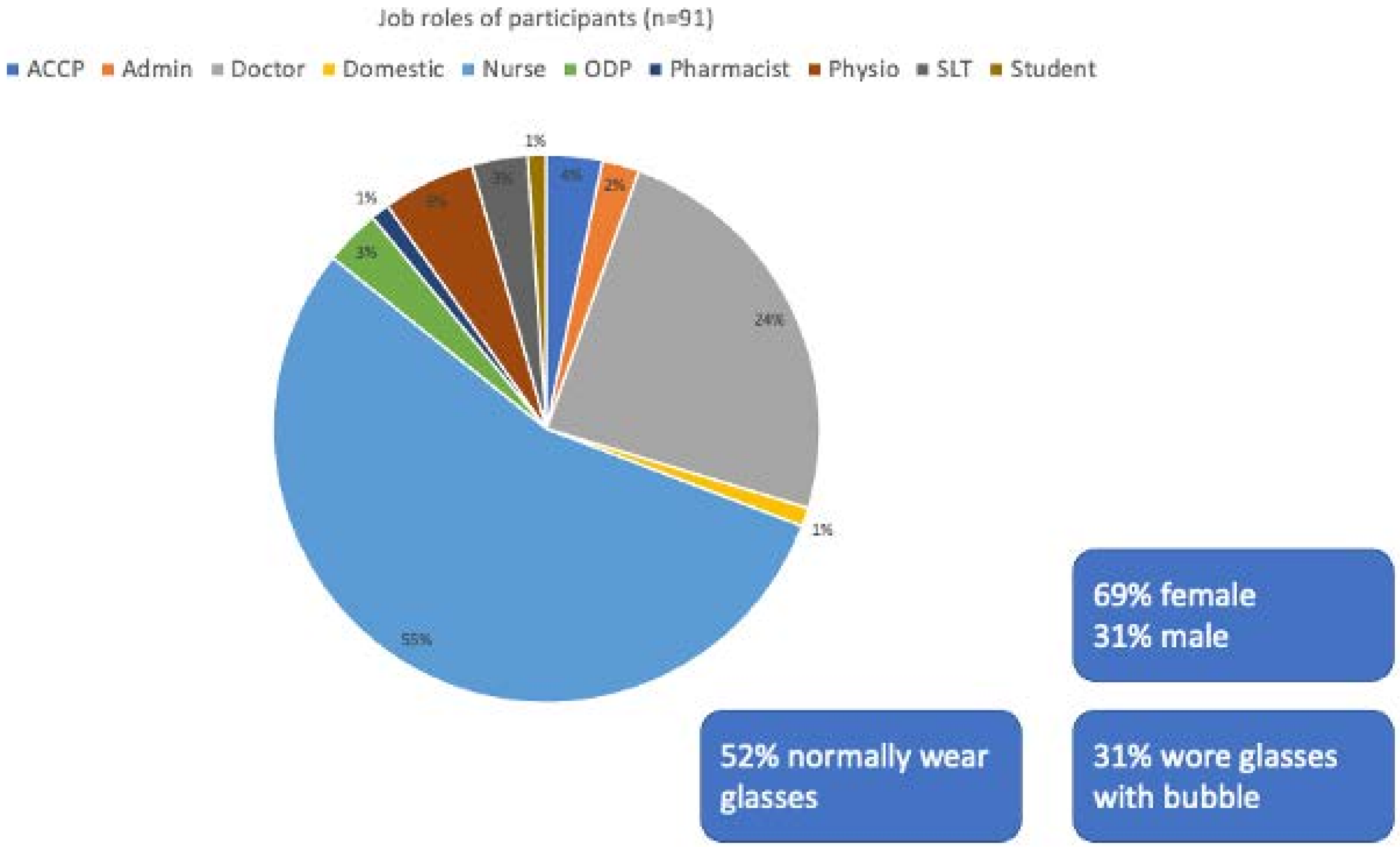

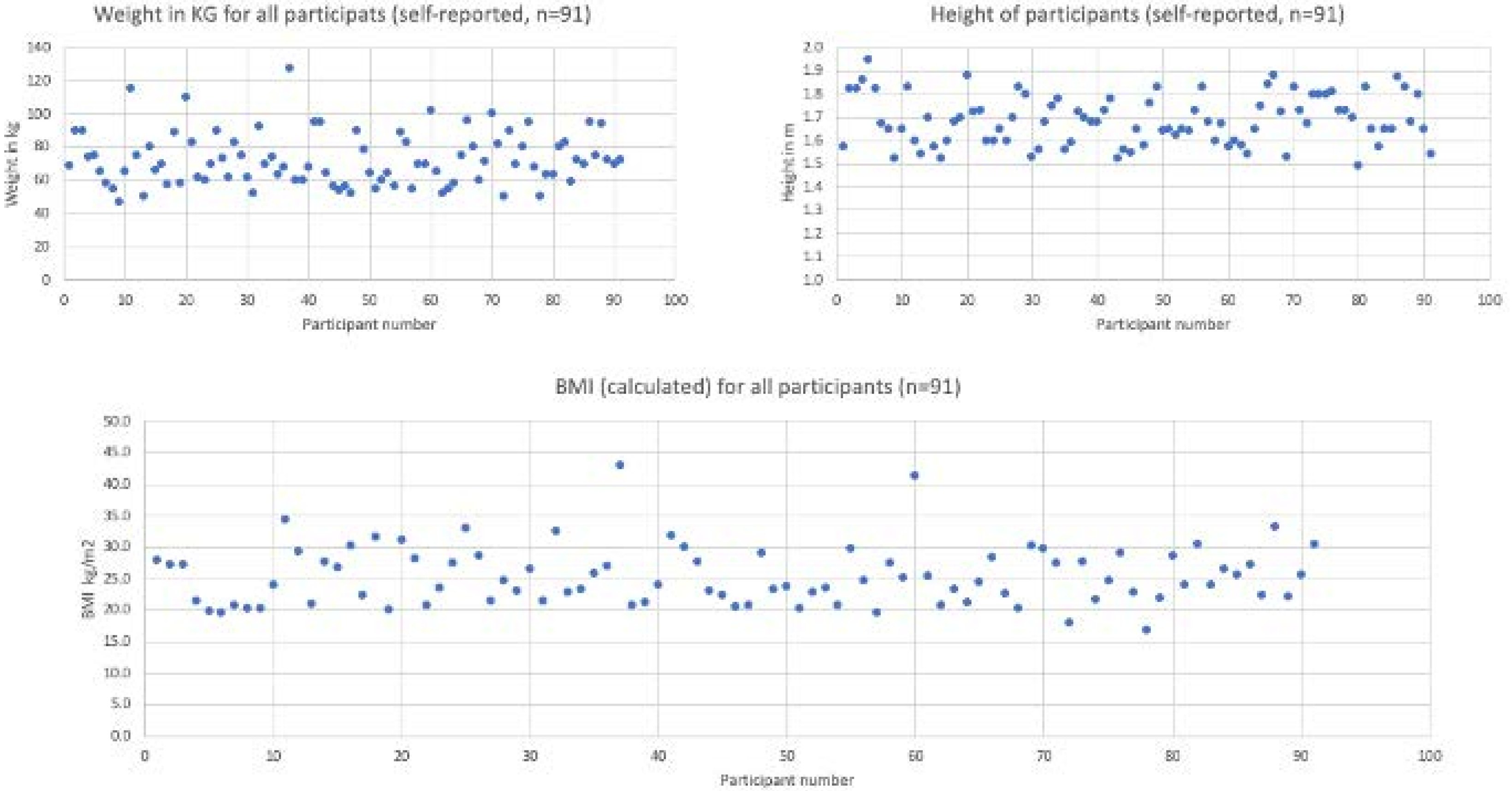

Figure S2. (Supplemental) Self-reported weight, height and BMI of staff participants.

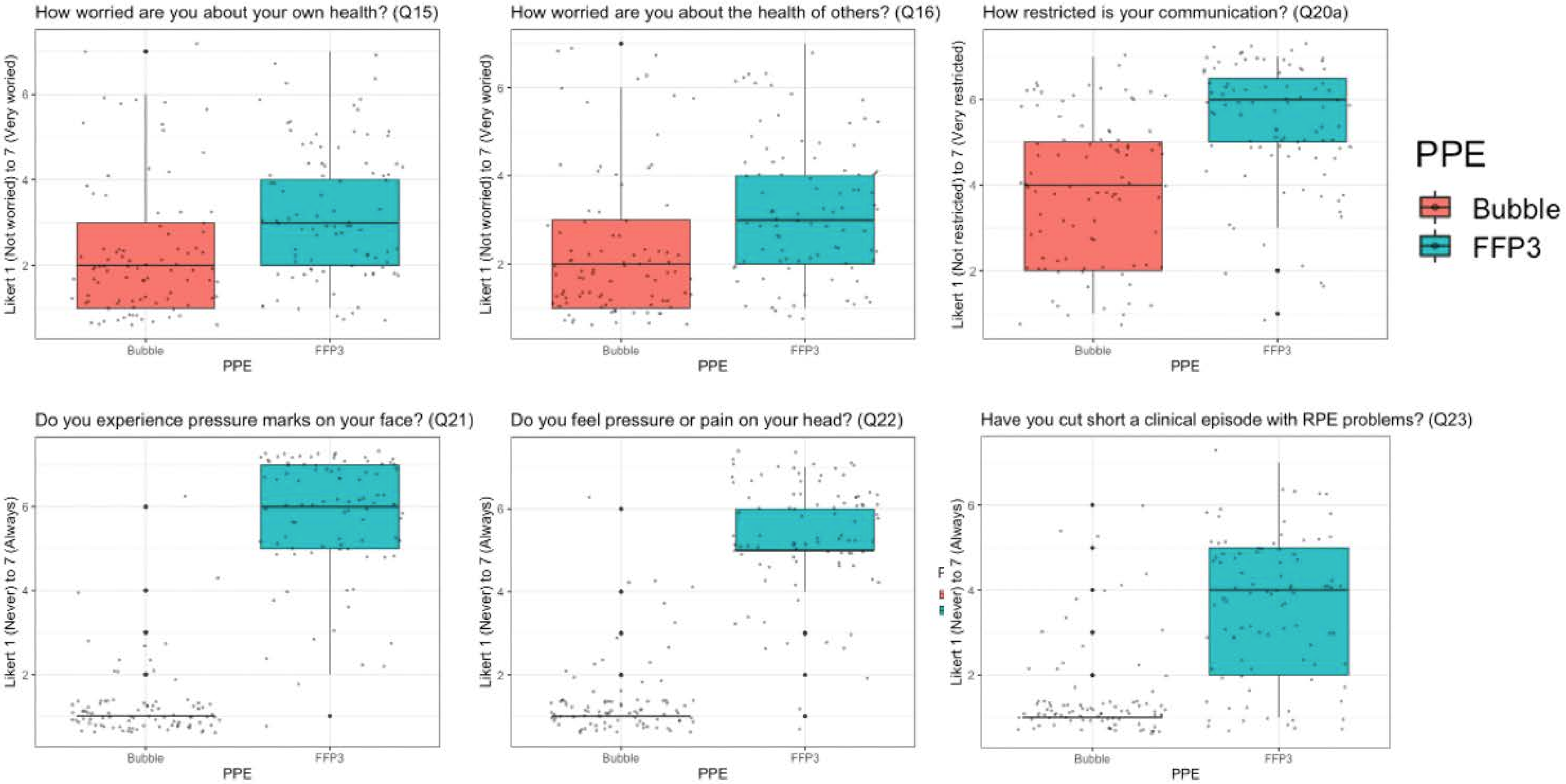

Figure S3. (Supplemental) Secondary outcomes where a lower Likert scale response was considered better.

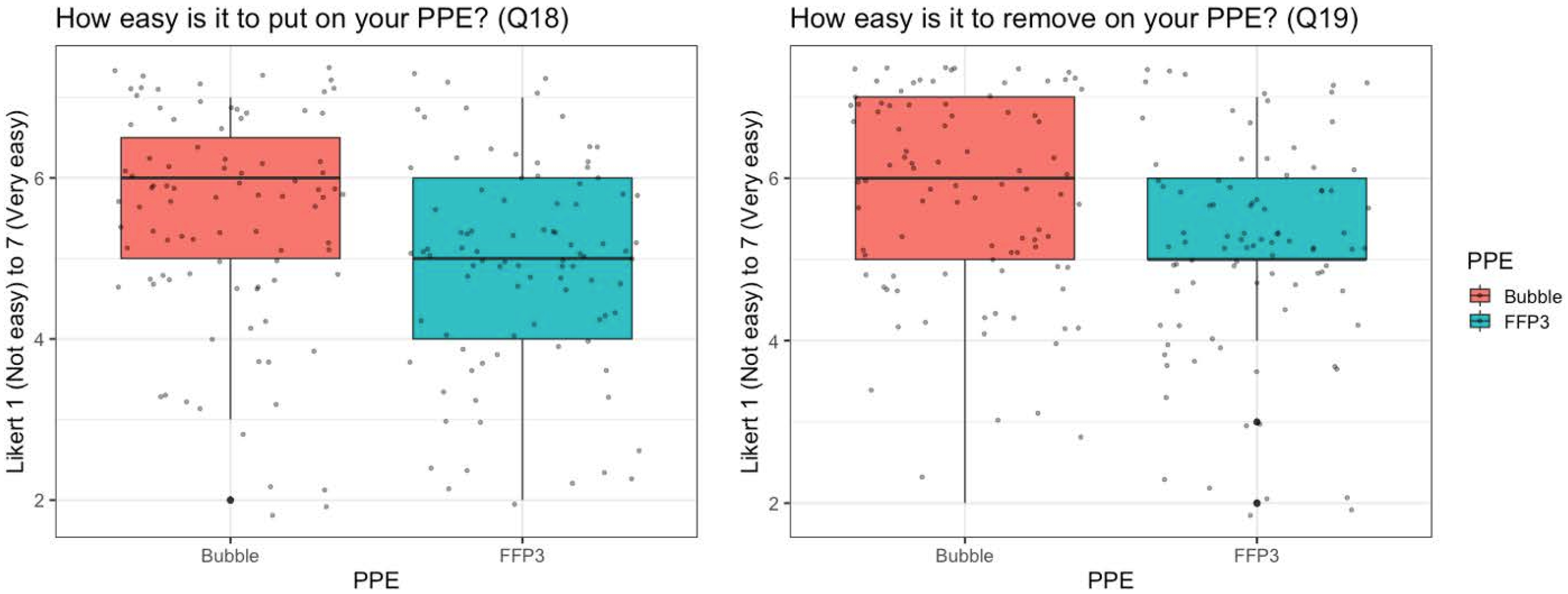

Figure S4. (Supplemental) Ease of donning and doffing of Respiratory Protective Equipment.

BMJ Publishing Group Limited (BMJ) disclaims all liability and responsibility arising from any reliance placed on this supplemental material which has been supplied by the author(s)

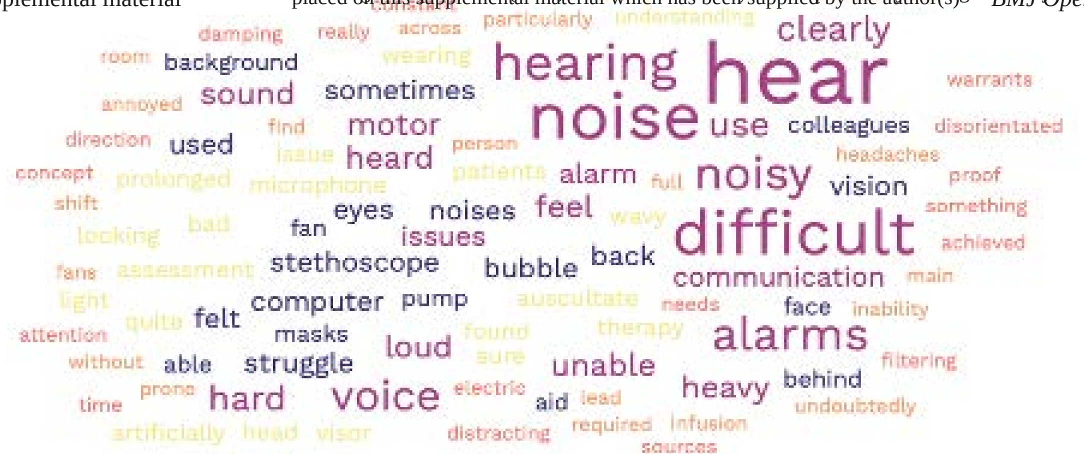

Figure S5. (Supplemental). Word clouds from the free text feedback. Negative comments (all categories).

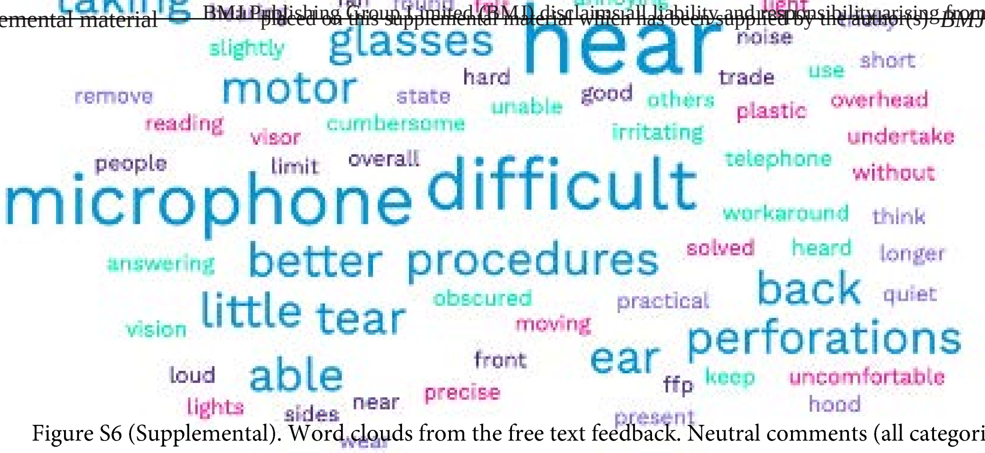

Figure S6 (Supplemental). Word clouds from the free text feedback. Neutral comments (all categories).

BMJ Publishing Group Limited (BMJ) disclaims all liability and responsibility arising from any reliance placed on this supplemental material which has been supplied by the author(s)

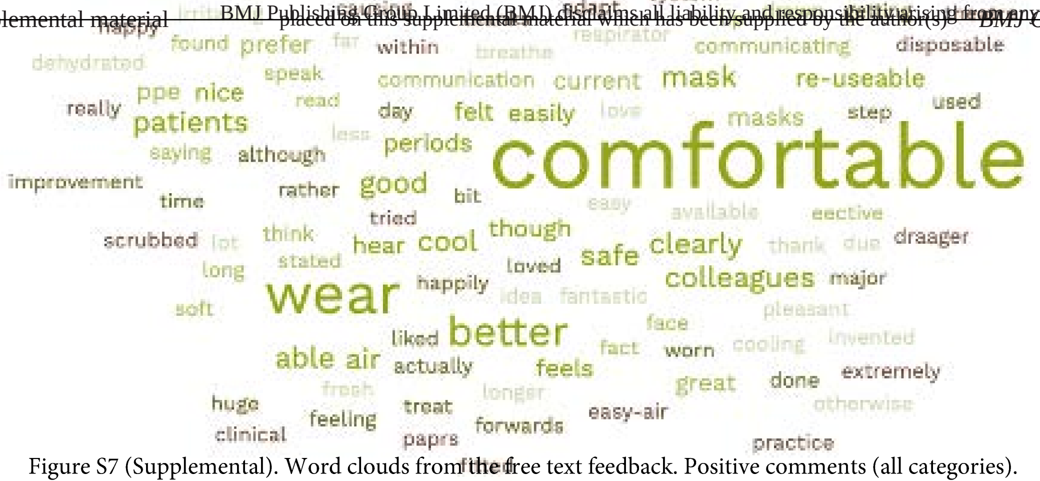

Figure S7 (Supplemental). Word clouds from the free text feedback. Positive comments (all categories).
