# Supplementary material for: Bubble-PAPR: a phase 1 clinical evaluation of the comfort and perception of a prototype powered air-purifying respirator for use by healthcare workers in an acute hospital setting
Source: BMJ Open. 2023 May 8;13(5):e066524. doi: 10.1136/bmjopen-2022-066524 (PMC10174029; doi:10.1136/bmjopen-2022-066524)
Supplement: Author's manuscript [file bmjopen-2022-066524.draft_revisions.pdf]

BMJ Open

BMJ Open is committed to open peer review. As part of this commitment we make the peer review history of every article we publish publicly available.

When an article is published we post the peer reviewers' comments and the authors' responses online. We also post the versions of the paper that were used during peer review. These are the versions that the peer review comments apply to.

The versions of the paper that follow are the versions that were submitted during the peer review process. They are not the versions of record or the final published versions. They should not be cited or distributed as the published version of this manuscript.

BMJ Open is an open access journal and the full, final, typeset and author-corrected version of record of the manuscript is available on our site with no access controls, subscription charges or pay-per-view fees (http://bmjopen.bmj.com).

If you have any questions on BMJ Open's open peer review process please email info.bmjopen@bmj.com

BMJ Open

# Bubble-PAPR: Phase I clinical evaluation of an 'in-house' developed prototype powered air-purifying respirator for use by healthcare workers

| | |
|---|---|
| Journal: | *BMJ Open* |
| Manuscript ID | bmjopen-2022-066524 |
| Article Type: | Original research |
| Date Submitted by the Author: | 12-Jul-2022 |
| Complete List of Authors: | McGrath, Brendan; Manchester University NHS Foundation Trust; Manchester Academic Health Science Centre, Immunity and Respiratory Medicine<br>Shelton, Clifford; NHS Manchester; Lancaster University Lancaster Medical School<br>Gardner, Angela; Manchester University NHS Foundation Trust<br>Coleman, Ruth; Manchester University NHS Foundation Trust<br>Lynch, James; Manchester University NHS Foundation Trust<br>Alexander, Peter G; Manchester University NHS Foundation Trust; Manchester Academic Health Science Centre, Immunity and Respiratory Medicine<br>Cooper, Glen; The University of Manchester School of Mechanical Aerospace and Civil Engineering |
| Keywords: | OCCUPATIONAL & INDUSTRIAL MEDICINE, INTENSIVE & CRITICAL CARE, Infection control < INFECTIOUS DISEASES, ANAESTHETICS, RESPIRATORY MEDICINE (see Thoracic Medicine) |
| | |

SCHOLARONE™
Manuscripts

*I, the Submitting Author has the right to grant and does grant on behalf of all authors of the Work (as defined in the below author licence), an exclusive licence and/or a non-exclusive licence for contributions from authors who are: i) UK Crown employees; ii) where BMJ has agreed a CC-BY licence shall apply, and/or iii) in accordance with the terms applicable for US Federal Government officers or employees acting as part of their official duties; on a worldwide, perpetual, irrevocable, royalty-free basis to BMJ Publishing Group Ltd ("BMJ") its licensees and where the relevant Journal is co-owned by BMJ to the co-owners of the Journal, to publish the Work in this journal and any other BMJ products and to exploit all rights, as set out in our licence.*

*The Submitting Author accepts and understands that any supply made under these terms is made by BMJ to the Submitting Author unless you are acting as an employee on behalf of your employer or a postgraduate student of an affiliated institution which is paying any applicable article publishing charge ("APC") for Open Access articles. Where the Submitting Author wishes to make the Work available on an Open Access basis (and intends to pay the relevant APC), the terms of reuse of such Open Access shall be governed by a Creative Commons licence – details of these licences and which Creative Commons licence will apply to this Work are set out in our licence referred to above.*

*Other than as permitted in any relevant BMJ Author's Self Archiving Policies, I confirm this Work has not been accepted for publication elsewhere, is not being considered for publication elsewhere and does not duplicate material already published. I confirm all authors consent to publication of this Work and authorise the granting of this licence.*

For peer review only

**Title:**
Bubble-PAPR: Phase I clinical evaluation of an 'in-house' developed prototype powered air-purifying respirator for use by healthcare workers

**Short title:**
Bubble-PAPR: a phase 1 study

**Authors**
Brendan A McGrath, Consultant & Honorary Professor[1,2]
Cliff Shelton, Consultant Clinical Senior Lecturer[1,3]
Angela Gardner, Clinical Fellow[1]
Ruth Coleman, Advanced Critical Care Practitioner[1]
James Lynch, Advanced Critical Care Practitioner[1]
Peter G Alexander, Consultant & Honorary Senior Lecturer[1,2]
Glen Cooper, Reader

1. Acute Intensive Care Unit, Wythenshawe Hospital, Manchester University NHS Foundation Trust
2. Manchester Academic Critical Care, Division of Infection, Immunity and Respiratory Medicine, School of Biological Sciences, Faculty of Biology, Medicine and Health, the University of Manchester, Manchester Academic Health Science Centre.
3. Lancaster Medical School, Lancaster University
4. Department of Mechanical, Aerospace & Civil Engineering, the University of Manchester

**Corresponding author**
Brendan A McGrath (CI)
Acute Intensive Care Unit
Wythenshawe Hospital
Southmoor Road
Wythenshawe
Manchester. M23 9LT
0161 291 6420
Brendan.mcgrath@manchester.ac.uk

For peer review only

**Structured abstract (299 words)**

**Objectives**: We aimed to design and produce a low-cost, ergonomic, hood-integrated Powered Air-Purifying Respirator (Bubble-PAPR) for pandemic healthcare use, offering optimal and equitable protection to all staff. We hypothesised that participants would rate Bubble-PAPR more highly than current FFP3 face mask respiratory protective equipment (RPE).

**Design**: Rapid design and evaluation cycles occurred based on the identified user needs. We conducted diary card and focus group exercises to identify relevant tasks requiring RPE. Lab-based safety standards established against British Standard BS-EN-12941 and EU2016/425. Questionnaire-based usability data from participating frontline healthcare staff before (usual RPE) and after using Bubble-PAPR.

**Setting**: Overseen by a trial safety committee, evaluation progressed sequentially through laboratory, simulated, low-risk, then high-risk clinical environments of a single tertiary NHS hospital.

**Participants:** 15 staff completed diary cards and focus groups. 91 staff from a range of clinical and non-clinical roles completed the study, wearing Bubble-PAPR for a median of 45 minutes (IQR 30-80 [15-120]). Participants self-reported a range of heights (mean 1.7m [SD 0.1, range 1.5-2.0]), weights (72.4kg [16.0, 47-127]) and body mass indices (25.3 [4.7,16.7-42.9]).

**Outcome measures**: Primary: *"How comfortable do you feel in your PPE?"* (Likert scale bounded by 1 [very uncomfortable] to 7 [very comfortable]). Secondary outcomes: perceived safety, communication, anxiety, discomfort, and performance.

**Results**: Bubble-PAPR mean comfort score was 5.64(SD 1.55) versus usual FFP3 2.96(1.44) (mean difference 2.68 (95% CI 2.23-3.14, p<0.001). There was a significant difference in favour of Bubble-PAPR across all secondary outcomes.

**Conclusions**: Bubble-PAPR achieved its primary purpose of keeping staff safe from airborne particulate material whilst improving comfort and the user experience. The design and development of Bubble-PAPR were conducted using a careful evaluation strategy addressing key regulatory and safety steps, in contrast to many devices rapidly developed and deployed during the pandemic.

**Trial registration**: IRAS ID:288493, REC Ref:21/WA/0018. ClinicalTrials.gov (NCT04681365).

For peer review only

**Strengths and limitations of this study**

- We employed user-centred design, engineering optimisation and staged feasibility testing to develop a novel Powered Air-Purifying Respirator (Bubble-PAPR) for use specifically in frontline healthcare settings.
- Diverse, frontline healthcare staff compared Bubble-PAPR with usual FFP3 face masks.
- The design and development of Bubble-PAPR were conducted using a careful strategy addressing key regulatory and safety steps, in contrast to many devices rapidly developed and deployed during the pandemic.
- Bubble-PAPR is an excellent example of developing a cosmopolitan network that could become a key feature of future system resilience.

For peer review only

## Introduction

The COVID-19 global pandemic created a worldwide shortage of personal protective equipment (PPE)[1] and highlighted significant usability issues in current PPE products.[2] In addition to direct contact, airborne diseases may be spread by aerosol or droplet transmission. Aerosol transmission may be mitigated by the appropriate use of respiratory protective equipment (RPE), a particular classification of personal protective equipment (PPE). However, respiratory protective equipment is used as part of a hierarchy of control measures and is usually considered a last resort. This is because RPE only protects individual workers, is prone to failure or misuse (wrong RPE for the wrong task/environment) and wearers may get a false sense of security, encouraging risk-taking behaviours.[3] A range of inspiratory filtering devices exist: dust masks, half-face masks, full-face masks and powered (fan-assisted) respirators. Powered respirators include: half/full-face masks, helmets, hoods and visors. Though not used in healthcare, for completeness, breathing apparatuses are systems that supply an independent, positive pressure supply of breathing-quality air.

Face masks may be classified by considering the level of protection they offer the wearer to inhalation of environmental contaminants. Simple surgical face masks or 'nuisance' dust masks do not entirely filter droplets or aerosols. Filtering face piece (FFP) masks comprise layers of synthetic non-woven material with interleaved filtration layers and provide protection against small airborne particles (aerosols). Different types and constructions of FFP masks can be classified by their ability to filter small particles. Particulate filters can be classified as low (P1) to high (P3) efficiency, filtering between 80% of particles smaller than 2 micrometres to 99.95% of particles smaller than 0.5 micrometres, respectively (Table 1).[4] Respiratory protection can therefore be considered in terms of a combination of the filtering ability of the device relative to the exposure environment and its fit on the wearer's face. A device is considered adequate if it has the capacity to reduce the wearer's exposure to a hazardous substance to acceptable levels (to comply with occupational exposure limit values). Devices can be reusable, but the majority are single-use. Masks are difficult to recycle due to their layered construction and the pandemic contributed to an unprecedented rise in RPE-related clinical waste.[5]

The majority of RPE used in healthcare settings are disposable face masks adopted from industry. Masks are not designed to be worn for long periods or repeated shifts, may restrict vision and communication, may cause facial damage due to their tight fit, and require multiple time-consuming 'fit tests' for each model of the device for each staff member. All these issues were highlighted in the context of the 2002-2004 SARS epidemic.[6] More appropriate solutions for prolonged and repeated use include powered air-purifying respirators (PAPRs). But, again, these are not designed primarily for healthcare, are heavy, noisy, expensive, difficult to clean to clinical standards, and not suitable for the specific needs in frontline healthcare environments.

There have been several widely reported 'homemade' or 'MacGyvered' devices that well-intentioned groups or individuals developed to protect staff and patients during the pandemic.[7] None of these devices sought or achieved independent certification or provided data to support safety.[8] Turner and colleagues proposed a framework for the safer adoption of novel devices[7] which: defines the problem and reviews existing solutions, benchmarks safety indices for the devices, and then evaluates it in a structured manner through simulated, low- and then high-risk clinical settings Table S1 (Supplemental). Broad stakeholder feedback is encouraged through iterative review cycles, re-design and improvements.

For peer review only

**Table 1.** Classification of particulate filters, with a worked example and fit testing.

**P1** – Filters about 80% of particles smaller than 2 micrometres
**P2** – Filters about 94% of particles smaller than 0.5 micrometres
**P3** – Filters about 99.95% of particles smaller than 0.5 micrometres

For example: an airborne dust contaminant with an occupational exposure limit of 5mg/m$^3$ may be present in the workplace in concentrations up to 60mg/m$^3$ (determined by monitoring). A particle filter is needed to reduce the concentration by at least a factor of 12 (60/5=12). A P3 filter with an assigned protection factor of 20 would be suitable (as this is greater than the factor of 12 required). Other considerations such as exposure time, useability and disposal of the device need to be considered prior to undertaking a **fit test** with the intended wearer.

A fit test verifies that a **specific model** of device works as intended with a **particular individual**. For example, different face shapes and facial hair can interfere with a particular system's ability to filter environmental contaminants effectively.

**Qualitative fit testing** assesses the leakage past a mask of airborne compounds detectable by the wearer (typically bitter/sweet tasting substances), aerosolised using a spray device.

**Quantitative fit testing** measures particulate concentrations inside and outside of devices, typically undertaken by measuring sodium chloride aerosolised in water to generate a 'particle' count.

The ratio of airborne particles outside:inside the filtering device gives a **protection factor**. An **assigned protection factor** reflects the workplace conditions.

Considering the above, our project aimed to design and produce a low-cost, ergonomic, hood-integrated PAPR for use in frontline healthcare settings. Our objectives were to focus on user-centred design, engineering optimisation, staged feasibility testing, certification, intellectual property protection and then rapid manufacture and distribution. We also aimed to design the PAPR to be re-used, refurbished and recycled where possible, using readily available, simple and interchangeable key parts which proved difficult to source during the early stages of the pandemic. Finally, by designing an available, affordable PAPR system that could be cleaned appropriately and re-used between different staff, we aimed to provide equitable access to high-quality RPE that offered optimal protection to *all* staff, wherever they worked.[9] We hypothesised that participants would rate Bubble-PAPR more highly than current FFP3 face mask RPE across the domains of comfort, perceived safety and communication.

For peer review only

## Methods

The design team brought together frontline clinical staff based in the Wythenshawe Hospital Acute Intensive Care Unit (ICU) of Manchester University NHS Foundation Trust (MFT), an experienced product design consultancy (Designing Science Limited, Middlesex, UK) and the technical expertise of the School of Engineering at the University of Manchester (UoM). Research Ethical and Health Research Authority approval (IRAS ID:288493, REC Ref:21/WA/0018) was granted. The study was sponsored by MFT, who acted as the manufacturer of this in-house prototype device, which became known as Bubble-PAPR. The study protocol, analysis plan and recruitment metrics were registered and reported at ClinicalTrials.gov (NCT04681365). User needs assessment was conducted through a series of workplace diary card exercises documenting typical activities undertaken by frontline healthcare staff, synthesised in focus groups. Staff were invited to participate (by email and posters in rest areas) from clinical locations where RPE was mandated within the hospital. The first two respondents from each area were recruited to the diary card and focus group activities. Rapid design and evaluation cycles occurred based on the identified user needs. In addition, evaluation of early prototypes occurred in simulated clinical environments, collecting usability data from participants.

### Patient and public involvement

Public and Patient involvement was undertaken through the Manchester Academic Critical Care research group's patient forum. There were powerful accounts from patients who regularly described not being able to understand what hospital staff wearing PPE were saying and being troubled that they had no idea what their carers looked like. These reports led us to focus on prioritising the ease of communication with Bubble-PAPR. Staff participants who were invited to wear Bubble-PAPR were again recruited from clinical locations where RPE was mandated, by direct invitation from the research team. All relevant staff working in the area were approached until a maximum of six staff had been recruited per shift (the most that the research team could reasonably accommodate per shift), or the recruitment target had been met.

A Trial Safety Committee was established to oversee the results of laboratory and bench testing of the prototype, initial safety data, usability and adverse event data at each stage of the evaluation. The Committee met prior to commencing clinical evaluation. It was tasked with the decision to allow the evaluation to proceed between phases: simulated clinical environment, low-risk (non-infectious) clinical environment and high-risk clinical environment (COVID-19 wards and ICUs). A final iteration of Bubble-PAPR was further tested in high-risk environments. Prior to first use, several device safety checks were independently undertaken by the MFT Electrical and Biomedical Engineering Department and INSPEC International Ltd, Salford, UK). A short report based on the criteria detailed in Table S2 (Supplemental) was presented to the Trial Safety Committee. The first ten study participants to wear Bubble-PAPR underwent 'fit testing' with a particulometer (TSI Portacount Fit Tester 8040, TSI Instruments Ltd, Buckinghamshire, UK) following a standard protocol derived from the UK Government's Health and Safety Executive.[12] This INDG-479 protocol requires a 'Fit Factor' pass level of 100 for FFP3/N95 face masks and 500 for full face masks/hoods. European Conformity Standard EN12941 requires an applied fit factor of 40 for a 'loose-fitting hood' PAPR; the equivalent of a nominal protection factor of at least 500 (accepting an inward leakage of 0.2% with a P3 class filter. See Table 1). By comparison, the minimal fit factor for an FFP3 mask in a clinical environment is 100.


For peer review only - http://bmjopen.bmj.com/site/about/guidelines.xhtml

Tests were conducted in an ICU side room with a particle generator to reach background counts between 70,000 to 100,000 particles/cm$^3$.

The primary outcome was based on Davis' technology acceptance model (perceived usefulness and perceived ease-of-use overcoming barriers to adoption)[10]. First, staff were asked to rate their experiences using current RPE (a variety of re-useable or disposable FFP3 masks) using a series of questions based on Likert-type scales (see Supplemental material). Next, safe use of the Bubble-PAPR was explained, and instructions for use were provided, supported by videos of donning, doffing, cleaning and storage. Bubble-PAPR was then worn during simulated/clinical use where the usual tasks (identified in the focus groups) were undertaken. Finally, after removal (doffing) of Bubble-PAPR, staff were immediately invited to complete a second questionnaire focused on the prototype. Free text comments were also invited.

The primary endpoint was staff rating of the comfort of Bubble-PAPR (versus current FFP3 face masks). Secondary endpoints focused on communication and perceived safety. Specifically, this was staff ratings of the prototype in terms of: how safe participants felt, ease of communication with colleagues, and ease of communication with patients (again, Bubble-PAPR versus current FFP3 face masks). In parallel, in-house device feasibility testing was conducted in the hospital environment to test ergonomics and air particle filtration. We tested against existing conformity standards for PAPRs relevant at the time of development (British Standard BS EN 12941 [Respiratory Protective Devices: Powered filtering devices incorporating a helmet or hood] and the European Union Personal Protective Equipment Directive EU2016/425). [4 11]

A pilot evaluation was conducted in August 2020 to test the questionnaires and to assess the likely population means for the test scores (Table S3 Supplemental). We calculated a sample size of 20 participants would be required for each phase of the evaluation to detect a significant difference between usual PPE and Bubble-PAPR, based on a mean difference of 2.5 (SD 0.9) points on the 7-point Likert scale identified during the pilot evaluation (alpha = 0.05, 90% power). In addition, we allowed for a 5% dropout and missing data rate, concluding 22 participants per phase. All variables were explored via appropriate graphical and descriptive statistics for completeness and form. Analyses were conducted in RStudio 2020 (Boston, MA, www.rstudio.com). Analyses were performed separately for each phase for presentation to the Trial Safety Committee, with a pooled analysis conducted at the study conclusion. Comparisons between groups (current RPE vs Bubble-PAPR) were made using a paired t-test or Wilcoxon signed-rank test as appropriate.


For peer review only - http://bmjopen.bmj.com/site/about/guidelines.xhtml

## Results

The final design of Bubble-PAPR is shown schematically in Figure 1 (www.bubble-papr.com). The device safety checks and fit testing results are presented in Tables S2, S3 and S4 (Supplemental), respectively, demonstrating a mean fit factor of 16,961. Additional particulometer tests were undertaken with deliberate tears up to 20 cm in the hood using a dummy head. The lowest fit factor recorded with the damaged hood was 1,123. Therefore, the Trial Safety Committee concluded that the Bubble-PAPR performed its primary purpose of adequately protecting staff from airborne environmental contaminants.

Fifteen staff contributed to the diary and focus group exercises, generating a list of tasks to be undertaken. One staff member from the 16 invited could not attend the focus group meeting. Ninety-one staff wore Bubble-PAPR for a median of 45 (IQR 30-90, range 10-150) minutes between 3rd March and 21st December 2021. No staff who were approached during their clinical shifts were unwilling or unable to trial Bubble-PAPR. There were no Bubble-PAPR-related safety incidents reported during the study. Staff undertook all clinical duties identified by the focus groups and diary card exercise, either in the simulation suite (n=22) or clinical settings (n=22 low-risk, n=25 high-risk, n=22 high-risk with final iteration). Participants predominantly declared as female (69%), and were from a range of clinical and non-clinical roles, Figure S1 (Supplemental). Staff self-reported a range of heights (mean 1.7m [SD 0.1, range 1.5-2.0]), weights (72.4kg [16.0, 47-127]) and body mass indices (25.3 [4.7,16.7-42.9]), Figure S2 (Supplemental). Fifty-two percent of participants reported that they normally wore glasses, with 31% wearing glasses during the evaluation. All participants described at least 6 month's experience with FFP3 face masks on a regular basis ("most shifts"), with a combination of re-useable (typically 3M™ 6000 Series Respirators) and single use (typically 3M™ Aura™ 9330 or equivalent) face masks. No participants described using PAPRs in the six months prior to recruitment. All participants completed all mandatory questionnaire sections.

With pooled data for the primary outcome, "*How comfortable do you feel in your PPE?*" (Likert scale bounded by 1 [very uncomfortable] to 7 [very comfortable]), Bubble-PAPR mean score was 5.64 (SD 1.55) versus usual FFP3 face mask 2.96 (1.44), Figure 2. There was a mean difference of 2.68 (95% CI 2.23-3.14, $p<0.001$). Secondary outcomes focused on communication and perceived safety. For the question, "*How safe do you feel in your PPE?*", Bubble-PAPR mean score was 6.15 (0.94) vs usual FFP3 face mask 5.43 (0.98); mean difference 0.73 (95% CI 0.45-1.00, $p<0.001$), Figure 2. Figure 3 demonstrates communication outcomes for all 91 comparisons of Bubble-PAPR versus usual FFP3 face masks. All adjusted comparisons were significant ($p<0.001$) in favour of Bubble-PAPR for communicating with both colleagues and patients (Table 2).

Secondary outcomes where a lower Likert response was considered better are presented in Figure S3 (Supplemental). These focussed on whether staff were worried about themselves or others whilst wearing RPE, whether the devices caused pressure or pain or if communication was impaired. Finally, staff were asked if they had to cut short a clinical (or simulated) encounter due to discomfort with their RPE. Again, there was a significant difference in favour of Bubble-PAPR for all metrics (all $p<0.001$, Table 2).


For peer review only - http://bmjopen.bmj.com/site/about/guidelines.xhtml

**Table 2.** Rating scales, summary results and comparisons across the questionnaire domains.

| | PPE | Q1 Confidence in donning | Q2 Confidence in donning without dislodging other PPE | Q3 Wear with glasses/goggles | Q4 Protect yourself from respiratory infection | Q5 Protect patient from infection from you | Q6 Safely care for your patient | Q7 Safely roll patient | Q8 Speak to staff | Q9 Be heard by staff | Q10 Speak to patient | Q11 Be heard by patient | Q12 Doff safely | Q13 Doff without dislodging glasses |
|---|---|---|---|---|---|---|---|---|---|---|---|---|---|---|
| Rating scale | From: | 0 - no confidence | 0 - no confidence | 0 - no confidence | 0 - no confidence | 0 - no confidence | 0 - no confidence | 0 - no confidence | 0 - no confidence | 0 - no confidence | 0 - no confidence | 0 - no confidence | 0 - no confidence | 0 - no confidence |
| | To: | 10 - fully confident | 10 - fully confident | 10 - fully confident | 10 - fully confident | 10 - fully confident | 10 - fully confident | 10 - fully confident | 10 - fully confident | 10 - fully confident | 10 - fully confident | 10 - fully confident | 10 - fully confident | 10 - fully confident |
| | | | | | | | | | | | | | | |
| RPE type | FFP3 | 8.9 (1.4) [3 - 10] | 8.3 (2) [2 - 10] | 6.9 (2.6) [2 - 10] | 8.2 (1.6) [4 - 10] | 8.2 (1.7) [2 - 10] | 8.4 (1.4) [5 - 10] | 8.2 (1.8) [2 - 10] | 5.1 (2.4) [1 - 10] | 4.9 (2.3) [1 - 10] | 4.8 (2.4) [1 - 10] | 4.7 (2.5) [1 - 10] | 8.1 (1.9) [2 - 10] | 6.2 (2.5) [1 - 10] |
| | Bubble | 7.4 (1.8) [3 - 10] | 7.7 (1.8) [2 - 10] | 7.6 (1.9) [3 - 10] | 8.6 (1.6) [3 - 10] | 8.5 (1.8) [2 - 10] | 8.0 (2) [2 - 10] | 7.8 (2.2) [2 - 10] | 7.5 (2.4) [1 - 10] | 7.1 (2.3) [1 - 10] | 7.8 (2.1) [2 - 10] | 7.4 (2.4) [1 - 10] | 8.0 (1.8) [2 - 10] | 7.8 (1.9) [2 - 10] |
| Comparison | Mean difference | -1.48 | -0.55 | 0.7 | 0.43 | 0.3 | -0.42 | -0.42 | 2.38 | 2.16 | 2.99 | 2.7 | -0.1 | 1.66 |
| | 95% CI | -1.9 to -0.99 | -1.12 to 0.02 | -0.00 to 1.40 | -0.04 to 0.89 | -0.18 to 0.78 | -0.91 to 0.07 | -0.98 to 0.15 | 1.66 to 3.11 | 1.45 to 2.88 | 2.36 to 3.62 | 1.97 to 3.433 | -0.63 to 0.43 | 0.98 to 2.34 |
| | | Favours FFP3 | No difference | Favours Bubble | No difference | No difference | No difference | No difference | Favours Bubble | Favours Bubble | Favours Bubble | Favours Bubble | No difference | Favours Bubble |
| | Adjusted p | <0.001 | 0.058 | 0.049 | 0.070 | 0.217 | 0.092 | 0.144 | <0.001 | <0.001 | <0.001 | <0.001 | 0.711 | <0.001 |

| | PPE | Q14 How safe does it feel | Q15 Worried about own health | Q16 Worried others health | Q17 Comfortable | Q18 Don ease | Q19 Doff ease | Q20a Restricted communication | Q20b Vision distorted with Bubble | Q20c Read monitors, computers, and notes with Bubble | Q21 Pressure marks on head/face | Q22 Pain on head/face | Q23 Leave clinical area early due to RPE |
|---|---|---|---|---|---|---|---|---|---|---|---|---|---|
| Rating scale | From: | 1 - very unsafe | 1 - not worried at all | 1 - not worried at all | 1 - very uncomfortable | 1 - not at all easy | 1 - not at all easy | 1 - not at all restricted | 1 - not at all affected | 1 - clear at all times | 1 - never | 1 - never | 1 - never |
| | To: | 7 very safe | 7 - very worried | 7 - very worried | 7 - very comfortable | 7 - very easy | 7 - very easy | 7 - very restricted | 7 - very affected | 7 - not clear at all | 7 - always | 7 - always | 7 - always |
| | | | | | | | | | | | | | |
| RPE type | FFP3 | 5.4 (1) [3 - 7] | 3.2 (1.5) [1 - 7] | 3.2 (1.5) [1 - 7] | 3 (1.4) [1 - 6] | 4.9 (1.3) [2 - 7] | 5.1 (1.3) [2 - 7] | 5.4 (1.4) [1 - 7] | - | - | 5.8 (1.4) [1 - 7] | 5.3 (1.4) [1 - 7] | 3.6 (1.6) [1 - 7] |
| | Bubble | 6.2 (0.9) [3 - 7] | 2.3 (1.6) [1 - 7] | 2.3 (1.7) [1 - 7] | 5.6 (1.6) [1 - 7] | 5.5 (1.4) [2 - 7] | 5.7 (1.2) [2 - 7] | 3.9 (1.7) [1 - 7] | 3.2 (1.9) [1 - 7] | 5.6 (1.5) [2 - 7] | 1.3 (0.8) [1 - 6] | 1.4 (0.9) [1 - 6] | 1.5 (1) [1 - 6] |
| Comparison | Mean difference | 0.73 | -0.92 | -0.93 | 2.68 | 0.62 | 0.63 | -1.49 | - | - | -4.54 | -3.99 | -2.13 |
| | 95% CI | 0.45 to 0.99 | -1.36 to -0.49 | -1.36 to -0.48 | 2.23 to 3.14 | 0.21 to 1.02 | 0.26 to 0.99 | -1.95 to -1.04 | - | - | -4.90 to -4.17 | -4.35 to -3.63 | -2.51 to - 1.75 |
| | | Favours Bubble | Favours Bubble | Favours Bubble | Favours Bubble | Favours Bubble | Favours Bubble | Favours Bubble | No comparator | No comparator | Favours Bubble | Favours Bubble | Favours Bubble |
| | Adjusted p | <0.001 | <0.001 | <0.001 | <0.001 | 0.003 | 0.002 | <0.001 | - | - | <0.001 | <0.001 | <0.001 |

For peer review only



For peer review only - http://bmjopen.bmj.com/site/about/guidelines.xhtml

During the initial phases, there was no significant difference between staff reporting ease of donning and doffing of Bubble-PAPR and usual PPE (which staff had used for many months at the time of the evaluation). However, pooled results saw staff becoming more familiar with the Bubble, and Bubble-PAPR was rated easier to don and doff when compared with usual FFP3 face masks (adjusted p=0.003 and 0.002 respectively), Table 2 and Figure S4 (Supplemental). Free text comments were reviewed and categorised into positive, negative and neutral comments (Figures S5-7 Supplemental). Most comments focused on the noise of the device, which improved throughout the project as the impellor and motor were made quieter in later design iterations.

For peer review only


For peer review only - http://bmjopen.bmj.com/site/about/guidelines.xhtml

**Discussion**

Our project developed an innovative prototype PAPR explicitly designed for prolonged healthcare use in high-risk clinical environments. Bubble-PAPR achieved its primary purpose of protecting staff from airborne potentially infectious material whilst also being rated significantly higher for comfort (the primary outcome), perceived safety, and communication with colleagues and patients (secondary outcomes) than usual RPE. Bubble-PAPR was used in all relevant simulated and clinical scenarios identified by detailed staff diary cards, making the results of this study extremely relevant to hospital-based healthcare workers.

Bubble-PAPR was rapidly developed based on the lived experiences of frontline staff during the early stages of the coronavirus pandemic, addressing the unmet needs of reliable, high-quality, universal and available RPE with improved comfort and communication when compared to usual FFP3 face masks. Staff overwhelmingly recognised the importance of facial visualisation when communicating with colleagues and patients. When combined with the improved comfort of wearing a PAPR over usual RPE, participants rated Bubble-PAPR consistently highly across all comparator domains.

This relatively simple evaluation study was preceded by a rapid design and prototyping phase, producing a working prototype within a few weeks. Despite the speed and agility demonstrated by the design team, we adhered to relevant standards, following a tiered evaluation within the governance structure of an approved and regulated research project. Bubble-PAPR was only introduced into higher-risk environments following review by the Trial Safety Committee. This approach contrasted with many rapidly developed or adopted RPE systems that became prevalent during the pandemic, often disseminated via social media and almost always without any meaningful safety or useability data.[7, 13, 14] Whilst the PPE shortages experienced during the pandemic drove many of these innovations and adaptations, we recognised the importance of a methodical approach to design, development and testing of our prototype, both in the laboratory and clinical settings. We recommend others to follow the framework proposed by Duggan et al. for the development of novel medical devices, with regular reviews of safety and useability data within the framework of a robust and transparent clinical trial.[7]

Our study has some limitations. The design of Bubble-PAPR addressed many of the issues identified by the same staff who subsequently evaluated the prototype. Whilst our study protocol allowed evaluation only within our Trust owing to the ‘in-house’ manufacturing exemption for testing, it is not unreasonable to expect similar results if our prototype were evaluated elsewhere. Although this may be considered a weakness of the study, many of the shortcomings of the PPE provided to frontline health workers around the world are well described and are essentially the same as those identified in our project.[15, 16] Furthermore, we evaluated Bubble-PAPR against single-use and reusable FFP3 face masks, which could be construed as comparing two different classes of RPE. However, Bubble-PAPR was designed and developed to provide a viable alternative to FFP3 class face masks, in contrast to the more usual healthcare use of PAPRs – selectively available on a limited basis to specific users or groups. Our detailed analysis of work diary cards from various clinical staff ensured that Bubble-PAPR was used for all relevant procedures undertaken by medical, nursing, healthcare assistant, allied healthcare professional (speech and language therapy, physiotherapy, pharmacy), administrative and


For peer review only - http://bmjopen.bmj.com/site/about/guidelines.xhtml

domestic staff in the clinical area. This perspective is unique within respiratory protective equipment product evaluation studies.[17, 18]

Our study did not directly evaluate the patient experience with staff wearing different RPE. However, the patient experience was reflected in the user specifications identified around communication, and anecdotal feedback was positive from patients, especially around facial visibility and verbal and non-verbal communication. In addition, when contrasted with FFP3 face masks, speech and language therapists reported that demonstrating speech and swallow exercises was suddenly possible with Bubble-PAPR and that the transparent nature of the hood overcame the communication barriers that can be so devastating for those with hearing impairments.[19] Although designed to be potentially recyclable, future work should address the environmental impact of PVC hoods with reusable collars compared to single-use or reusable FFP3 face masks.

For peer review only


For peer review only - http://bmjopen.bmj.com/site/about/guidelines.xhtml

**Conclusions**

Our study has demonstrated that Bubble-PAPR achieved its primary purpose of keeping staff safe from airborne particulate material whilst improving comfort, communication and the user experience. It is likely that the patient experience was also enhanced. Bubble-PAPR has been patented (PCT/GB2021/052147) and subsequently licenced to GAMA Healthcare Ltd (Hemel Hempstead, UK) for large-scale manufacture and distribution as RediHood™ to frontline NHS and other workers. The pandemic drove unprecedented collaboration between clinicians, academics and industry. Bubble-PAPR is an excellent example of developing a cosmopolitan network that could become a key feature of future system resilience. The design and development of Bubble-PAPR were conducted using a careful strategy addressing key regulatory and safety steps, in contrast to many devices rapidly developed and deployed during the pandemic.

For peer review only


For peer review only - http://bmjopen.bmj.com/site/about/guidelines.xhtml

**Acknowledgements**

We are grateful to our funders (detailed below) for supporting this project and the staff who participated in the evaluation. In addition, we are indebted to the designers, engineers and staff who gave their time freely during the early stages of the coronavirus pandemic to work tirelessly on designing, building, testing and refining Bubble-PAPR. Specifically:

Patrick Hall, Designing Science Ltd (www.designingscience.co.uk)
Andrew Spragg, Industrial design consultant
Andrew Forbes, XK Design Ltd
James Corden, Manchester University Hospital NHS Foundation Trust Innovation Team
Nick Duggan, Innovation Consultant, Zuas (www.zuas.io)
GAMA Healthcare Ltd, Hemel Hempstead, UK (www.gamahealthcare.com)

**Conflict of interests**

Manchester University NHS Foundation Trust, the University of Manchester and Designing Science Ltd have agreed commercial terms to license Bubble-PAPR to GAMA Healthcare Ltd for manufacture and development, branded as RediHood™.

**Funding**

This project was supported by unrestricted grants and funding from:

- Engineering and Physical Sciences Research Council (EPSRC) Impact Acceleration Account 302
- Oxford Road Corridor (no relevant grant award number)
- Health Innovation Manchester 'Momentum' special projects fund 2021
- Acute ICU Charitable Research Fund, Manchester University NHS Foundation Trust (no relevant grant award number)
- Manchester University NHS Foundation Trust (no relevant grant award number)

**Data availability statement**

Due to the commercial sensitivity of the intellectual property licensed at the conclusion of this project, the full dataset is not publicly available. However, the corresponding author will consider requests to disclose the dataset on an individual basis if necessary.

**Trial registration**

Research Ethical and Health Research Authority approval (IRAS ID:288493, REC Ref:21/WA/0018). Manchester University NHS Foundation Trust sponsored the study, acting as the manufacturer of this in-house prototype device. The study protocol, analysis plan and recruitment metrics were registered and reported at ClinicalTrials.gov (NCT04681365).

For peer review only


For peer review only - http://bmjopen.bmj.com/site/about/guidelines.xhtml

## Author contributions

All authors critically revised the manuscript for important intellectual content and approved the final manuscript. BAM attests that all listed authors meet authorship criteria and that no others meeting the criteria have been omitted.

**BAM:** conception and design, collection, analysis and interpretation of data, drafting and revision of the manuscript, and final approval of the version to be published. Participant recruitment.

**CS:** qualitative work package conception and design, analysis and interpretation of data. Participant recruitment. Drafting and revision of the final manuscript, and final approval of the version to be published.

**AG:** qualitative work package design, analysis and interpretation of data. Drafting and revision of the final manuscript, and final approval of the version to be published.

**RC:** design, collection, and interpretation of data, drafting and revision of the manuscript, and final approval of the version to be published.

**JL:** design, collection, and interpretation of data, drafting and revision of the manuscript, and final approval of the version to be published.

**PGA:** design, collection, and interpretation of data, drafting and revision of the manuscript, and final approval of the version to be published.

**GC:** design, collection, and interpretation of data, drafting and revision of the manuscript, and final approval of the version to be published. Manufacturing and engineering lead.

**Provenance and peer review**
Not commissioned; externally peer reviewed.

**Word count of main document:** 3111

## Ethics approval

Research Ethical and Health Research Authority approval (IRAS ID:288493, REC Ref:21/WA/0018) was granted from Wales REC5 on 27th January 2021. The study was sponsored by Manchester University NHS Foundation Trust, who acted as the manufacturer of this in-house prototype device. The study protocol, analysis plan and recruitment metrics were registered and reported at ClinicalTrials.gov (NCT04681365).

For peer review only


For peer review only - http://bmjopen.bmj.com/site/about/guidelines.xhtml

**Table and Figure legends**

**Table 1.** Classification of particulate filters, with a worked example and fit testing.

**Table 2.** Rating scales, summary results and comparisons across the questionnaire domains.

**Table S1. (Supplemental)** Proposed framework for the safer adoption of a MacGyvered device. Adapted from Turner and colleagues.[7]

**Table S2. (Supplemental)** Lab-based testing of the Bubble PAPR prior to clinical evaluation.

**Table S3. (Supplemental).** Pilot data. Q14 & Q17 are Likert Scale items (rated 1-7) and Q8-11 are Visual Analogue Scale items (rated 0-100).

**Table S4. (Supplemental)** Fit testing data from the first 10 participants.

**Figure 1.** Bubble-PAPR comprises a medical-grade foam neck collar and a separate PVC hood. The universal fit collar draws air in through a filter via an impellor powered by an external battery. The collar has a mechanical low flow indicator and can be cleaned and reused by different users. The semi-rigid hood is pulled over the collar before donning and is secured by integrated straps.

**Figure 2.** Reported comfort (primary) and safety (secondary) outcomes for Bubble-PAPR vs usual FFP3 face masks.

**Figure 3.** Secondary communication outcomes where a higher Likert scale response was considered better.

**Figure S1. (Supplemental)** Participant job roles.

**Figure S2. (Supplemental)** Self-reported weight, height and BMI of staff participants.

**Figure S3. (Supplemental)** Secondary outcomes where a lower Likert scale response was considered better.

**Figure S4. (Supplemental)** Ease of donning and doffing of Respiratory Protective Equipment.

**Figure S5. (Supplemental).** Word clouds from the free text feedback. Negative comments (all categories).

**Figure S6 (Supplemental).** Word clouds from the free text feedback. Neutral comments (all categories).

**Figure S7 (Supplemental).** Word clouds from the free text feedback. Positive comments (all categories).

For peer review only


For peer review only - http://bmjopen.bmj.com/site/about/guidelines.xhtml

## References


1. Fadela Chaib, World Health Organisation. Shortage of personal protective equipment endangering health workers worldwide. 3rd March 2020. Available from www.who.int/news/item/03-03-2020-shortage-of-personal-protective-equipment-endangering-health-workers-worldwide [Accessed 5th May 2020].

2. Hignett S, Welsh R, Banerjee J. Human factors issues of working in personal protective equipment during the COVID-19 pandemic. *Anaesthesia* 2021; 76: 134-135.

3. Cook TM. Personal protective equipment during the coronavirus disease (COVID) 2019 pandemic - a narrative review. *Anaesthesia* 2020; 75: 920-927.

4. British Standard BS EN12941 Respiratory protective devices - Powered filtering devices incorporating a helmet or a hood - Requirements, testing, marking (British Standard). 1999. Available from https://standards.globalspec.com/ [Accessed 5th April 2020].

5. Rizan C, Reed M, Bhutta MF. Environmental impact of personal protective equipment distributed for use by health and social care services in England in the first six months of the COVID-19 pandemic. *Journal of the Royal Society of Medicine* 2021; 114: 250-263.

6. Foo CC, Goon AT, Leow YH, Goh CL. Adverse skin reactions to personal protective equipment against severe acute respiratory syndrome--a descriptive study in Singapore. *Contact Dermatitis* 2006; 55: 291-294.

7. Turner MC, Duggan LV, Glezerson BA, Marshall SD. Thinking outside the (acrylic) box: a framework for the local use of custom-made medical devices. *Anaesthesia* 2020; 75: 1566-1569.

8. Gould CL, Alexander PDG, Allen CN, McGrath BA, Shelton CL. Protecting staff and patients during airway management in the COVID-19 pandemic: are intubation boxes safe. *British Journal of Anaesthesia* 2020; 125: e292-e293.

9. Shelton C, El-Boghdadly K, Appleby JB. The 'haves' and 'have-nots' of personal protective equipment during the COVID-19 pandemic: the ethics of emerging inequalities amongst healthcare workers. *Journal of Medical Ethics* 2021; medethics-2021-107501.

10. Davis FD. Perceived Usefulness, Perceived Ease of Use, and User Acceptance of Information Technology. *MIS Quarterly* 1989; 13: 319-340.

11. European Agency for Health and Safety at Work. Regulation (EU) 2016/425 on Personal Protective Equipment. 2016. Available from https://osha.europa.eu/en/legislation/directive/regulation-eu-2016425-personal-protective-equipment. [Accessed 5th April 2020].

12. Great Britain Health and Safety Executive. Guidance on Respiratory Protective Equipment (RPE) Fit Testing: (Rev1). March 2019. Available from https://www.hse.gov.uk/pubns/indg479.htm [Accessed 5th April 2020].

13. Duggan LV, Marshall SD, Scott J, Brindley PG, Grocott HP. The MacGyver bias and attraction of homemade devices in healthcare. *Canadian Journal of Anesthesia/Journal Canadien d'Anesthésie* 2019; 66: 757-761.

14. Addi RA, Benksim A, Cherkaaoui M. Easybreath Decathlon Mask: An Efficient Personal Protective Equipment (PPE) against COVID-19 in Africa. *Journal of Clinical and Experimental Investigations* 2020; 11: em00738.




For peer review only - http://bmjopen.bmj.com/site/about/guidelines.xhtml

15. Kim H, Hegde S, LaFiura C et al. Access to personal protective equipment in exposed healthcare workers and COVID-19 illness, severity, symptoms and duration: a population-based case-control study in six countries. *BMJ Global Health* 2021; 6: e004611.

16. Hoernke K, Djellouli N, Andrews L et al. Frontline healthcare workers' experiences with personal protective equipment during the COVID-19 pandemic in the UK: a rapid qualitative appraisal. *BMJ Open* 2021; 11: e046199.

17. Galanis P, Vraka I, Fragkou D, Bilali A, Kaitelidou D. Impact of personal protective equipment use on health care workers' physical health during the COVID-19 pandemic: A systematic review and meta-analysis. *American Journal of Infection Control* 2021; 49: 1305-1315.

18. Houghton C, Meskell P, Delaney H et al. Barriers and facilitators to healthcare workers' adherence with infection prevention and control (IPC) guidelines for respiratory infectious diseases: a rapid qualitative evidence synthesis. *Cochrane Database of Systematic Reviews* 2020; 4: CD013582.

19. Poostchi A, Kuet ML, Richardson PS, Patel MK. Covid-19: face masks can be devastating for people with hearing loss but alternatives are available. *BMJ* 2020; 370: m3326.

For peer review only


For peer review only - http://bmjopen.bmj.com/site/about/guidelines.xhtml

For peer review only


For peer review only - http://bmjopen.bmj.com/site/about/guidelines.xhtml

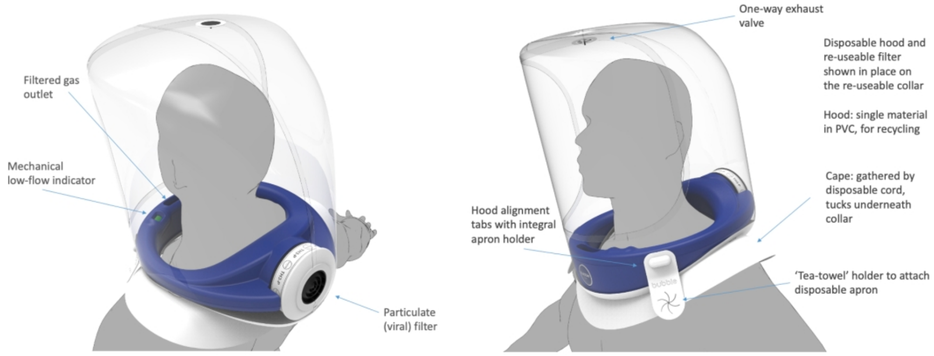


Bubble-PAPR comprises a medical-grade foam neck collar and a separate PVC hood. The universal fit collar draws air in through a filter via an impellor powered by an external battery. The collar has a mechanical low flow indicator and can be cleaned and reused by different users. The semi-rigid hood is pulled over the collar before donning and is secured by integrated straps.

762x283mm (72 x 72 DPI)

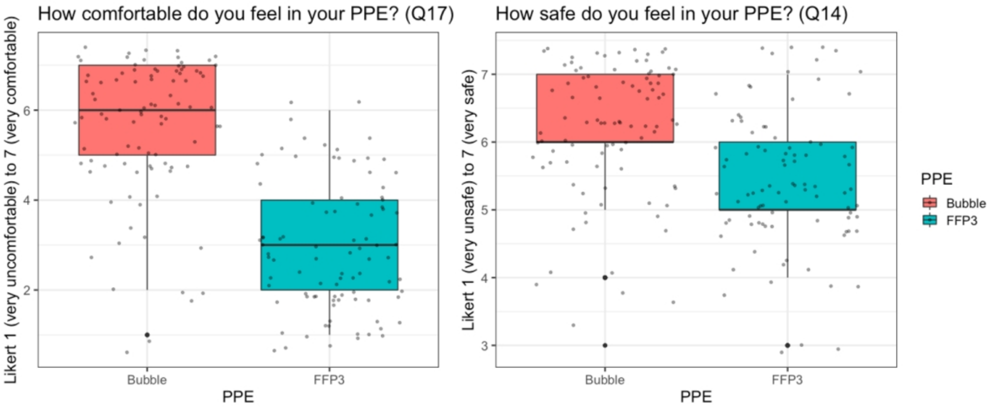


Reported comfort (primary) and safety (secondary) outcomes for Bubble-PAPR vs usual FFP3 face masks.

537x225mm (72 x 72 DPI)

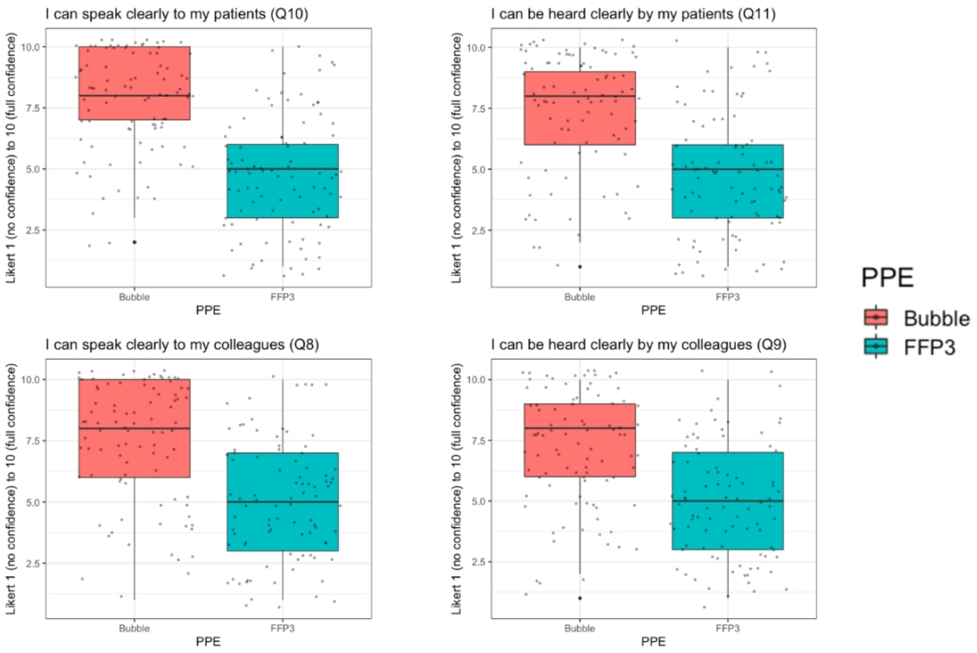


Secondary communication outcomes where a higher Likert scale response was considered better.

446x295mm (72 x 72 DPI)

Figure S1. (Supplemental) Participant job roles.

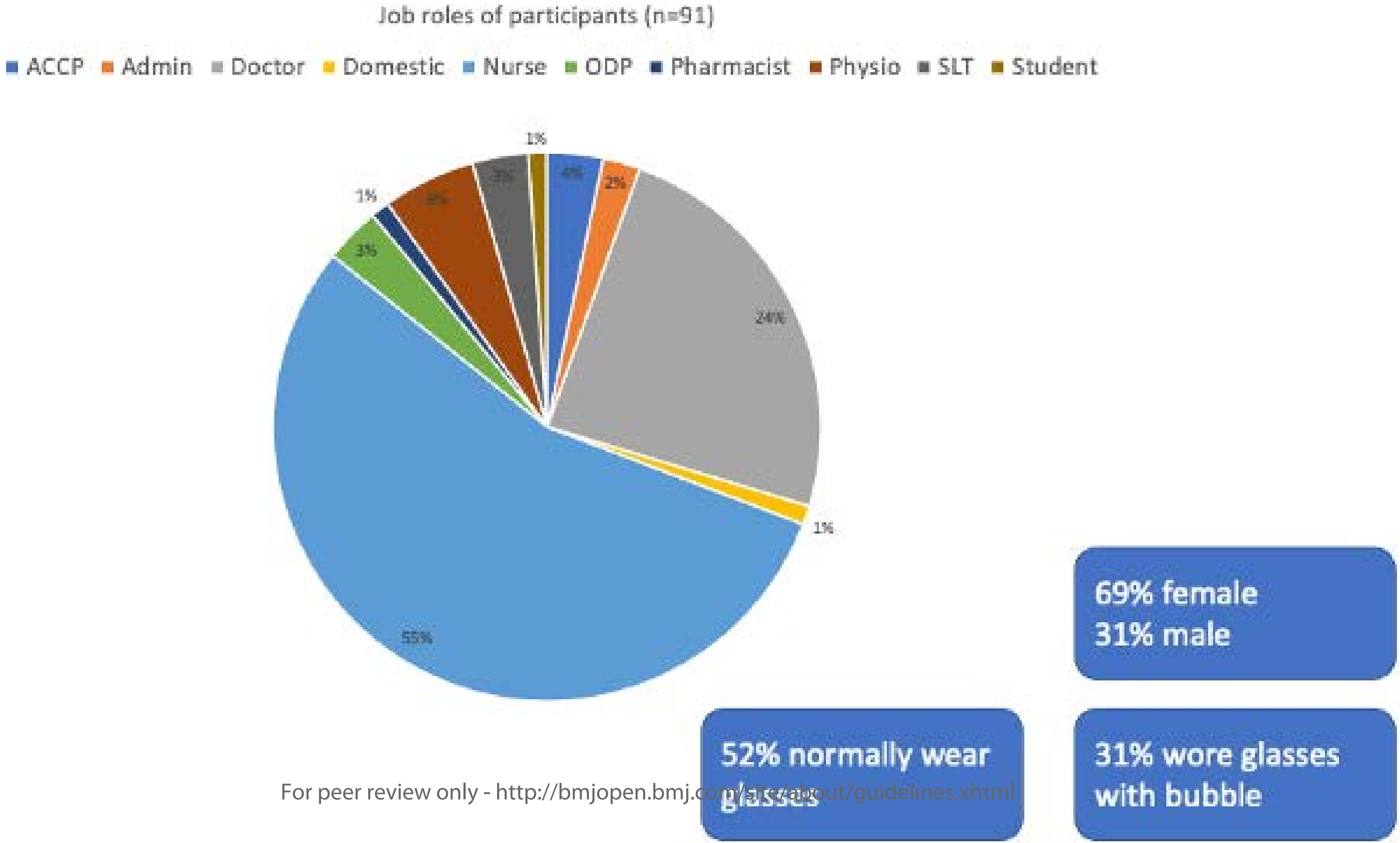

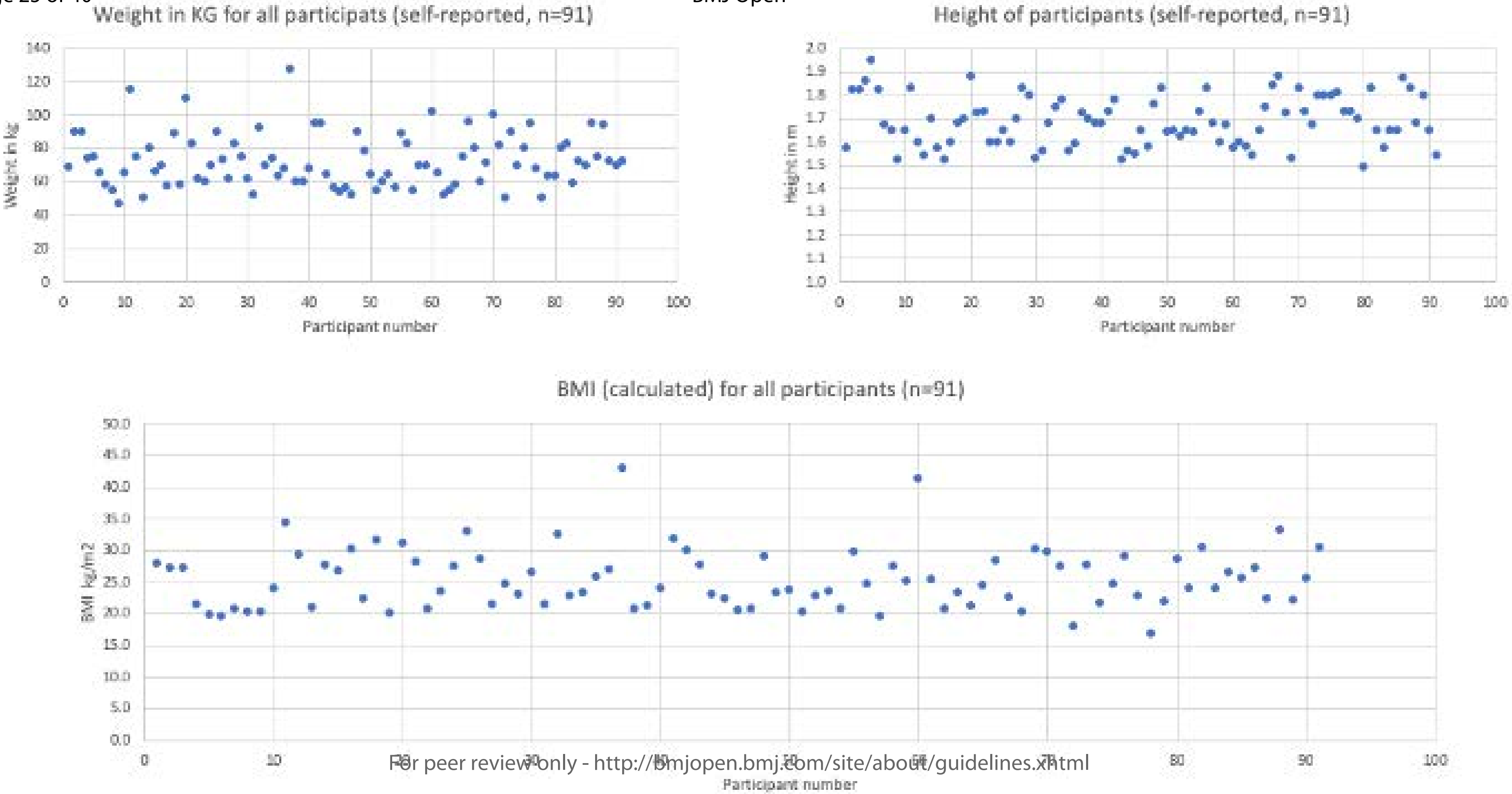


For peer review only - http://bmjopen.bmj.com/site/about/guidelines.xhtml

Figure S2. (Supplemental) Self-reported weight, height and BMI of staff participants.

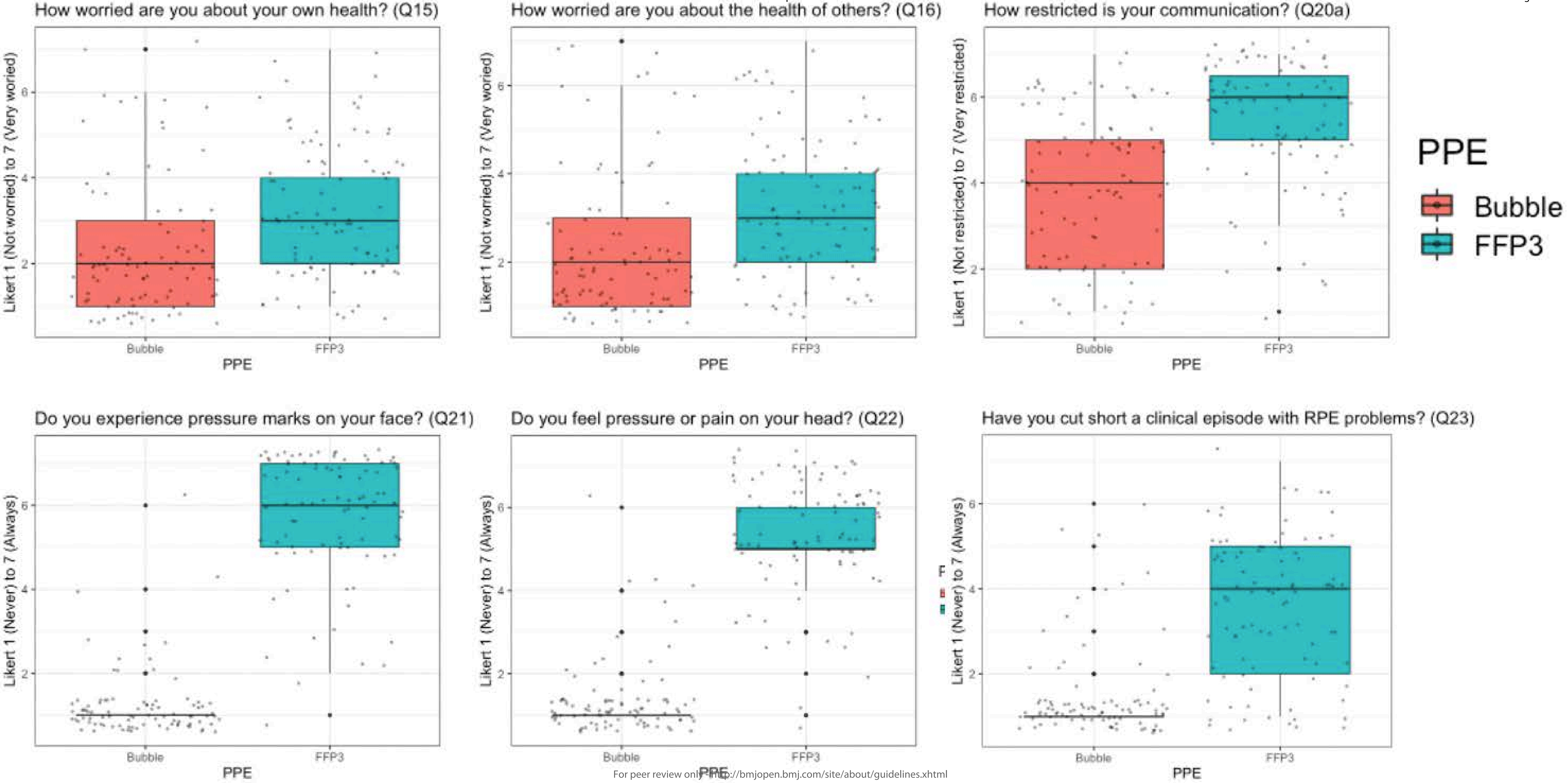




Figure S3. (Supplemental) Secondary outcomes where a lower Likert scale response was considered better.

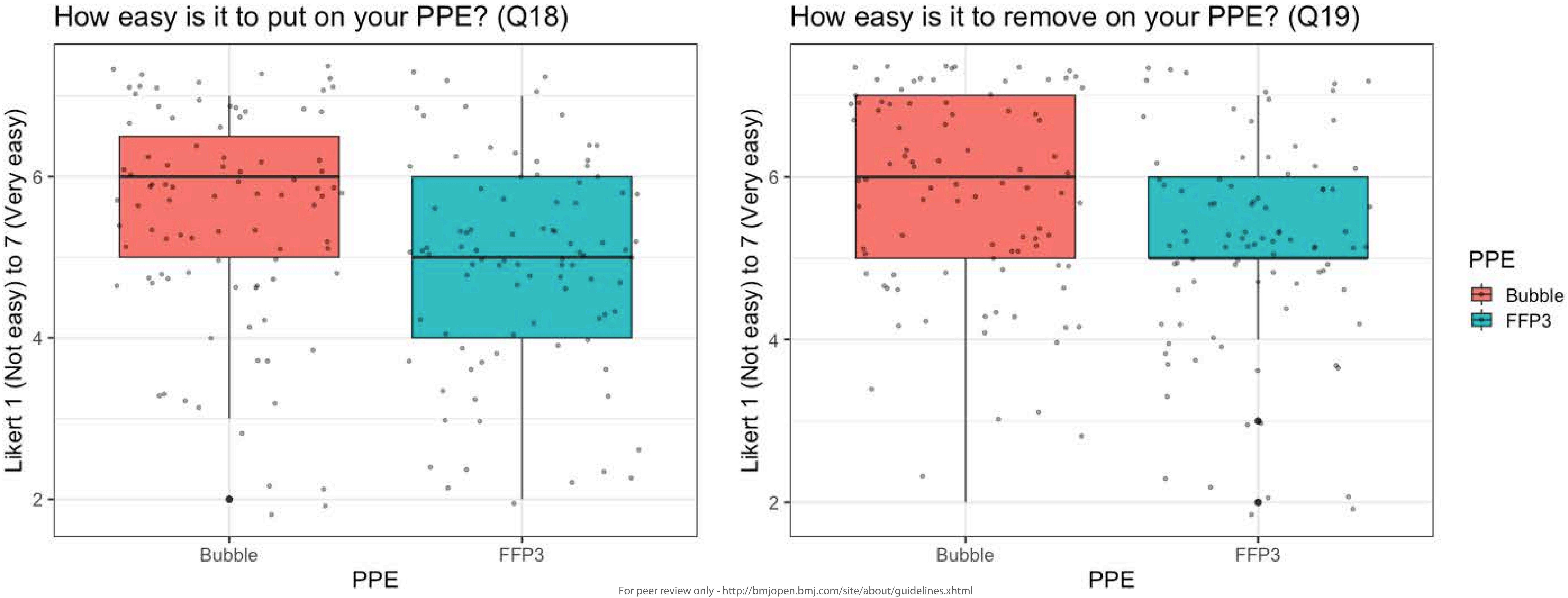


For peer review only - http://bmjopen.bmj.com/site/about/guidelines.xhtml

Figure S4. (Supplemental) Ease of donning and doffing of Respiratory Protective Equipment.

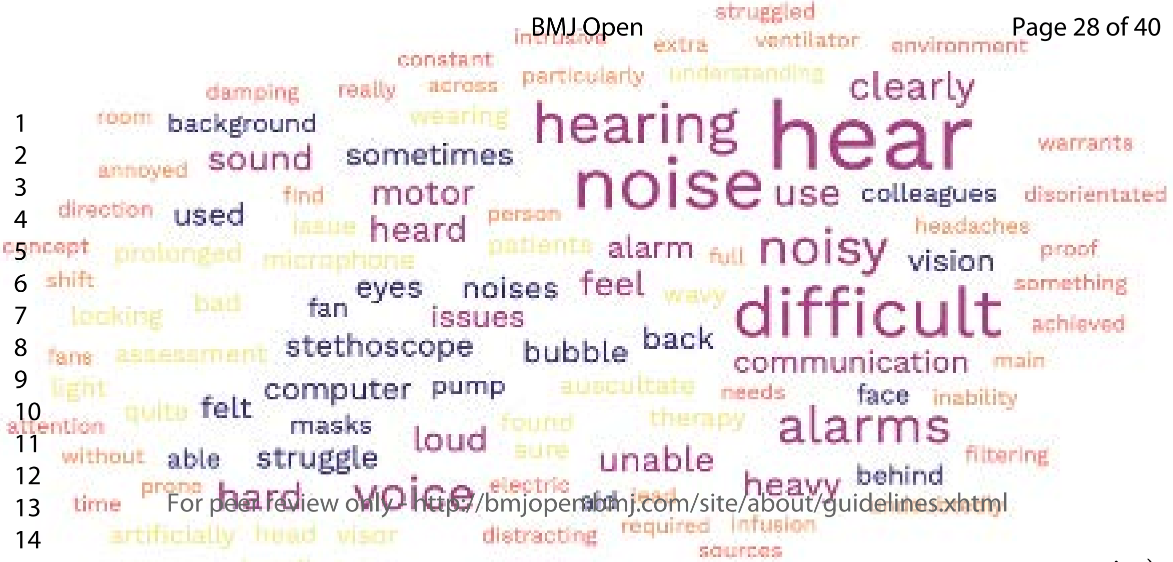




Figure S5. (Supplemental). Word clouds from the free text feedback. Negative comments (all categories).

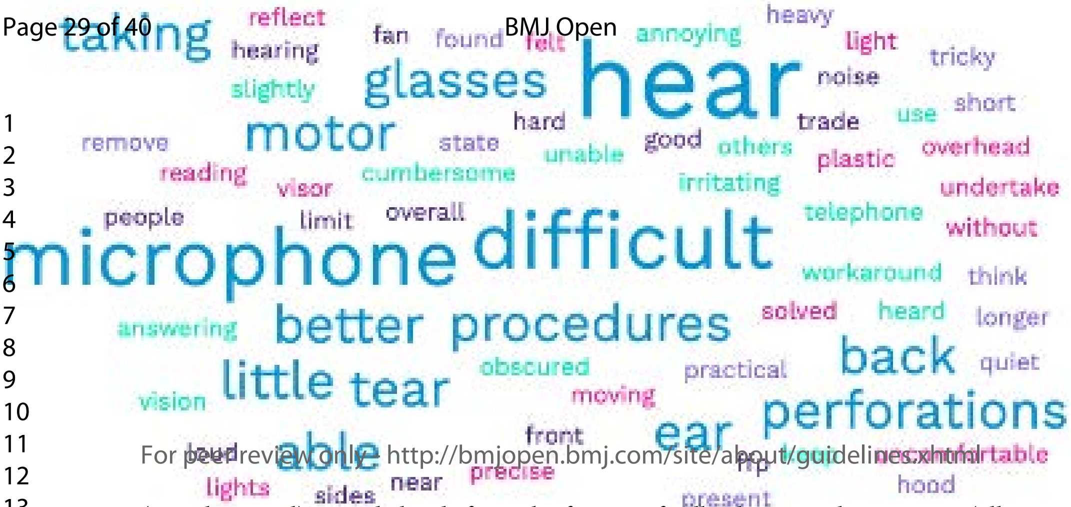


Figure S6 (Supplemental). Word clouds from the free text feedback. Neutral comments (all categories).

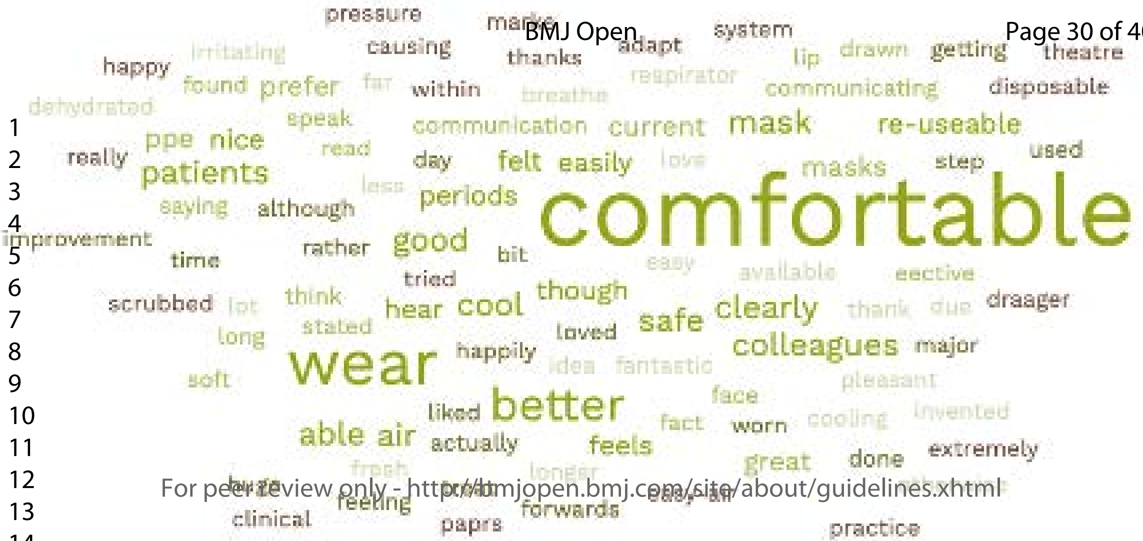


Figure S7 (Supplemental). Word clouds from the free text feedback. Positive comments (all categories).

**Table S1. (Supplemental)** Proposed framework for the safer adoption of a MacGyvered device. Adapted from Turner and colleagues.[7]

1. Define the problem and rule out the suitability of existing solutions
2. List benchmark safety indices for the device
3. Seek broader feedback from all stakeholders on the design's utility and potential pitfalls.
4. Perform laboratory-based and in situ simulations.
5. Introduce into low-risk clinical settings after local due process and patient consent.
6. Introduce into higher-risk clinical settings with a discrete group of trained 'super-users'.
7. Encourage an iterative cycle of feedback, review, re-design and improvement.
8. **Do not:** adopt, publish, endorse or disseminate via social media a MacGyvered device without data to support safety.

For peer review only


For peer review only - http://bmjopen.bmj.com/site/about/guidelines.xhtml

**Table S2. (Supplemental). Lab-based testing of the Bubble PAPR prior to clinical evaluation**

All of the bench tests detailed below were carried out by the Electrical and Biomedical Engineering (EMBE) team based at Wythenshawe Hospital (MFT), the University of Manchester Mechanical Aerospace and Civil Engineering team (UoM) or by INSPEC International, Salford, UK. A PAPR unit was supplied, and the Instructions for Use were followed by the independent tester, with a judgement made if they fulfilled particular requirements. Some requirements are supplemented by the qualitative or quantitative data collected in the questionnaires. Standards used were British Standard EN12941 (BS, 2008) and the European Regulations for Respiratory Protective Equipment EU2016/425 (ER, 2016).

| Relevant section of standard | Standard detail | Test location | Test detail | Results/notes | Pass/Fail |
|---|---|---|---|---|---|
| BS 6.1.1<br>ER 1.3.2 | Suitable resistance to wear and tear | MFT | PAPR units inspected after 1 week of continual use. Images taken before and after. | Opinion. Baseline inspection +/- photograph. Review after 1 week | Pass 14/3/21 |
| | | | | | |
| BS 6.1.4<br>ER 1.2.1.2 | No sharp edges | MFT | Visual and physical inspection Reports from staff evaluation | Opinion. Baseline inspection +/- photograph. Review after 1 week | Pass 14/3/21 |
| | | | | | |
| BS 6.3.2 | Fits a range of head sizes | MFT | Ten participants will undergo fit testing. These participants will have height, weight and head circumference measured as part of this standard process. | Fit test data shared with EBME team. All fit factors >500 as per BS EN 12941 standard. | Pass 25/2/21 |
| | | | | | |
| BS 6.3.3.1 | Does not distort vision | MFT | The optical area appears transparent. | Inspection by EBME team | Pass 25/2/21 |
| BS 6.3.3.2 | Permits appropriate field of view | MFT | Reports from staff evaluation | Review of results from initial staff evaluations | Pass 14/3/21 |


For peer review only - http://bmjopen.bmj.com/site/about/guidelines.xhtml

| | | | | | |
|---|---|---|---|---|---|
| ER2.3 | | | | | |
| Relevant section of standard | Standard detail | Test location | Test detail | Results/notes | Pass/Fail |
| BS 6.9 | Cannot reverse airflow | MFT | Normal use.<br>Simulate blocked filter and blocked air duct.<br>Flowmeter. | | Pass 14/3/21 |
| BS 6.9 | Battery safe – protection from short circuit | MFT | EBME check on battery packs (suitable for purpose / recommended packs) | As per manufacturer documentation. Will not be separately tested. | Pass 25/2/21 |
| | | | | | |
| ER2.12 | Appropriate markings | MFT | Yoke manufactured with section for appropriate sticker | | Pass 25/2/21 |
| | | | | | |
| ER3.10.1 | Appropriate training provided | MFT | Instructions for use provided.<br>Training videos provided. | Instructions for Use provided to EBME.<br>Training videos available. | Pass 25/2/21 |
| | | | | | |
| No specific clause | Cleanable | MFT | Specification of foam material for yoke details cleaning methods, durability and material fatigue. | Specification provided to EBME. | Pass 25/2/21 |
| BS 6.16 | Mass shall not exceed 5kg. A maximum of 1.5kg shall be carried on the head. | MFT | Weigh the assembled Bubble PAPR | Assembled Bubble PAPR Weight = 1.4kg | Pass 14/3/21 |

For peer review only


For peer review only - http://bmjopen.bmj.com/site/about/guidelines.xhtml

| Relevant section of standard | Standard detail | Test location | Test detail | Results/notes | Pass/Fail |
|---|---|---|---|---|---|
| BS 6.1.3 | Repeated cleaning and disinfection – does not deteriorate | MFT | PAPR units are inspected after 1 week of continual use. Images taken before and after.<br><br>Specification of foam material for yoke details cleaning methods, durability and material fatigue. | Spec sheet and review after 1 week of use. | Pass 14/3/21 |
| | | | | | |
| BS 6.4 | Ingress protection test for 10 test subjects – using either or both methods (bitter and/or particulometer). | MFT | 10 users will undergo fit testing with particulometer. | Fit test data shared with EBME team. All fit factors >500 as per BS EN 12941 standard. | Pass 25/2/21 |
| ER 3.10.2 | Appropriate protection to eye and skin irritants | MFT | | | |

For peer review only


For peer review only - http://bmjopen.bmj.com/site/about/guidelines.xhtml

| Relevant section of standard | Standard detail | Test location | Test detail | Results/notes | Pass/Fail |
|---|---|---|---|---|---|
| BS 6.2 (6.4) | Repeated after hood/yoke is conditioned at maximum specified temperature and humidity.<br><br>Complete unit is stored for 72 +/- 1 hours at the upper extreme of temperature and humidity specified by the manufacturer. Unit is allowed to return to ambient conditions for 4 hours, then stored for 72 +/- 1 hours at the lower extreme of temperature and humidity. | MFT | Unit is subjected to particulometer fit testing after appropriate temperature conditioning. | *Conditioning beyond use on the ICU should not be required for the MFT in-house evaluation*<br><br>All fit tests took place on the Acute ICU at Wythenshawe | Pass 25/2/21 |
| | | | | | |
| BS 6.5 | Positive pressure inside the hood remains below 5mbar | MFT | 1 user and 1 dummy test head setup. Pressure measurement inside hood during regular use. | Measured pressure below 5mBar | Pass 14/3/21 |
| BS 6.6.2 | Exceeds manufacturer's minimum specified airflow for a period of at least 4 hours.<br><br>(The UK/EU regs do not specify a minimal flow. US regulations do, but this is not immediately relevant) | MFT, INSPEC | Test head and flow meter.<br><br>*Note the flow meter arrangement is slightly complex as the measurement itself can interfere with flow.* | Breathe Safety report reviewed. | Pass 25/2/21 |


For peer review only - http://bmjopen.bmj.com/site/about/guidelines.xhtml

| Relevant section of standard | Standard detail | Test location | Test detail | Results/notes | Pass/Fail |
|---|---|---|---|---|---|
| BS 6.7 | Check function of minimum airflow indicator. | MFT, INSPEC | Apply different flow rates to the yoke measured by external flow meter and evaluate performance of the minimum airflow indicator in units. | Breathe Safety report reviewed. | Pass 25/2/21 |
| | | | | | |
| EN 143<br>BS 6.8 | Clogging of filter | MFT, INSPEC | Flow through filter and yoke tested after 4 hours of use with an external flow meter. | Breathe Safety report reviewed. | Pass 25/2/21 |
| | | | | | |
| 6.13 | The carbon dioxide content of the inhalation air (dead space inside the hood) shall not exceed an average of 1% by volume.<br><br>There is a specific test-rig setup for this. A physiological surrogate model should provide adequate assurance that the $CO_2$ content inside the hood is <1% during normal use. | MFT, INSPEC | Oxygen and carbon dioxide (gas analysis) inside the hood measured as partial pressures and/or percentages using MFT EBME equipment. | Breathe Safety report reviewed. | Pass 25/2/21 |

For peer review only


For peer review only - http://bmjopen.bmj.com/site/about/guidelines.xhtml

| Relevant section of standard | Standard detail | Test location | Test detail | Results/notes | Pass/Fail |
|---|---|---|---|---|---|
| BS 6.15 | Exhalation means (valve) maximum flowrate and safe operation:<br><br>Hood performs adequately during normal use. Specifically; exhalation means:<br>• functions and can be replaced (new hood)<br>• functions in orientations encountered during normal use<br>• is protected against dirt and mechanical damage | MFT | Test subject wears hood.<br><br>Eventually, this section is supplemented with reports from staff evaluation. | Review of initial feedback from users in sim setting | Pass 14/3/21 |
| BS 6.15 | Exhalation means (valve) maximum flowrate and safe operation:<br><br>Continuous flow rate of 300 +/- 15 L/min is applied for a period of 60+/- 6 secs. | MFT | Flow generator (ventilator) and flow meter.<br><br>Visual inspection of exhalation valve. | Opinion of EBME team. The flow generator works as described with the test flowmeter supplied. Exhalation Valve inspection pass. | Pass 14/3/21 |


For peer review only - http://bmjopen.bmj.com/site/about/guidelines.xhtml

**Table S3. (Supplemental). Pilot data. Q14 & Q17 are Likert Scale items (rated 1-7) and Q8-11 are Visual Analogue Scale items (rated 0-100).**

| | Current PPE | | | | | |
|---|---|---|---|---|---|---|
| Participant | Q14 Safe to wear | Q17 Comfort to wear | Q8 Speak clearly to colleagues | Q9 Be heard by colleagues | Q10 Speak clearly to patients | Q11 Be heard by patients |
| 1 | 3 | 3 | 10 | 20 | 0 | 0 |
| 2 | 4 | 2 | 15 | 20 | 10 | 30 |
| 3 | 4 | 4 | 20 | 20 | 30 | 30 |
| 4 | 5 | 2 | 25 | 25 | 10 | 10 |
| 5 | 5 | 4 | 33 | 40 | 25 | 25 |
| 6 | 6 | 3 | 30 | 25 | 30 | 30 |
| 7 | 7 | 2 | 20 | 30 | 25 | 25 |
| 8 | 4 | 3 | 45 | 50 | 20 | 30 |
| Mean | 4.8 | 2.9 | 24.8 | 28.8 | 18.8 | 22.5 |
| SD | 1.3 | 0.8 | 11.1 | 10.9 | 10.9 | 11.3 |

| | BUBBLE-PAPR | | | | | |
|---|---|---|---|---|---|---|
| Participant | Q14 Safe to wear | Q17 Comfort to wear | Q8 Speak clearly to colleagues | Q9 Be heard by colleagues | Q10 Speak clearly to patients | Q11 Be heard by patients |
| 1 | 4 | 5 | 75 | 75 | 80 | 80 |
| 2 | 7 | 5 | 85 | 80 | 60 | 70 |
| 3 | 5 | 6 | 90 | 90 | 90 | 90 |
| 4 | 6 | 6 | 95 | 95 | 95 | 85 |
| 5 | 6 | 5 | 75 | 70 | 85 | 90 |
| 6 | 6 | 5 | 65 | 70 | 55 | 66 |
| 7 | 7 | 5 | 70 | 70 | 65 | 65 |
| 8 | 7 | 6 | 90 | 95 | 90 | 90 |
| Mean | 6.0 | 5.4 | 80.6 | 80.6 | 77.5 | 79.5 |
| SD | 1.1 | 0.5 | 10.8 | 11.2 | 15.4 | 11.0 |


For peer review only - http://bmjopen.bmj.com/site/about/guidelines.xhtml

**Table S4. (Supplemental) Fit testing data from the first 10 participants.**

Test protocol HSE INDG 479. Pass level set at a fit factor of 500.

| Subject | Self-reported height | Self-reported weight | BMI | Normal Breathing 1 | Deep Breathing | Head Side to Side | Head Up and Down | Talking | Bending at the waist | Normal Breathing 2 | Overall Fit Factor |
|---|---|---|---|---|---|---|---|---|---|---|---|
| **1** | 1.86 | 74 | 21.4 | 79705 | 43647 | 125478 | 11125 | 107899 | 1339 | 76152 | **7757** |
| **2** | 1.95 | 75 | 19.7 | 53792 | 52343 | 59440 | 51673 | 52733 | 50433 | 45961 | **52075** |
| **3** | NR | NR | NR | 38867 | 36699 | 37097 | 41474 | 39500 | 36884 | 37465 | **38217** |
| **4** | 1.82 | 65 | 19.6 | 17745 | 6622 | 3149 | 5028 | 31996 | 30520 | 31326 | **8539** |
| **5** | 1.65 | 55 | 20.2 | 24945 | 25215 | 3885 | 8097 | 28877 | 29107 | 24393 | **12268** |
| **6** | 1.67 | 58 | 20.8 | 24617 | 25608 | 25581 | 25225 | 20107 | 1924 | 23517 | **9088** |
| **7** | 1.52 | 47 | 20.3 | 28747 | 30700 | 33203 | 15275 | 31671 | 8327 | 26041 | **19829** |
| **8** | 1.65 | 65 | 23.9 | 27282 | 31318 | 34900 | 9697 | 29093 | 3514 | 25770 | **12544** |
| **9** | 1.83 | 115 | 34.3 | 11182 | 1123 | 11028 | 24524 | 24692 | 2537 | 23704 | **4408** |
| **10** | 1.59 | 75 | 29.7 | 25760 | 3419 | 16125 | 2433 | 25523 | 1552 | 26154 | **4588** |
| | | | | | | | | | | | |
| **Mean values** | | | | **33264** | **25669** | **34989** | **19455** | **39209** | **16614** | **34048** | **16931** |



For peer review only - http://bmjopen.bmj.com/site/about/guidelines.xhtml

STROBE Statement—checklist of items that should be included in reports of observational studies

| | Item No | Recommendation | Page No |
|---|---|---|---|
| **Title and abstract** | 1 | (*a*) Indicate the study's design with a commonly used term in the title or the abstract | 1 |
| | | (*b*) Provide in the abstract an informative and balanced summary of what was done and what was found | 2 |
| **Introduction** | | | |
| Background/rationale | 2 | Explain the scientific background and rationale for the investigation being reported | 3 |
| Objectives | 3 | State specific objectives, including any prespecified hypotheses | 4 |
| **Methods** | | | |
| Study design | 4 | Present key elements of study design early in the paper | 5 |
| Setting | 5 | Describe the setting, locations, and relevant dates, including periods of recruitment, exposure, follow-up, and data collection | 5 |
| Participants | 6 | (*a*) *Cohort study*—Give the eligibility criteria, and the sources and methods of selection of participants. Describe methods of follow-up<br>*Case-control study*—Give the eligibility criteria, and the sources and methods of case ascertainment and control selection. Give the rationale for the choice of cases and controls<br>*Cross-sectional study*—Give the eligibility criteria, and the sources and methods of selection of participants | 6 |
| | | (*b*) *Cohort study*—For matched studies, give matching criteria and number of exposed and unexposed<br>*Case-control study*—For matched studies, give matching criteria and the number of controls per case | |
| Variables | 7 | Clearly define all outcomes, exposures, predictors, potential confounders, and effect modifiers. Give diagnostic criteria, if applicable | 6 and Supplemental Material |
| Data sources/ measurement | 8* | For each variable of interest, give sources of data and details of methods of assessment (measurement). Describe comparability of assessment methods if there is more than one group | 6 |
| Bias | 9 | Describe any efforts to address potential sources of bias | 5 |
| Study size | 10 | Explain how the study size was arrived at | 6 |
| Quantitative variables | 11 | Explain how quantitative variables were handled in the analyses. If applicable, describe which groupings were chosen and why | 6 |
| Statistical methods | 12 | (*a*) Describe all statistical methods, including those used to control for confounding | 6 |
| | | (*b*) Describe any methods used to examine subgroups and interactions | N/A |


For peer review only - http://bmjopen.bmj.com/site/about/guidelines.xhtml

| | | | |
|---|---|---|---|
| | | (*c*) Explain how missing data were addressed | Intention to treat. No missing data. |
| | | (*d*) *Cohort study*—If applicable, explain how loss to follow-up was addressed<br>*Case-control study*—If applicable, explain how matching of cases and controls was addressed<br>*Cross-sectional study*—If applicable, describe analytical methods taking account of sampling strategy | |
| | | (*e*) Describe any sensitivity analyses | N/A |
| **Results** | | | |
| Participants | 13* | (a) Report numbers of individuals at each stage of study—eg numbers potentially eligible, examined for eligibility, confirmed eligible, included in the study, completing follow-up, and analysed | 7 |
| | | (b) Give reasons for non-participation at each stage | 7 |
| | | (c) Consider use of a flow diagram | Not required |
| Descriptive data | 14* | (a) Give characteristics of study participants (eg demographic, clinical, social) and information on exposures and potential confounders | 7 |
| | | (b) Indicate number of participants with missing data for each variable of interest | N/A |
| | | (c) *Cohort study*—Summarise follow-up time (eg, average and total amount) | 7 |
| Outcome data | 15* | *Cohort study*—Report numbers of outcome events or summary measures over time | 7 |
| | | *Case-control study*—Report numbers in each exposure category, or summary measures of exposure | |
| | | *Cross-sectional study*—Report numbers of outcome events or summary measures | |
| Main results | 16 | (*a*) Give unadjusted estimates and, if applicable, confounder-adjusted estimates and their precision (eg, 95% confidence interval). Make clear which confounders were adjusted for and why they were included | 7 |
| | | (*b*) Report category boundaries when continuous variables were categorized | 7 |
| | | (*c*) If relevant, consider translating estimates of relative risk into absolute risk for a meaningful time period | 7 |
| Other analyses | 17 | Report other analyses done—eg analyses of subgroups and interactions, and sensitivity analyses | 8 |
| **Discussion** | | | |
| Key results | 18 | Summarise key results with reference to study objectives | 9 |
| Limitations | 19 | Discuss limitations of the study, taking into account sources of potential bias or imprecision. Discuss both direction and magnitude of any potential bias | 9 |
| Interpretation | 20 | Give a cautious overall interpretation of results considering objectives, limitations, multiplicity of analyses, results from similar studies, and other relevant evidence | 9 |
| Generalisability | 21 | Discuss the generalisability (external validity) of the study results | 10,11 |
| **Other information** | | | |
| Funding | 22 | Give the source of funding and the role of the funders for the present study and, if applicable, for the original study on which the present article is based | 12 |

For peer review only


For peer review only - http://bmjopen.bmj.com/site/about/guidelines.xhtml

BMJ Open

# Bubble-PAPR: Phase I clinical evaluation of the comfort and perception of a prototype powered air-purifying respirator for use by healthcare workers in an acute hospital setting.

| | |
|---|---|
| Journal: | *BMJ Open* |
| Manuscript ID | bmjopen-2022-066524.R1 |
| Article Type: | Original research |
| Date Submitted by the Author: | 09-Dec-2022 |
| Complete List of Authors: | McGrath, Brendan; Manchester University NHS Foundation Trust; Manchester Academic Health Science Centre, Immunity and Respiratory Medicine<br>Shelton, Clifford; NHS Manchester; Lancaster University Lancaster Medical School<br>Gardner, Angela; Manchester University NHS Foundation Trust<br>Coleman, Ruth; Manchester University NHS Foundation Trust<br>Lynch, James; Manchester University NHS Foundation Trust<br>Alexander, Peter G; Manchester University NHS Foundation Trust; Manchester Academic Health Science Centre, Immunity and Respiratory Medicine<br>Cooper, Glen; The University of Manchester School of Mechanical Aerospace and Civil Engineering |
| <b>Primary Subject Heading</b>: | Intensive care |
| Secondary Subject Heading: | Anaesthesia, Communication, Infectious diseases, Intensive care, Nursing |
| Keywords: | OCCUPATIONAL & INDUSTRIAL MEDICINE, INTENSIVE & CRITICAL CARE, Infection control < INFECTIOUS DISEASES, ANAESTHETICS, RESPIRATORY MEDICINE (see Thoracic Medicine) |
| | |

SCHOLARONE™
Manuscripts

*I, the Submitting Author has the right to grant and does grant on behalf of all authors of the Work (as defined in the below author licence), an exclusive licence and/or a non-exclusive licence for contributions from authors who are: i) UK Crown employees; ii) where BMJ has agreed a CC-BY licence shall apply, and/or iii) in accordance with the terms applicable for US Federal Government officers or employees acting as part of their official duties; on a worldwide, perpetual, irrevocable, royalty-free basis to BMJ Publishing Group Ltd ("BMJ") its licensees and where the relevant Journal is co-owned by BMJ to the co-owners of the Journal, to publish the Work in this journal and any other BMJ products and to exploit all rights, as set out in our licence.*

*The Submitting Author accepts and understands that any supply made under these terms is made by BMJ to the Submitting Author unless you are acting as an employee on behalf of your employer or a postgraduate student of an affiliated institution which is paying any applicable article publishing charge ("APC") for Open Access articles. Where the Submitting Author wishes to make the Work available on an Open Access basis (and intends to pay the relevant APC), the terms of reuse of such Open Access shall be governed by a Creative Commons licence – details of these licences and which Creative Commons licence will apply to this Work are set out in our licence referred to above.*

*Other than as permitted in any relevant BMJ Author's Self Archiving Policies, I confirm this Work has not been accepted for publication elsewhere, is not being considered for publication elsewhere and does not duplicate material already published. I confirm all authors consent to publication of this Work and authorise the granting of this licence.*

For peer review only

**Title:**
Bubble-PAPR: Phase I clinical evaluation of the comfort and perception of a prototype powered air-purifying respirator for use by healthcare workers in an acute hospital setting.
**REVISION**

**Short title:**
Bubble-PAPR: a phase 1 study

**Authors**
Brendan A McGrath, Consultant & Honorary Professor[1,2]
Cliff Shelton, Consultant Clinical Senior Lecturer[1,3]
Angela Gardner, Clinical Fellow[1]
Ruth Coleman, Advanced Critical Care Practitioner[1]
James Lynch, Advanced Critical Care Practitioner[1]
Peter G Alexander, Consultant & Honorary Senior Lecturer[1,2]
Glen Cooper, Reader

1. Acute Intensive Care Unit, Wythenshawe Hospital, Manchester University NHS Foundation Trust
2. Manchester Academic Critical Care, Division of Infection, Immunity and Respiratory Medicine, School of Biological Sciences, Faculty of Biology, Medicine and Health, the University of Manchester, Manchester Academic Health Science Centre.
3. Lancaster Medical School, Lancaster University
4. Department of Mechanical, Aerospace & Civil Engineering, the University of Manchester

**Corresponding author**
Brendan A McGrath (CI)
Acute Intensive Care Unit
Wythenshawe Hospital
Southmoor Road
Wythenshawe
Manchester. M23 9LT
0161 291 6420
Brendan.mcgrath@manchester.ac.uk

For peer review only


For peer review only - http://bmjopen.bmj.com/site/about/guidelines.xhtml

**Structured abstract**

**Objectives**: We aimed to design and produce a low-cost, ergonomic, hood-integrated Powered Air-Purifying Respirator (Bubble-PAPR) for pandemic healthcare use, offering optimal and equitable protection to all staff. We hypothesised that participants would rate Bubble-PAPR more highly than current FFP3 face mask respiratory protective equipment (RPE).

**Design**: Rapid design and evaluation cycles occurred based on the identified user needs. We conducted diary card and focus group exercises to identify relevant tasks requiring RPE. Lab-based safety standards established against British Standard BS-EN-12941 and EU2016/425 covering materials; inward particulate leakage; breathing resistance; clean air filtration and supply; carbon dioxide elimination; exhalation means; and electrical safety. Questionnaire-based usability data from participating frontline healthcare staff before (usual RPE) and after using Bubble-PAPR.

**Setting**: Overseen by a trial safety committee, evaluation progressed sequentially through laboratory, simulated, low-risk, then high-risk clinical environments of a single tertiary NHS hospital.

**Participants:** 15 staff completed diary cards and focus groups. 91 staff from a range of clinical and non-clinical roles completed the study, wearing Bubble-PAPR for a median of 45 minutes (IQR 30-80 [15-120]). Participants self-reported a range of heights (mean 1.7m [SD 0.1, range 1.5-2.0]), weights (72.4kg [16.0, 47-127]) and body mass indices (25.3 [4.7,16.7-42.9]).

**Outcome measures**: Pre-use particulometer "fit testing" and evaluation against standards by independent biomedical engineer. Primary: perceived comfort (Likert scale). Secondary: perceived safety, communication.

**Results**: Mean fit factor 16,961 (ten participants). Bubble-PAPR mean comfort score 5.64(SD 1.55) versus usual FFP3 2.96(1.44) (mean difference 2.68 (95% CI 2.23-3.14, p<0.001). There was a significant difference in favour of Bubble-PAPR across all secondary outcomes.

**Conclusions**: Bubble-PAPR achieved its primary purpose of keeping staff safe from airborne particulate material whilst improving comfort and the user experience. The design and development of Bubble-PAPR were conducted using a careful evaluation strategy addressing key regulatory and safety steps, in contrast to many devices rapidly developed and deployed during the pandemic.

**Trial registration**: IRAS ID:288493, REC Ref:21/WA/0018. ClinicalTrials.gov (NCT04681365).

For peer review only



For peer review only - http://bmjopen.bmj.com/site/about/guidelines.xhtml

**Strengths and limitations of this study**

- We employed user-centred design, engineering optimisation and staged feasibility testing to develop a novel Powered Air-Purifying Respirator (Bubble-PAPR) for use specifically in frontline healthcare settings.
- The design of Bubble-PAPR met regulatory standards and our evaluation demonstrated that it met the key requirements of comfort and perceived safety identified as essential requirements by healthcare staff.
- The design and development of Bubble-PAPR were conducted using a careful strategy addressing key regulatory and safety steps, measured against UK/European standards, in contrast to many devices rapidly developed and deployed during the pandemic.
- A limitation of our study was the design and evaluation were undertaken at a single (large) hospital, using similar staff groups (but different staff).
- Bubble-PAPR is an excellent example of developing a cosmopolitan network that could become a key feature of future system resilience.

For peer review only



For peer review only - http://bmjopen.bmj.com/site/about/guidelines.xhtml

## Introduction

The COVID-19 global pandemic created a worldwide shortage of personal protective equipment (PPE)[1] and highlighted significant usability issues in current PPE products.[2] In addition to direct contact, airborne diseases may be spread by aerosol or droplet transmission. Aerosol transmission may be mitigated by the appropriate use of respiratory protective equipment (RPE), a particular classification of personal protective equipment (PPE). However, respiratory protective equipment is used as part of a hierarchy of control measures and is usually considered a last resort. This is because RPE only protects individual workers, is prone to failure or misuse (wrong RPE for the wrong task/environment) and wearers may get a false sense of security, encouraging risk-taking behaviours.[3] A range of inspiratory filtering devices exist: dust masks, half-face masks, full-face masks and powered (fan-assisted) respirators. Powered respirators include: half/full-face masks, helmets, hoods and visors. Though not used in healthcare, for completeness, breathing apparatuses are systems that supply an independent, positive pressure supply of breathing-quality air.

Face masks may be classified by considering the level of protection they offer the wearer to inhalation of environmental contaminants. Simple surgical face masks or 'nuisance' dust masks do not entirely filter droplets or aerosols. Filtering face piece (FFP) masks comprise layers of synthetic non-woven material with interleaved filtration layers and provide protection against small airborne particles (aerosols). Different types and constructions of FFP masks can be classified by their ability to filter small particles. Particulate filters can be classified as low (P1) to high (P3) efficiency, filtering between 80% of particles smaller than 2 micrometres to 99.95% of particles smaller than 0.5 micrometres, respectively (Table 1).[4] Respiratory protection can therefore be considered in terms of a combination of the filtering ability of the device relative to the exposure environment and its fit on the wearer's face. A device is considered adequate if it has the capacity to reduce the wearer's exposure to a hazardous substance to acceptable levels (to comply with occupational exposure limit values). Devices can be reusable, but the majority are single-use. Masks are difficult to recycle due to their layered construction and the pandemic contributed to an unprecedented rise in RPE-related clinical waste.[5]

The majority of RPE used in healthcare settings are disposable face masks adopted from industry. Masks are not designed to be worn for long periods or repeated shifts, may restrict vision and communication, may cause facial damage due to their tight fit, and require multiple time-consuming 'fit tests' for each model of the device for each staff member. All these issues were highlighted in the context of the 2002-2004 SARS epidemic.[6] More appropriate solutions for prolonged and repeated use include powered air-purifying respirators (PAPRs). But, again, these are not designed primarily for healthcare, are heavy, noisy, expensive, difficult to clean to clinical standards, and not suitable for the specific needs in frontline healthcare environments.

There have been several widely reported 'homemade' or 'MacGyvered' devices that well-intentioned groups or individuals developed to protect staff and patients during the pandemic.[7] In a time of crisis, these innovations were often rapidly developed without significant funding and delivered to areas of need during a time of global RPE shortage. However, due to the urgency of the situation, none of these devices sought or achieved independent certification or provided data to support safety.[8] Turner and colleagues proposed a framework for the safer adoption of novel devices[7] which: defines the problem and reviews existing solutions, benchmarks safety indices for the devices, and then evaluates it in a structured manner through simulated, low- and then high-risk clinical settings Table S1



For peer review only - http://bmjopen.bmj.com/site/about/guidelines.xhtml

(Supplemental). Broad stakeholder feedback is encouraged through iterative review cycles, re-design and improvements.

**Table 1.** Classification of particulate filters, with a worked example and fit testing. Data from EU Standard 149:2001 Respiratory Protective Devices.

**P1** – Filters about 80% of particles smaller than 2 micrometres
**P2** – Filters about 94% of particles smaller than 0.5 micrometres
**P3** – Filters about 99.95% of particles smaller than 0.5 micrometres

A respiratory protective device is considered adequate if it has the capacity to reduce the wearer's exposure to a hazardous substance to acceptable levels. The ratio of airborne particles outside:inside the filtering device gives a **nominal (theoretical) protection factor**. An **assigned protection factor** reflects the actual workplace conditions. For example: an airborne dust contaminant with an occupational exposure limit of 5mg/m$^3$ may be present in the workplace in concentrations up to 60mg/m$^3$ (determined by monitoring). A particle filter is needed to reduce the concentration by at least a factor of 12 (60/5=12). A P3 filter with an assigned protection factor of 20 would be suitable (as this is greater than the factor of 12 required). Other considerations such as exposure time, useability and disposal of the device need to be considered prior to undertaking a **fit test** with the intended wearer.

A fit test verifies that a **specific model** of device works as intended with a **particular individual**. For example, different face shapes and facial hair can interfere with a particular system's ability to filter environmental contaminants effectively.

**Qualitative fit testing** assesses the inward leakage past a mask of airborne compounds detectable by the wearer (typically bitter/sweet tasting substances), aerosolised using a spray device.

**Quantitative fit testing** measures particulate concentrations inside and outside of devices, typically undertaken by measuring sodium chloride aerosolised in water to generate a 'particle' count. Quantitative fit testing generates a **fit factor** – the ratio of airborne particle counts outside:inside. The fit factor takes account of the whole device (the filter, hood and airflow in the case of a PAPR). Fit factors for PAPRs are very high (optimal protection) and so if correctly worn, fit testing prior to use is not usually required.

For peer review only

Considering the above, our project aimed to design and produce a low-cost, ergonomic, hood-integrated PAPR for use in frontline healthcare settings. Our objectives were to focus on user-centred design, engineering optimisation, staged feasibility testing, certification, intellectual property protection and then rapid manufacture and distribution. We also aimed to design the PAPR to be re-used, refurbished and recycled where possible, using readily available, simple and interchangeable key parts which proved difficult to source during the early stages of the pandemic. Finally, by designing an available, affordable PAPR system that could be cleaned appropriately and re-used between different staff, we aimed to provide equitable access to high-quality RPE that offered optimal protection to *all* staff, wherever they worked.[9] We hypothesised that participants would rate Bubble-PAPR more highly than current FFP3 face mask RPE across the domains of comfort, perceived safety and communication.


For peer review only - http://bmjopen.bmj.com/site/about/guidelines.xhtml

**Methods**

The design team brought together frontline clinical staff based in the Wythenshawe Hospital Acute Intensive Care Unit (ICU) of Manchester University NHS Foundation Trust (MFT), an experienced product design consultancy (Designing Science Limited, Middlesex, UK) and the technical expertise of the School of Engineering at the University of Manchester (UoM). Research Ethical and Health Research Authority approval (IRAS ID:288493, REC Ref:21/WA/0018) was granted. The study was sponsored by MFT, who acted as the manufacturer of this in-house prototype device, which became known as Bubble-PAPR. The study protocol, analysis plan and recruitment metrics were registered and reported at ClinicalTrials.gov (NCT04681365). Participating staff were provided with participant information sheets, a detailed explanation and demonstration of the safe use of Bubble-PAPR, and written consent was obtained. User needs assessment was conducted through a series of workplace diary card exercises documenting typical activities undertaken by frontline healthcare staff, synthesised in focus groups. Staff were invited to participate (by email and posters in rest areas) from clinical locations where RPE was mandated within the hospital. The first two respondents from each area were recruited to the diary card and focus group activities. Rapid design and evaluation cycles occurred based on the identified user needs. In addition, evaluation of early prototypes occurred in simulated clinical environments, collecting usability data from participants.

Patient and public involvement

Public and Patient involvement was undertaken through the Manchester Academic Critical Care research group's patient forum. There were powerful accounts from patients who regularly described not being able to understand what hospital staff wearing PPE were saying and being troubled that they had no idea what their carers looked like. These reports led us to focus on prioritising the ease of communication with Bubble-PAPR. Staff participants who were invited to wear Bubble-PAPR were recruited from clinical locations where RPE was mandated, by direct invitation from the research team.

A Trial Safety Committee was established to oversee the results of laboratory and bench testing of the prototype, initial safety data, usability, and adverse event data at each stage of the evaluation. The Committee met prior to commencing clinical evaluation. It was tasked with the decision to allow the evaluation to proceed between phases: simulated clinical environment, low-risk (non-infectious) clinical environment and high-risk clinical environment (COVID-19 wards and ICUs). Early iterations of Bubble-PAPR included 3-D-printed collars and key parts (such as the impellor), along with a variety of designs of the hood. A final iteration of Bubble-PAPR included a medical-grade foam collar, precision-machined internal components and a revised (smaller) hood was further tested in high-risk environments. Prior to first use, several device safety checks were independently undertaken by the MFT Electrical and Biomedical Engineering Department and INSPEC International Ltd, Salford, UK). A short report addressing the qualitative and qualitative criteria detailed in the relevant standards, and summarised in Table S2, S3 and S5 (Supplemental), was presented to the Trial Safety Committee. The first ten study participants to wear Bubble-PAPR underwent ‘fit testing’ with a particulometer (TSI Portacount Fit Tester 8040, TSI Instruments Ltd, Buckinghamshire, UK) following a standard protocol derived from the UK Government’s Health and Safety Executive.[10] Fit testing is not required before wearing PAPRs, including Bubble-PAPR. The purpose of fit testing was to collect device performance data and to allow the research team to assure the Trial Safety Committee that Bubble-PAPR was



For peer review only - http://bmjopen.bmj.com/site/about/guidelines.xhtml

performing to an appropriate standard. This INDG-479 protocol requires a ‘Fit Factor’ pass level of 100 for FFP3/N95 face masks and 500 for full face masks/hoods. Participants followed this standard protocol during quantitative fit testing which involved the following exercises undertaken for at least 60 seconds: normal breathing; deep breathing; turning head from side-to-side; moving head up and down; talking; bending over to 90 degrees; repeat normal breathing. European Conformity Standard EN12941 requires an applied fit factor of 40 for a ‘loose-fitting hood’ PAPR; the equivalent of a nominal protection factor of at least 500 (accepting an inward leakage of 0.2% with a P3 class filter. See Table 1). By comparison, the minimal fit factor for an FFP3 mask in a clinical environment is 100. Tests were conducted in an ICU side room with a particle generator to reach background counts between 70,000 to 100,000 particles/$cm^3$.

The primary outcome was based on Davis’ technology acceptance model (perceived usefulness and perceived ease-of-use overcoming barriers to adoption)[11]. First, staff were asked to rate their experiences using current RPE (a variety of re-useable or disposable FFP3 masks) using a series of questions based on Likert-type scales (see Supplemental material). Next, safe use of the Bubble-PAPR was explained, and instructions for use were provided, supported by videos of donning, doffing, cleaning and storage. Bubble-PAPR was then worn during simulated/clinical use where the usual tasks (identified in the focus groups) were undertaken. Finally, after removal (doffing) of Bubble-PAPR, staff were immediately invited to complete a second questionnaire focused on the prototype. Free text comments were also invited.

The primary endpoint was staff rating of the comfort of Bubble-PAPR (versus current FFP3 face masks). Secondary endpoints focused on communication and perceived safety. Specifically, this was staff ratings of the prototype in terms of: how safe participants felt, ease of communication with colleagues, and ease of communication with patients (again, Bubble-PAPR versus current FFP3 face masks). Additional questions explored wearer anxiety, ease of use, and performance whilst undertaking usual work tasks. In parallel, in-house device feasibility testing was conducted in the hospital environment to test ergonomics and air particle filtration. The research framework for this study was based around in-house exemption for device development from the UK Medicines and Healthcare products Regulatory Agency (MHRA). This means that the hospital, acting as manufacturer, can use a device it has developed itself internally. Such a device is not required to undergo to independent testing and therefore it will not achieve a certificate of conformity (UK-Conformity Assessed or Conformitè Europëenne marking). However, in order to assure the study Sponsor and staff participants of the safety and efficacy of Bubble-PAPR, we tested against existing conformity standards for PAPRs relevant at the time of development (British Standard BS EN 12941 [Respiratory Protective Devices: Powered filtering devices incorporating a helmet or hood] and the European Union Personal Protective Equipment Directive EU2016/425).[4 12] Some of the testing was undertaken internally by independent biomedical engineers, with the flowrate and carbon dioxide testing undertaken externally.

A pilot evaluation was conducted in August 2020 to test the questionnaires and to assess the likely population means for the test scores (Table S3 Supplemental). We calculated a sample size of 20 participants would be required for each phase of the evaluation to detect a significant difference between usual PPE and Bubble-PAPR, based on a mean difference of 2.5 (SD 0.9) points on the 7-point Likert scale identified during the pilot evaluation (alpha = 0.05, 90% power). In addition, we allowed


For peer review only - http://bmjopen.bmj.com/site/about/guidelines.xhtml

for a 5% dropout and missing data rate, concluding 22 participants per phase. All variables were explored via appropriate graphical and descriptive statistics to evaluate distributions, data completeness and form. Analyses were conducted in RStudio 2020 (Boston, MA, www.rstudio.com). Analyses were performed separately for each phase for presentation to the Trial Safety Committee, with a pooled analysis conducted at the study conclusion. Comparisons between groups (current RPE vs Bubble-PAPR) were made using a paired t-test or Wilcoxon signed-rank test as appropriate.

For peer review only

**Results**

The final design of Bubble-PAPR is shown schematically in Figure 1 (www.bubble-papr.com). The device safety checks and fit testing results are presented in Tables S2, S3 and S4 (Supplemental), respectively, demonstrating a mean fit factor of 16,961. Additional particulometer tests were undertaken with deliberate tears up to 20 cm in the hood using a dummy head. The lowest fit factor recorded with the damaged hood was 1,123. Therefore, the Trial Safety Committee concluded that the Bubble-PAPR performed its primary purpose of adequately protecting staff from airborne environmental contaminants.

Fifteen staff contributed to the diary and focus group exercises, generating a list of tasks to be undertaken. One staff member from the 16 invited could not attend the focus group meeting. Staff reported a range of patient-facing activities, including: verbal communication between colleagues and patients; writing; typing; reading notes, computer screens and monitors; manual handling; invasive procedures; emergency resuscitation; airway management; and maintenance of a clean/safe bedside environment. Over the course of the evaluation, staff completed all of the tasks identified by the diary exercise whilst wearing Bubble-PAPR in the clinical environment. Ninety-one staff wore Bubble-PAPR for a median of 45 (IQR 30-90, range 10-150) minutes between 3rd March and 21st December 2021. All relevant staff working in relevant clinical areas were approached until a maximum of six staff had been recruited per shift (the most that the research team could reasonably accommodate per shift), or the recruitment target had been met. No staff who were approached during their clinical shifts were unwilling or unable to trial Bubble-PAPR. There were no Bubble-PAPR-related safety incidents reported during the study. Staff undertook all clinical duties identified by the focus groups and diary card exercise, either in the simulation suite (n=22) or clinical settings (n=22 low-risk, n=25 high-risk, n=22 high-risk with final iteration). Participants predominantly declared as female (69%), and were from a range of clinical and non-clinical roles, Figure S1 (Supplemental). Staff self-reported a range of heights (mean 1.7m [SD 0.1, range 1.5-2.0]), weights (72.4kg [16.0, 47-127]) and body mass indices (25.3 [4.7,16.7-42.9]), Figure S2 (Supplemental). Fifty-two percent of participants reported that they normally wore glasses, with 31% wearing glasses during the evaluation. All participants described at least 6 month's experience with FFP3 face masks on a regular basis ("most shifts"), with a combination of re-useable (typically 3M™ 6000 Series Respirators) and single use (typically 3M™ Aura™ 9330 or equivalent) face masks. No participants described using PAPRs in the six months prior to recruitment. All participants completed all mandatory questionnaire sections.

With pooled data for the primary outcome, "*How comfortable do you feel in your PPE?"* (Likert scale bounded by 1 [very uncomfortable] to 7 [very comfortable]), Bubble-PAPR mean score was 5.64 (SD 1.55) versus usual FFP3 face mask 2.96 (1.44), Figure 2. There was a mean difference of 2.68 (95% CI 2.23-3.14, p<0.001). Secondary outcomes focused on communication and perceived safety. For the question, "*How safe do you feel in your PPE?*", Bubble-PAPR mean score was 6.15 (0.94) vs usual FFP3 face mask 5.43 (0.98); mean difference 0.73 (95% CI 0.45-1.00, p<0.001), Figure 2. Figure 3 demonstrates communication outcomes for all 91 comparisons of Bubble-PAPR versus usual FFP3 face masks. All adjusted comparisons were significant (p<0.001) in favour of Bubble-PAPR for communicating with both colleagues and patients (Table 2).


For peer review only - http://bmjopen.bmj.com/site/about/guidelines.xhtml

Secondary outcomes where a lower Likert response was considered better are presented in Figure S3 (Supplemental). These focussed on whether staff were worried about themselves or others whilst wearing RPE, whether the devices caused pressure or pain or if communication was impaired. Finally, staff were asked if they had to cut short a clinical (or simulated) encounter due to discomfort with their RPE. Again, there was a significant difference in favour of Bubble-PAPR for all metrics (all $p<0.001$, Table 2).

For peer review only


For peer review only - http://bmjopen.bmj.com/site/about/guidelines.xhtml

**Table 2.** Rating scales, summary results and comparisons across the questionnaire domains.

| | PPE | Q1 Confidence in donning | Q2 Confidence in donning without dislodging other PPE | Q3 Wear with glasses/goggles | Q4 Protect yourself from respiratory infection | Q5 Protect patient from infection from you | Q6 Safely care for your patient | Q7 Safely roll patient | Q8 Speak to staff | Q9 Be heard by staff | Q10 Speak to patient | Q11 Be heard by patient | Q12 Doff safely | Q13 Doff without dislodging glasses |
|---|---|---|---|---|---|---|---|---|---|---|---|---|---|---|
| Rating scale | From: | 0 - no confidence | 0 - no confidence | 0 - no confidence | 0 - no confidence | 0 - no confidence | 0 - no confidence | 0 - no confidence | 0 - no confidence | 0 - no confidence | 0 - no confidence | 0 - no confidence | 0 - no confidence | 0 - no confidence |
| | To: | 10 - fully confident | 10 - fully confident | 10 - fully confident | 10 - fully confident | 10 - fully confident | 10 - fully confident | 10 - fully confident | 10 - fully confident | 10 - fully confident | 10 - fully confident | 10 - fully confident | 10 - fully confident | 10 - fully confident |
| | | | | | | | | | | | | | | |
| RPE type | FFP3 | 8.9 (1.4) [3 - 10] | 8.3 (2) [2 - 10] | 6.9 (2.6) [2 - 10] | 8.2 (1.6) [4 - 10] | 8.2 (1.7) [2 - 10] | 8.4 (1.4) [5 - 10] | 8.2 (1.8) [2 - 10] | 5.1 (2.4) [1 - 10] | 4.9 (2.3) [1 - 10] | 4.8 (2.4) [1 - 10] | 4.7 (2.5) [1 - 10] | 8.1 (1.9) [2 - 10] | 6.2 (2.5) [1 - 10] |
| | Bubble | 7.4 (1.8) [3 - 10] | 7.7 (1.8) [2 - 10] | 7.6 (1.9) [3 - 10] | 8.6 (1.6) [3 - 10] | 8.5 (1.8) [2 - 10] | 8.0 (2) [2 - 10] | 7.8 (2.2) [2 - 10] | 7.5 (2.4) [1 - 10] | 7.1 (2.3) [1 - 10] | 7.8 (2.1) [2 - 10] | 7.4 (2.4) [1 - 10] | 8.0 (1.8) [2 - 10] | 7.8 (1.9) [2 - 10] |
| Comparison | Mean difference | -1.48 | -0.55 | 0.7 | 0.43 | 0.3 | -0.42 | -0.42 | 2.38 | 2.16 | 2.99 | 2.7 | -0.1 | 1.66 |
| | 95% CI | -1.9 to -0.99 | -1.12 to 0.02 | -0.00 to 1.40 | -0.04 to 0.89 | -0.18 to 0.78 | -0.91 to 0.07 | -0.98 to 0.15 | 1.66 to 3.11 | 1.45 to 2.88 | 2.36 to 3.62 | 1.97 to 3.433 | -0.63 to 0.43 | 0.98 to 2.34 |
| | | Favours FFP3 | No difference | Favours Bubble | No difference | No difference | No difference | No difference | Favours Bubble | Favours Bubble | Favours Bubble | Favours Bubble | No difference | Favours Bubble |
| | Adjusted p | <0.001 | 0.058 | 0.049 | 0.070 | 0.217 | 0.092 | 0.144 | <0.001 | <0.001 | <0.001 | <0.001 | 0.711 | <0.001 |

| | PPE | Q14 How safe does it feel | Q15 Worried about own health | Q16 Worried others health | Q17 Comfortable | Q18 Don ease | Q19 Doff ease | Q20a Restricted communication | Q20b Vision distorted with Bubble | Q20c Read monitors, computers, and notes with Bubble | Q21 Pressure marks on head/face | Q22 Pain on head/face | Q23 Leave clinical area early due to RPE |
|---|---|---|---|---|---|---|---|---|---|---|---|---|---|
| Rating scale | From: | 1 - very unsafe | 1 - not worried at all | 1 - not worried at all | 1 - very uncomfortable | 1 - not at all easy | 1 - not at all easy | 1 - not at all restricted | 1 - not at all affected | 1 - clear at all times | 1 - never | 1 - never | 1 - never |
| | To: | 7 very safe | 7 - very worried | 7 - very worried | 7 - very comfortable | 7 - very easy | 7 - very easy | 7 - very restricted | 7 - very affected | 7 - not clear at all | 7 - always | 7 - always | 7 - always |
| | | | | | | | | | | | | | |
| RPE type | FFP3 | 5.4 (1) [3 - 7] | 3.2 (1.5) [1 - 7] | 3.2 (1.5) [1 - 7] | 3 (1.4) [1 - 6] | 4.9 (1.3) [2 - 7] | 5.1 (1.3) [2 - 7] | 5.4 (1.4) [1 - 7] | - | - | 5.8 (1.4) [1 - 7] | 5.3 (1.4) [1 - 7] | 3.6 (1.6) [1 - 7] |
| | Bubble | 6.2 (0.9) [3 - 7] | 2.3 (1.6) [1 - 7] | 2.3 (1.7) [1 - 7] | 5.6 (1.6) [1 - 7] | 5.5 (1.4) [2 - 7] | 5.7 (1.2) [2 - 7] | 3.9 (1.7) [1 - 7] | 3.2 (1.9) [1 - 7] | 5.6 (1.5) [2 - 7] | 1.3 (0.8) [1 - 6] | 1.4 (0.9) [1 - 6] | 1.5 (1) [1 - 6] |
| Comparison | Mean difference | 0.73 | -0.92 | -0.93 | 2.68 | 0.62 | 0.63 | -1.49 | - | - | -4.54 | -3.99 | -2.13 |
| | 95% CI | 0.45 to 0.99 | -1.36 to -0.49 | -1.36 to -0.48 | 2.23 to 3.14 | 0.21 to 1.02 | 0.26 to 0.99 | -1.95 to -1.04 | - | - | -4.90 to -4.17 | -4.35 to -3.63 | -2.51 to - 1.75 |
| | | Favours Bubble | Favours Bubble | Favours Bubble | Favours Bubble | Favours Bubble | Favours Bubble | Favours Bubble | No comparator | No comparator | Favours Bubble | Favours Bubble | Favours Bubble |
| | Adjusted p | <0.001 | <0.001 | <0.001 | <0.001 | 0.003 | 0.002 | <0.001 | - | - | <0.001 | <0.001 | <0.001 |

For peer review only

During the initial phases, there was no significant difference between staff reporting ease of donning and doffing of Bubble-PAPR and usual PPE (which staff had used for many months at the time of the evaluation). However, pooled results saw staff becoming more familiar with the Bubble, and Bubble-PAPR was rated easier to don and doff when compared with usual FFP3 face masks (adjusted p=0.003 and 0.002 respectively), Table 2 and Figure S4 (Supplemental). One hundred and thirty-two additional free text comments were reviewed and categorised into positive (n=47, 35.6%), negative (67, 50.8%) and neutral (18, 13.6%) comments (Figures S5-7 Supplemental). Most comments focused on the noise of the device, which improved throughout the project as the impellor and motor were made quieter in later design iterations. The categories and nature of comments were as follows: Noise (33 comments [3 neutral, 30 negative]); Comfort (24 comments [20 positive, 2 neutral, 2 negative]); Communication (22 comments [5 positive, 6 neutral, 11 negative]); General (21 comments [17 positive, 2 neutral, 2 negative]); Vision (14 comments [1 positive, 4 neutral, 9 negative]); Wear and fit (10 comments (2 positive, 1 neutral, 7 negative); Stethoscope (5 negative comments); Safety (2 positive comments); Battery (1 negative comment).

For peer review only



For peer review only - http://bmjopen.bmj.com/site/about/guidelines.xhtml

**Discussion**

Our project developed an innovative prototype PAPR explicitly designed for prolonged healthcare use in high-risk clinical environments. Bubble-PAPR achieved its primary purpose of protecting staff from airborne potentially infectious material whilst also being rated significantly higher for comfort (the primary outcome), perceived safety, and communication with colleagues and patients (secondary outcomes) than usual RPE. Bubble-PAPR was used in all relevant simulated and clinical scenarios identified by detailed staff diary cards, making the results of this study extremely relevant to hospital-based healthcare workers.

Bubble-PAPR was rapidly developed based on the lived experiences of frontline staff during the early stages of the coronavirus pandemic, addressing the unmet needs of reliable, high-quality, universal and available RPE with improved comfort and communication when compared to usual FFP3 face masks. Staff overwhelmingly recognised the importance of facial visualisation when communicating with colleagues and patients. When combined with the improved comfort of wearing a PAPR over usual RPE, participants rated Bubble-PAPR consistently highly across all comparator domains.

This relatively simple evaluation study was preceded by a rapid design and prototyping phase, producing a working prototype within a few weeks. Despite the speed and agility demonstrated by the design team, we adhered to relevant conformity standards for PAPRs, following a tiered evaluation within the governance structure of an approved and regulated research project. Bubble-PAPR was only introduced into higher-risk environments following review by the Trial Safety Committee. This structured approach contrasted with some other rapidly developed or adopted pandemic RPE systems.[7, 13, 14] Whilst the PPE shortages experienced during the pandemic drove many of these innovations and adaptations, we recognised the importance of a methodical approach to design, development and testing of our prototype, both in the laboratory and clinical settings. We recommend others to follow the framework proposed by Duggan et al. for the development of novel medical devices, with regular reviews of safety and useability data within the framework of a robust and transparent clinical trial.[7]

Our study has some limitations. Some of the endpoints were self-reported by participating staff and not independently verified. This included communication between colleagues and between staff and patients. However, staff were performing their usual clinical duties whilst wearing Bubble-PAPR we are confident that any limitations of two-way communication would have been recognised and reported. The design of Bubble-PAPR addressed many of the issues identified by the same staff who subsequently evaluated the prototype. Whilst our study protocol allowed evaluation only within our Trust owing to the 'in-house' manufacturing exemption for testing, it is not unreasonable to expect similar results if our prototype were evaluated elsewhere. Although this may be considered a weakness of the study, many of the shortcomings of the PPE provided to frontline health workers around the world are well described and are essentially the same as those identified in our project.[15, 16] Furthermore, we evaluated Bubble-PAPR against single-use and reusable FFP3 face masks, which could be construed as comparing two different classes of RPE. However, Bubble-PAPR was designed and developed to provide a viable alternative to FFP3 class face masks, in contrast to the more usual healthcare use of PAPRs. Other PAPRs are more complex, more cumbersome (belt-worn fans and hoses), more costly, and typically are selectively available on a limited basis to specific users or groups


For peer review only - http://bmjopen.bmj.com/site/about/guidelines.xhtml

because of these factors. Although a pricing structure is currently unavailable, the simplicity of the design and components (designed with pandemic supply chain limitations in mind) means that Bubble-PAPR is likely to cost around 25-50% of the list price of equivalent PAPRs. Our detailed analysis of work diary cards from various clinical staff ensured that Bubble-PAPR was used for all relevant procedures undertaken by medical, nursing, healthcare assistant, allied healthcare professional (speech and language therapy, physiotherapy, pharmacy), administrative and domestic staff in the clinical area. Staff were able to undertake their usual duties with this simple, collar-worn PAPR. Although the design is simple, with no electronic indicators or alarms, this did not impact on conformity testing or function. This perspective, testing safety, performance and the user experience, is unique within published respiratory protective equipment product evaluation studies.[17, 18]

Our study did not directly evaluate the patient experience with staff wearing different RPE. However, the patient experience was reflected in the user specifications identified around communication, and anecdotal feedback was positive from patients, especially around facial visibility and verbal and non-verbal communication. In addition, when contrasted with FFP3 face masks, speech and language therapists reported that demonstrating speech and swallow exercises was suddenly possible with Bubble-PAPR and that the transparent nature of the hood overcame the communication barriers that can be so devastating for those with hearing impairments.[19] Although designed to be potentially recyclable, future work should address the environmental impact of PVC hoods with reusable collars compared to single-use or reusable FFP3 face masks.

For peer review only



For peer review only - http://bmjopen.bmj.com/site/about/guidelines.xhtml

**Conclusions**

Our study has demonstrated that Bubble-PAPR achieved its primary purpose of keeping staff safe from airborne particulate material whilst improving comfort, communication and the user experience when compared to usual RPE worn throughout the pandemic. It is likely that the patient experience was also enhanced. Bubble-PAPR has been patented (PCT/GB2021/052147) and subsequently licenced to a UK-based healthcare manufacturer for large-scale manufacture and distribution to frontline NHS and other workers. The pandemic drove unprecedented collaboration between clinicians, academics and industry. Bubble-PAPR is an excellent example of developing a cosmopolitan network that could become a key feature of future system resilience.

For peer review only


For peer review only - http://bmjopen.bmj.com/site/about/guidelines.xhtml

**Acknowledgements**

We are grateful to our funders (detailed below) for supporting this project and the staff who participated in the evaluation. In addition, we are indebted to the designers, engineers and staff who gave their time freely during the early stages of the coronavirus pandemic to work tirelessly on designing, building, testing and refining Bubble-PAPR. Specifically:

Patrick Hall, Designing Science Ltd (www.designingscience.co.uk)
Andrew Spragg, Industrial design consultant
Andrew Forbes, XK Design Ltd
James Corden, Manchester University Hospital NHS Foundation Trust Innovation Team
Nick Duggan, Innovation Consultant, Zuas (www.zuas.io)
GAMA Healthcare Ltd, Hemel Hempstead, UK (www.gamahealthcare.com)

**Conflict of interests**

Manchester University NHS Foundation Trust, the University of Manchester and Designing Science Ltd have agreed commercial terms to license Bubble-PAPR to GAMA Healthcare Ltd for manufacture and development, branded as RediHood™.

**Funding**

This project was supported by unrestricted grants and funding from:

- Engineering and Physical Sciences Research Council (EPSRC) Impact Acceleration Account 302
- Oxford Road Corridor (grant award number N/A)
- Health Innovation Manchester 'Momentum' special projects fund 2021 (grant award number N/A)
- Acute ICU Charitable Research Fund, Manchester University NHS Foundation Trust (grant award number N/A)
- Manchester University NHS Foundation Trust (grant award number N/A)

**Data availability statement**

Due to the commercial sensitivity of the intellectual property licensed at the conclusion of this project, the full dataset is not publicly available. However, the corresponding author will consider requests to disclose the dataset on an individual basis if necessary.

**Trial registration**

Research Ethical and Health Research Authority approval (IRAS ID:288493, REC Ref:21/WA/0018). Manchester University NHS Foundation Trust sponsored the study, acting as the manufacturer of this in-house prototype device. The study protocol, analysis plan and recruitment metrics were registered and reported at ClinicalTrials.gov (NCT04681365).



For peer review only - http://bmjopen.bmj.com/site/about/guidelines.xhtml

**Author contributions**

All authors critically revised the manuscript for important intellectual content and approved the final manuscript. BAM attests that all listed authors meet authorship criteria and that no others meeting the criteria have been omitted.

**BAM:** conception and design, collection, analysis and interpretation of data, drafting and revision of the manuscript, and final approval of the version to be published. Participant recruitment.

**CS:** qualitative work package conception and design, analysis and interpretation of data. Participant recruitment. Drafting and revision of the final manuscript, and final approval of the version to be published.

**AG:** qualitative work package design, analysis and interpretation of data. Drafting and revision of the final manuscript, and final approval of the version to be published.

**RC:** design, collection, and interpretation of data, drafting and revision of the manuscript, and final approval of the version to be published.

**JL:** design, collection, and interpretation of data, drafting and revision of the manuscript, and final approval of the version to be published.

**PGA:** design, collection, and interpretation of data, drafting and revision of the manuscript, and final approval of the version to be published.

**GC:** design, collection, and interpretation of data, drafting and revision of the manuscript, and final approval of the version to be published. Manufacturing and engineering lead.

**Provenance and peer review**
Not commissioned; externally peer reviewed.

**Word count of main document:** 3111

**Ethics approval**

Research Ethical and Health Research Authority approval (IRAS ID:288493, REC Ref:21/WA/0018) was granted from Wales REC5 on 27th January 2021. The study was sponsored by Manchester University NHS Foundation Trust, who acted as the manufacturer of this in-house prototype device. The study protocol, analysis plan and recruitment metrics were registered and reported at ClinicalTrials.gov (NCT04681365).

For peer review only



For peer review only - http://bmjopen.bmj.com/site/about/guidelines.xhtml

## Table and Figure legends

**Table 1.** Classification of particulate filters, with a worked example and fit testing.

**Table 2.** Rating scales, summary results and comparisons across the questionnaire domains.

**Table S1. (Supplemental)** Proposed framework for the safer adoption of a MacGyvered device. Adapted from Turner and colleagues.[7]

**Table S2. (Supplemental)** Lab-based testing of the Bubble PAPR prior to clinical evaluation.

**Table S3. (Supplemental).** Pilot data. Q14 & Q17 are Likert Scale items (rated 1-7) and Q8-11 are Visual Analogue Scale items (rated 0-100).

**Table S4. (Supplemental)** Fit testing data from the first 10 participants.

**Figure 1.** Bubble-PAPR comprises a medical-grade foam neck collar and a separate PVC hood. The universal fit collar draws air in through a filter via an impellor powered by an external battery. The collar has a mechanical low flow indicator and can be cleaned and reused by different users. The semi-rigid hood is pulled over the collar before donning and is secured by integrated straps.

**Figure 2.** Reported comfort (primary) and safety (secondary) outcomes for Bubble-PAPR vs usual FFP3 face masks.

**Figure 3.** Secondary communication outcomes where a higher Likert scale response was considered better.

**Figure S1. (Supplemental)** Participant job roles.

**Figure S2. (Supplemental)** Self-reported weight, height and BMI of staff participants.

**Figure S3. (Supplemental)** Secondary outcomes where a lower Likert scale response was considered better.

**Figure S4. (Supplemental)** Ease of donning and doffing of Respiratory Protective Equipment.

**Figure S5. (Supplemental).** Word clouds from the free text feedback. Negative comments (all categories).

**Figure S6 (Supplemental).** Word clouds from the free text feedback. Neutral comments (all categories).

**Figure S7 (Supplemental).** Word clouds from the free text feedback. Positive comments (all categories).



For peer review only - http://bmjopen.bmj.com/site/about/guidelines.xhtml

For peer review only

**References**


1. Fadela Chaib, World Health Organisation. Shortage of personal protective equipment endangering health workers worldwide. 3rd March 2020. Available from www.who.int/news/item/03-03-2020-shortage-of-personal-protective-equipment-endangering-health-workers-worldwide [Accessed 5th May 2020].

2. Hignett S, Welsh R, Banerjee J. Human factors issues of working in personal protective equipment during the COVID-19 pandemic. *Anaesthesia* 2021; 76: 134-135.

3. Cook TM. Personal protective equipment during the coronavirus disease (COVID) 2019 pandemic - a narrative review. *Anaesthesia* 2020; 75: 920-927.

4. British Standard BS EN12941 Respiratory protective devices - Powered filtering devices incorporating a helmet or a hood - Requirements, testing, marking (British Standard). 1999. Available from https://standards.globalspec.com/ [Accessed 5th April 2020].

5. Rizan C, Reed M, Bhutta MF. Environmental impact of personal protective equipment distributed for use by health and social care services in England in the first six months of the COVID-19 pandemic. *Journal of the Royal Society of Medicine* 2021; 114: 250-263.

6. Foo CC, Goon AT, Leow YH, Goh CL. Adverse skin reactions to personal protective equipment against severe acute respiratory syndrome--a descriptive study in Singapore. *Contact Dermatitis* 2006; 55: 291-294.

7. Turner MC, Duggan LV, Glezerson BA, Marshall SD. Thinking outside the (acrylic) box: a framework for the local use of custom-made medical devices. *Anaesthesia* 2020; 75: 1566-1569.

8. Gould CL, Alexander PDG, Allen CN, McGrath BA, Shelton CL. Protecting staff and patients during airway management in the COVID-19 pandemic: are intubation boxes safe. *British Journal of Anaesthesia* 2020; 125: e292-e293.

9. Shelton C, El-Boghdadly K, Appleby JB. The 'haves' and 'have-nots' of personal protective equipment during the COVID-19 pandemic: the ethics of emerging inequalities amongst healthcare workers. *Journal of Medical Ethics* 2021; medethics-2021-107501.

10. Great Britain Health and Safety Executive. Guidance on Respiratory Protective Equipment (RPE) Fit Testing: (Rev1). March 2019. Available from https://www.hse.gov.uk/pubns/indg479.htm [Accessed 5th April 2020].

11. Davis FD. Perceived Usefulness, Perceived Ease of Use, and User Acceptance of Information Technology. *MIS Quarterly* 1989; 13: 319-340.

12. European Agency for Health and Safety at Work. Regulation (EU) 2016/425 on Personal Protective Equipment. 2016. Available from https://osha.europa.eu/en/legislation/directive/regulation-eu-2016425-personal-protective-equipment. [Accessed 5th April 2020].

13. Duggan LV, Marshall SD, Scott J, Brindley PG, Grocott HP. The MacGyver bias and attraction of homemade devices in healthcare. *Canadian Journal of Anesthesia/Journal Canadien d'Anesthésie* 2019; 66: 757-761.

14. Addi RA, Benksim A, Cherkaaoui M. Easybreath Decathlon Mask: An Efficient Personal Protective Equipment (PPE) against COVID-19 in Africa. *Journal of Clinical and Experimental Investigations* 2020; 11: em00738.

15. Kim H, Hegde S, LaFiura C et al. Access to personal protective equipment in exposed healthcare workers and COVID-19 illness, severity, symptoms and duration: a population-based case-control study in six countries. *BMJ Global Health* 2021; 6: e004611.

16. Hoernke K, Djellouli N, Andrews L et al. Frontline healthcare workers' experiences with personal protective equipment during the COVID-19 pandemic in the UK: a rapid qualitative appraisal. *BMJ Open* 2021; 11: e046199.



For peer review only - http://bmjopen.bmj.com/site/about/guidelines.xhtml

17. Galanis P, Vraka I, Fragkou D, Bilali A, Kaitelidou D. Impact of personal protective equipment use on health care workers' physical health during the COVID-19 pandemic: A systematic review and meta-analysis. *American Journal of Infection Control* 2021; 49: 1305-1315.

18. Houghton C, Meskell P, Delaney H et al. Barriers and facilitators to healthcare workers' adherence with infection prevention and control (IPC) guidelines for respiratory infectious diseases: a rapid qualitative evidence synthesis. *Cochrane Database of Systematic Reviews* 2020; 4: CD013582.

19. Poostchi A, Kuet ML, Richardson PS, Patel MK. Covid-19: face masks can be devastating for people with hearing loss but alternatives are available. *BMJ* 2020; 370: m3326.

For peer review only


For peer review only - http://bmjopen.bmj.com/site/about/guidelines.xhtml

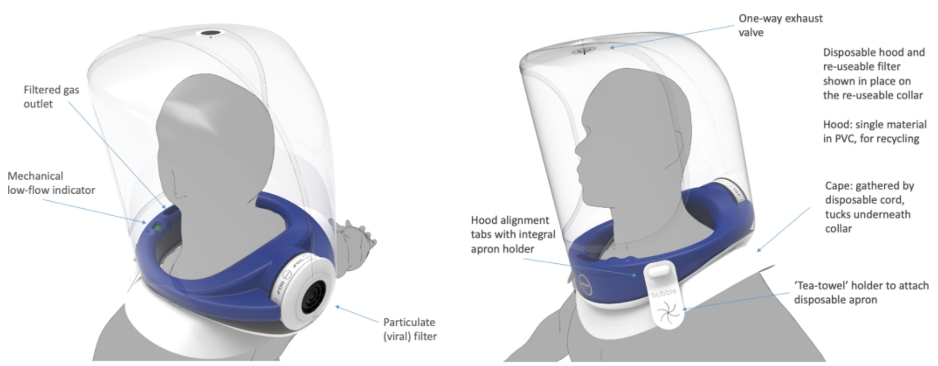


Bubble-PAPR comprises a medical-grade foam neck collar and a separate PVC hood. The universal fit collar draws air in through a filter via an impellor powered by an external battery. The collar has a mechanical low flow indicator and can be cleaned and reused by different users. The semi-rigid hood is pulled over the collar before donning and is secured by integrated straps.

762x283mm (72 x 72 DPI)

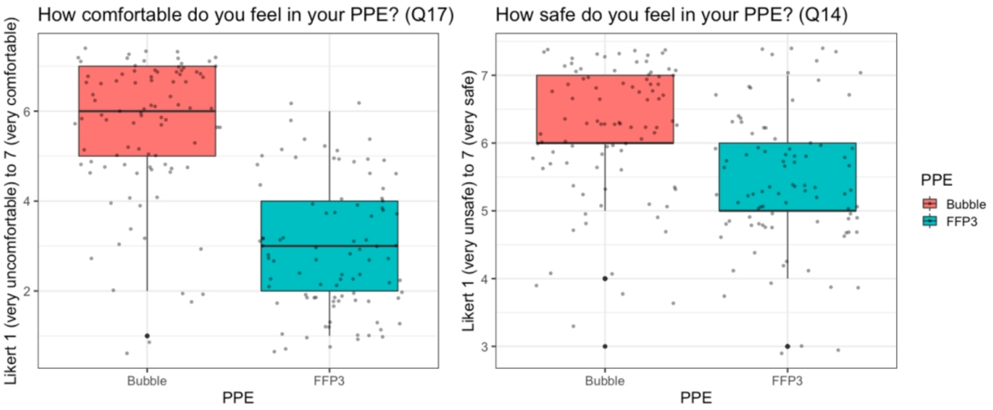


Reported comfort (primary) and safety (secondary) outcomes for Bubble-PAPR vs usual FFP3 face masks.

537x225mm (72 x 72 DPI)

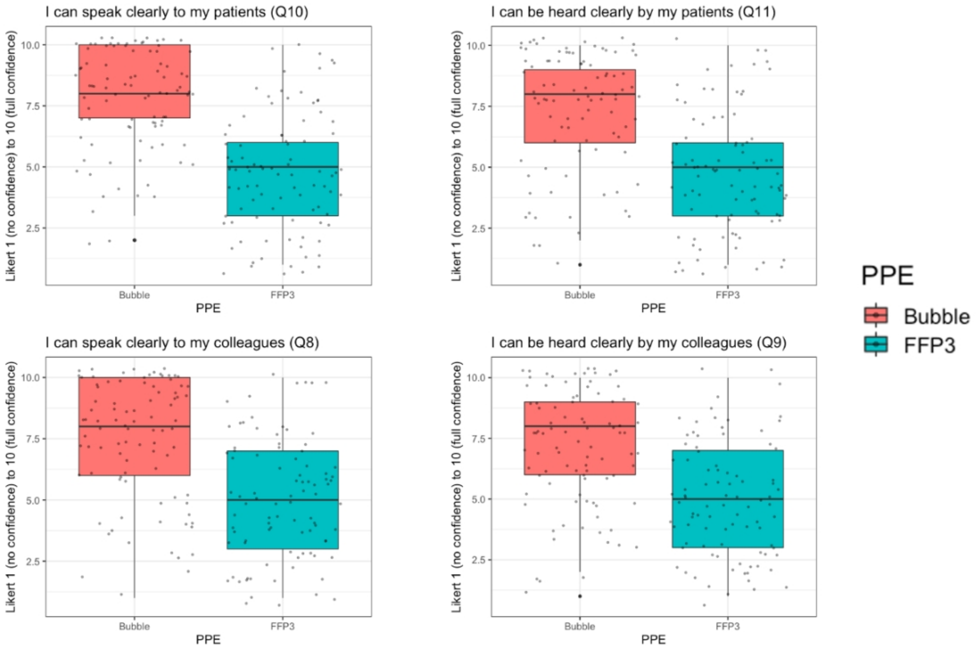


Secondary communication outcomes where a higher Likert scale response was considered better.

446x295mm (72 x 72 DPI)

**Table S1. (Supplemental)** Proposed framework for the safer adoption of a MacGyvered device. Adapted from Turner and colleagues.[7]

1. Define the problem and rule out the suitability of existing solutions
2. List benchmark safety indices for the device
3. Seek broader feedback from all stakeholders on the design's utility and potential pitfalls.
4. Perform laboratory-based and in situ simulations.
5. Introduce into low-risk clinical settings after local due process and patient consent.
6. Introduce into higher-risk clinical settings with a discrete group of trained 'super-users'.
7. Encourage an iterative cycle of feedback, review, re-design and improvement.
8. **Do not:** adopt, publish, endorse or disseminate via social media a MacGyvered device without data to support safety.

For peer review only


For peer review only - http://bmjopen.bmj.com/site/about/guidelines.xhtml

**Table S2. (Supplemental). Lab-based testing of the Bubble PAPR prior to clinical evaluation**

All of the bench tests detailed below were carried out by the Electrical and Biomedical Engineering (EMBE) team based at Wythenshawe Hospital (MFT), the University of Manchester Mechanical Aerospace and Civil Engineering team (UoM) or by INSPEC International, Salford, UK. A PAPR unit was supplied, and the Instructions for Use were followed by the independent tester, with a judgement made if they fulfilled particular requirements. Some requirements are supplemented by the qualitative or quantitative data collected in the questionnaires. Standards used were British Standard EN12941 (BS, 2008) and the European Regulations for Respiratory Protective Equipment EU2016/425 (ER, 2016).

| Relevant section of standard | Standard detail | Test location | Test detail | Results/notes | Pass/Fail |
|---|---|---|---|---|---|
| BS 6.1.1<br>ER 1.3.2 | Suitable resistance to wear and tear | MFT | PAPR units inspected after 1 week of continual use. Images taken before and after. | Opinion. Baseline inspection +/- photograph. Review after 1 week | Pass 14/3/21 |
| | | | | | |
| BS 6.1.4<br>ER 1.2.1.2 | No sharp edges | MFT | Visual and physical inspection Reports from staff evaluation | Opinion. Baseline inspection +/- photograph. Review after 1 week | Pass 14/3/21 |
| | | | | | |
| BS 6.3.2 | Fits a range of head sizes | MFT | Ten participants will undergo fit testing. These participants will have height, weight and head circumference measured as part of this standard process. | Fit test data shared with EBME team. All fit factors >500 as per BS EN 12941 standard. | Pass 25/2/21 |
| | | | | | |
| BS 6.3.3.1 | Does not distort vision | MFT | The optical area appears transparent. | Inspection by EBME team | Pass 25/2/21 |
| BS 6.3.3.2 | Permits appropriate field of view | MFT | Reports from staff evaluation | Review of results from initial staff evaluations | Pass 14/3/21 |


For peer review only - http://bmjopen.bmj.com/site/about/guidelines.xhtml

| ER2.3 | | | | | |
|---|---|---|---|---|---|
| Relevant section of standard | Standard detail | Test location | Test detail | Results/notes | Pass/Fail |
| BS 6.9 | Cannot reverse airflow | MFT | Normal use.<br>Simulate blocked filter and blocked air duct.<br>Flowmeter. | | Pass 14/3/21 |
| BS 6.9 | Battery safe – protection from short circuit | MFT | EBME check on battery packs (suitable for purpose / recommended packs) | As per manufacturer documentation. Will not be separately tested. | Pass 25/2/21 |
| | | | | | |
| ER2.12 | Appropriate markings | MFT | Yoke manufactured with section for appropriate sticker | | Pass 25/2/21 |
| | | | | | |
| ER3.10.1 | Appropriate training provided | MFT | Instructions for use provided.<br>Training videos provided. | Instructions for Use provided to EBME.<br>Training videos available. | Pass 25/2/21 |
| | | | | | |
| No specific clause | Cleanable | MFT | Specification of foam material for yoke details cleaning methods, durability and material fatigue. | Specification provided to EBME. | Pass 25/2/21 |
| BS 6.16 | Mass shall not exceed 5kg. A maximum of 1.5kg shall be carried on the head. | MFT | Weigh the assembled Bubble PAPR | Assembled Bubble PAPR Weight = 1.4kg | Pass 14/3/21 |

For peer review only


For peer review only - http://bmjopen.bmj.com/site/about/guidelines.xhtml

| Relevant section of standard | Standard detail | Test location | Test detail | Results/notes | Pass/Fail |
|---|---|---|---|---|---|
| BS 6.1.3 | Repeated cleaning and disinfection – does not deteriorate | MFT | PAPR units are inspected after 1 week of continual use. Images taken before and after.<br><br>Specification of foam material for yoke details cleaning methods, durability and material fatigue. | Spec sheet and review after 1 week of use. | Pass 14/3/21 |
| | | | | | |
| BS 6.4 | Ingress protection test for 10 test subjects – using either or both methods (bitter and/or particulometer). | MFT | 10 users will undergo fit testing with particulometer. | Fit test data shared with EBME team. All fit factors >500 as per BS EN 12941 standard. | Pass 25/2/21 |
| ER 3.10.2 | Appropriate protection to eye and skin irritants | MFT | | | |

For peer review only


For peer review only - http://bmjopen.bmj.com/site/about/guidelines.xhtml

| Relevant section of standard | Standard detail | Test location | Test detail | Results/notes | Pass/Fail |
|---|---|---|---|---|---|
| BS 6.2 (6.4) | Repeated after hood/yoke is conditioned at maximum specified temperature and humidity.<br><br>Complete unit is stored for 72 +/- 1 hours at the upper extreme of temperature and humidity specified by the manufacturer. Unit is allowed to return to ambient conditions for 4 hours, then stored for 72 +/- 1 hours at the lower extreme of temperature and humidity. | MFT | Unit is subjected to particulometer fit testing after appropriate temperature conditioning. | *Conditioning beyond use on the ICU should not be required for the MFT in-house evaluation*<br><br>All fit tests took place on the Acute ICU at Wythenshawe | Pass 25/2/21 |
| | | | | | |
| BS 6.5 | Positive pressure inside the hood remains below 5mbar | MFT | 1 user and 1 dummy test head setup. Pressure measurement inside hood during regular use. | Measured pressure below 5mBar | Pass 14/3/21 |
| BS 6.6.2 | Exceeds manufacturer's minimum specified airflow for a period of at least 4 hours.<br><br>(The UK/EU regs do not specify a minimal flow. US regulations do, but this is not immediately relevant) | MFT, INSPEC | Test head and flow meter.<br><br>*Note the flow meter arrangement is slightly complex as the measurement itself can interfere with flow.* | Breathe Safety report reviewed. | Pass 25/2/21 |


For peer review only - http://bmjopen.bmj.com/site/about/guidelines.xhtml

| Relevant section of standard | Standard detail | Test location | Test detail | Results/notes | Pass/Fail |
|---|---|---|---|---|---|
| BS 6.7 | Check function of minimum airflow indicator. | MFT, INSPEC | Apply different flow rates to the yoke measured by external flow meter and evaluate performance of the minimum airflow indicator in units. | Breathe Safety report reviewed. | Pass 25/2/21 |
| | | | | | |
| EN 143<br>BS 6.8 | Clogging of filter | MFT, INSPEC | Flow through filter and yoke tested after 4 hours of use with an external flow meter. | Breathe Safety report reviewed. | Pass 25/2/21 |
| | | | | | |
| 6.13 | The carbon dioxide content of the inhalation air (dead space inside the hood) shall not exceed an average of 1% by volume.<br><br>There is a specific test-rig setup for this. A physiological surrogate model should provide adequate assurance that the $CO_2$ content inside the hood is <1% during normal use. | MFT, INSPEC | Oxygen and carbon dioxide (gas analysis) inside the hood measured as partial pressures and/or percentages using MFT EBME equipment. | Breathe Safety report reviewed. | Pass 25/2/21 |

For peer review only


For peer review only - http://bmjopen.bmj.com/site/about/guidelines.xhtml

| Relevant section of standard | Standard detail | Test location | Test detail | Results/notes | Pass/Fail |
|---|---|---|---|---|---|
| BS 6.15 | Exhalation means (valve) maximum flowrate and safe operation:<br><br>Hood performs adequately during normal use. Specifically; exhalation means:<br>• functions and can be replaced (new hood)<br>• functions in orientations encountered during normal use<br>• is protected against dirt and mechanical damage | MFT | Test subject wears hood.<br><br>Eventually, this section is supplemented with reports from staff evaluation. | Review of initial feedback from users in sim setting | Pass 14/3/21 |
| BS 6.15 | Exhalation means (valve) maximum flowrate and safe operation:<br><br>Continuous flow rate of 300 +/- 15 L/min is applied for a period of 60+/- 6 secs. | MFT | Flow generator (ventilator) and flow meter.<br><br>Visual inspection of exhalation valve. | Opinion of EBME team. The flow generator works as described with the test flowmeter supplied. Exhalation Valve inspection pass. | Pass 14/3/21 |

For peer review only

**Table S3. (Supplemental). Pilot data. Q14 & Q17 are Likert Scale items (rated 1-7) and Q8-11 are Visual Analogue Scale items (rated 0-100).**

| | Current PPE | | | | | | BUBBLE-PAPR | | | | | |
|---|---|---|---|---|---|---|---|---|---|---|---|---|
| Participant | Q14 Safe to wear | Q17 Comfort to wear | Q8 Speak clearly to colleagues | Q9 Be heard by colleagues | Q10 Speak clearly to patients | Q11 Be heard by patients | Q14 Safe to wear | Q17 Comfort to wear | Q8 Speak clearly to colleagues | Q9 Be heard by colleagues | Q10 Speak clearly to patients | Q11 Be heard by patients |
| 1 | 3 | 3 | 10 | 20 | 0 | 0 | 4 | 5 | 75 | 75 | 80 | 80 |
| 2 | 4 | 2 | 15 | 20 | 10 | 30 | 7 | 5 | 85 | 80 | 60 | 70 |
| 3 | 4 | 4 | 20 | 20 | 30 | 30 | 5 | 6 | 90 | 90 | 90 | 90 |
| 4 | 5 | 2 | 25 | 25 | 10 | 10 | 6 | 6 | 95 | 95 | 95 | 85 |
| 5 | 5 | 4 | 33 | 40 | 25 | 25 | 6 | 5 | 75 | 70 | 85 | 90 |
| 6 | 6 | 3 | 30 | 25 | 30 | 30 | 6 | 5 | 65 | 70 | 55 | 66 |
| 7 | 7 | 2 | 20 | 30 | 25 | 25 | 7 | 5 | 70 | 70 | 65 | 65 |
| 8 | 4 | 3 | 45 | 50 | 20 | 30 | 7 | 6 | 90 | 95 | 90 | 90 |
| Mean | 4.8 | 2.9 | 24.8 | 28.8 | 18.8 | 22.5 | 6.0 | 5.4 | 80.6 | 80.6 | 77.5 | 79.5 |
| SD | 1.3 | 0.8 | 11.1 | 10.9 | 10.9 | 11.3 | 1.1 | 0.5 | 10.8 | 11.2 | 15.4 | 11.0 |


For peer review only - http://bmjopen.bmj.com/site/about/guidelines.xhtml

**Table S4. (Supplemental) Fit testing data from the first 10 participants.**

Test protocol HSE INDG 479. Pass level set at a fit factor of 500.

| Subject | Self-reported height | Self-reported weight | BMI | Normal Breathing 1 | Deep Breathing | Head Side to Side | Head Up and Down | Talking | Bending at the waist | Normal Breathing 2 | Overall Fit Factor |
|---|---|---|---|---|---|---|---|---|---|---|---|
| **1** | 1.86 | 74 | 21.4 | 79705 | 43647 | 125478 | 11125 | 107899 | 1339 | 76152 | **7757** |
| **2** | 1.95 | 75 | 19.7 | 53792 | 52343 | 59440 | 51673 | 52733 | 50433 | 45961 | **52075** |
| **3** | NR | NR | NR | 38867 | 36699 | 37097 | 41474 | 39500 | 36884 | 37465 | **38217** |
| **4** | 1.82 | 65 | 19.6 | 17745 | 6622 | 3149 | 5028 | 31996 | 30520 | 31326 | **8539** |
| **5** | 1.65 | 55 | 20.2 | 24945 | 25215 | 3885 | 8097 | 28877 | 29107 | 24393 | **12268** |
| **6** | 1.67 | 58 | 20.8 | 24617 | 25608 | 25581 | 25225 | 20107 | 1924 | 23517 | **9088** |
| **7** | 1.52 | 47 | 20.3 | 28747 | 30700 | 33203 | 15275 | 31671 | 8327 | 26041 | **19829** |
| **8** | 1.65 | 65 | 23.9 | 27282 | 31318 | 34900 | 9697 | 29093 | 3514 | 25770 | **12544** |
| **9** | 1.83 | 115 | 34.3 | 11182 | 1123 | 11028 | 24524 | 24692 | 2537 | 23704 | **4408** |
| **10** | 1.59 | 75 | 29.7 | 25760 | 3419 | 16125 | 2433 | 25523 | 1552 | 26154 | **4588** |
| | | | | | | | | | | | |
| **Mean values** | | | | **33264** | **25669** | **34989** | **19455** | **39209** | **16614** | **34048** | **16931** |


For peer review only - http://bmjopen.bmj.com/site/about/guidelines.xhtml

Figure S1. (Supplemental) Participant job roles.

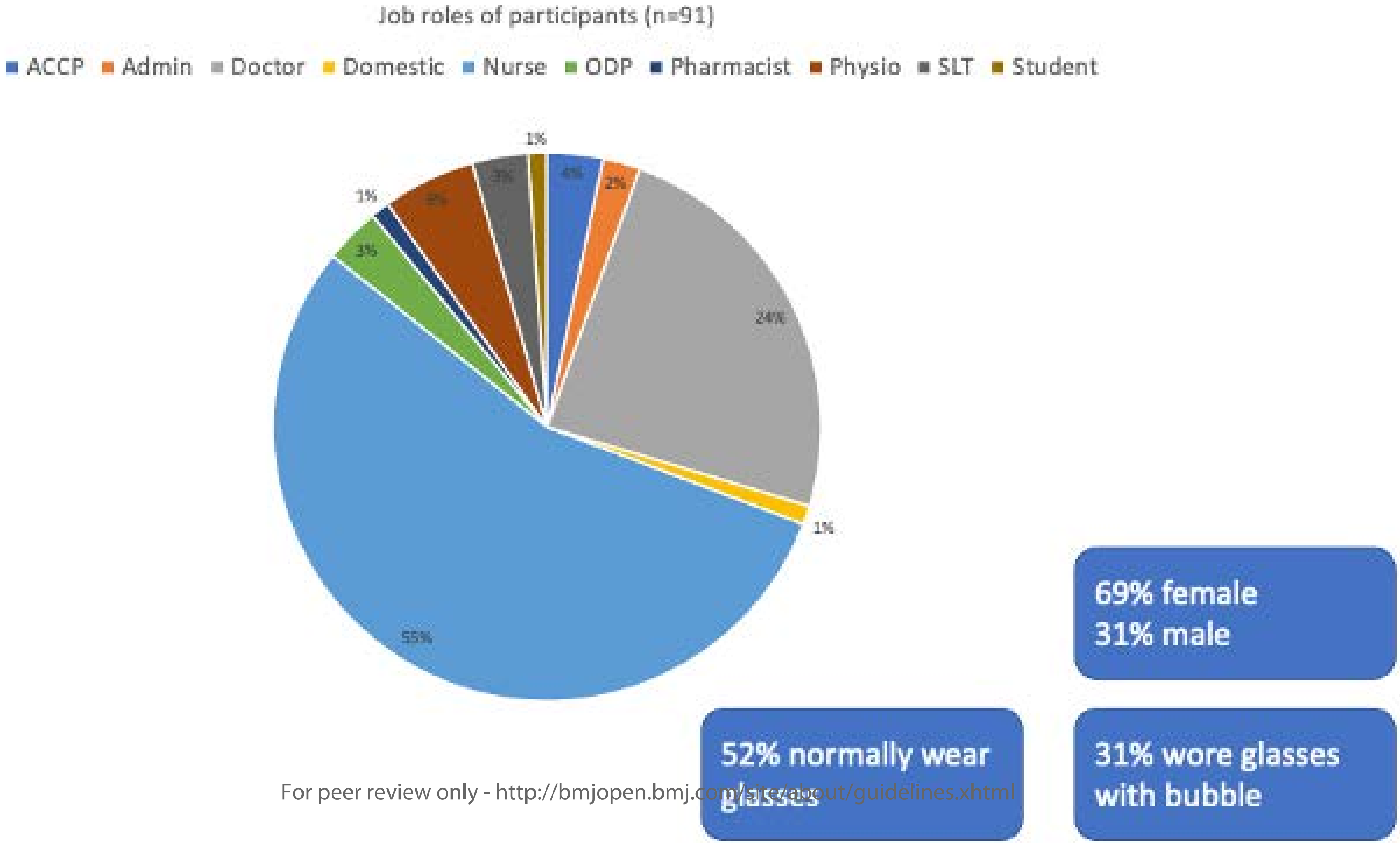

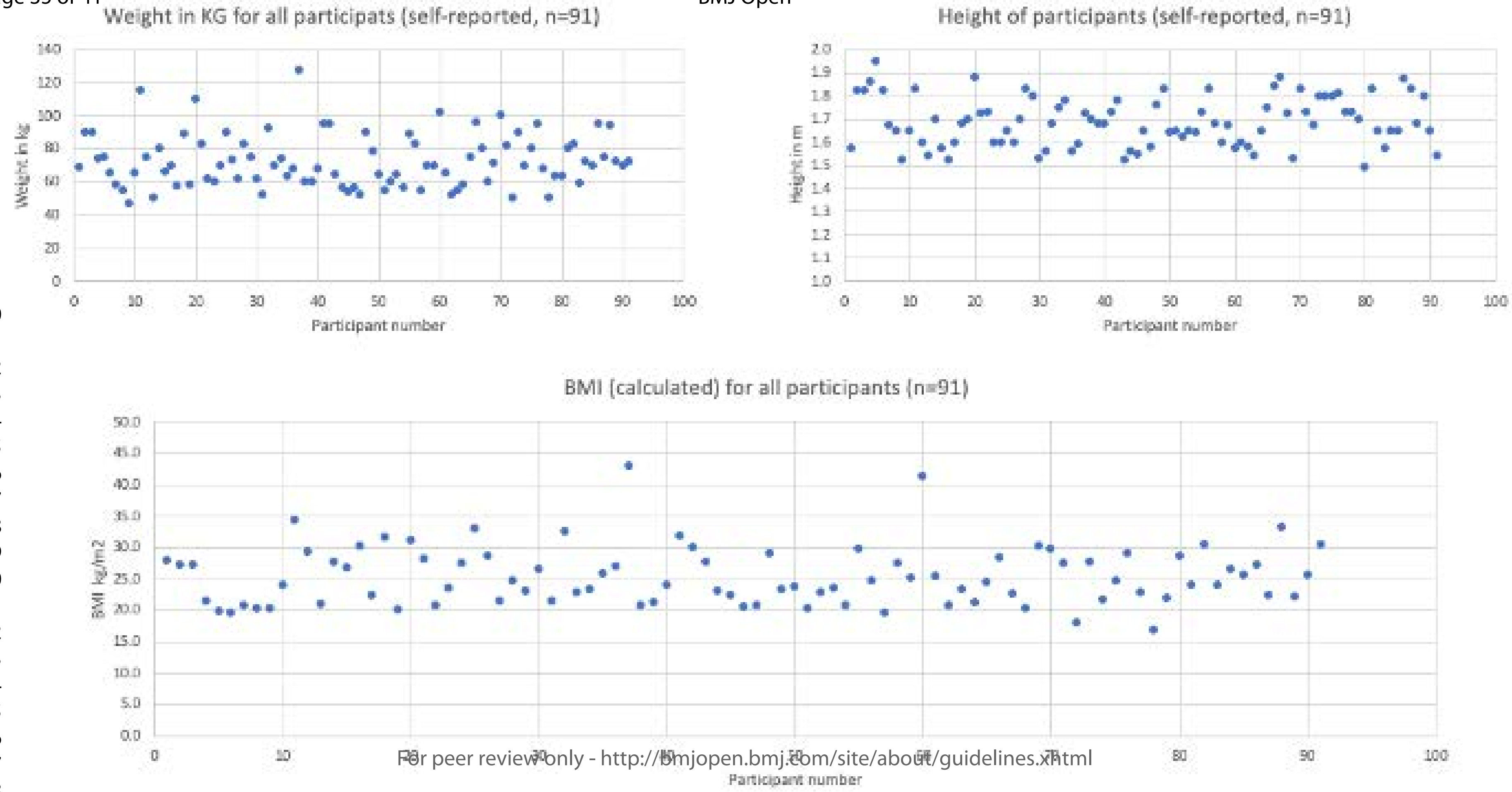


Figure S2. (Supplemental) Self-reported weight, height and BMI of staff participants.

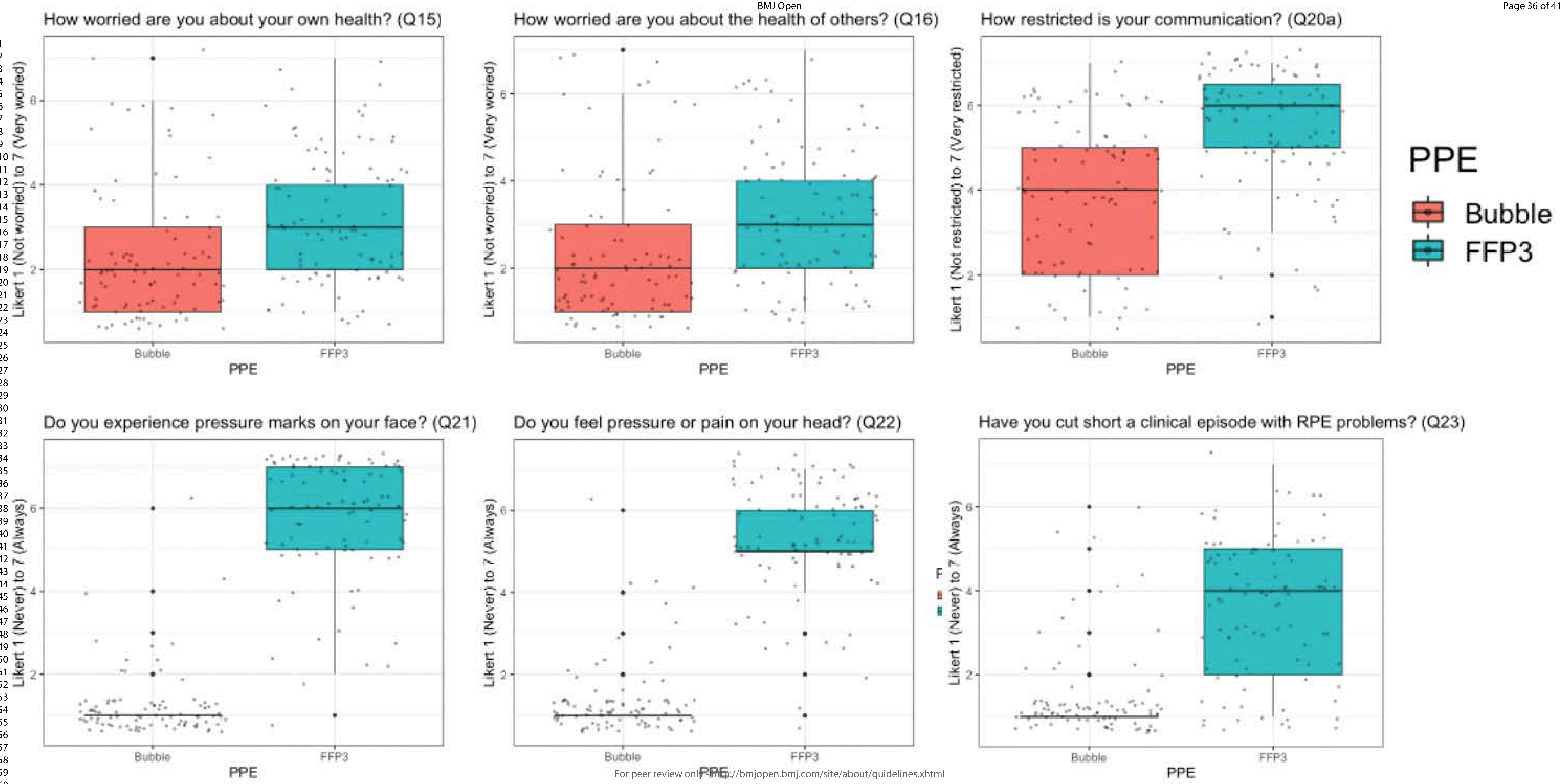




Figure S3. (Supplemental) Secondary outcomes where a lower Likert scale response was considered better.

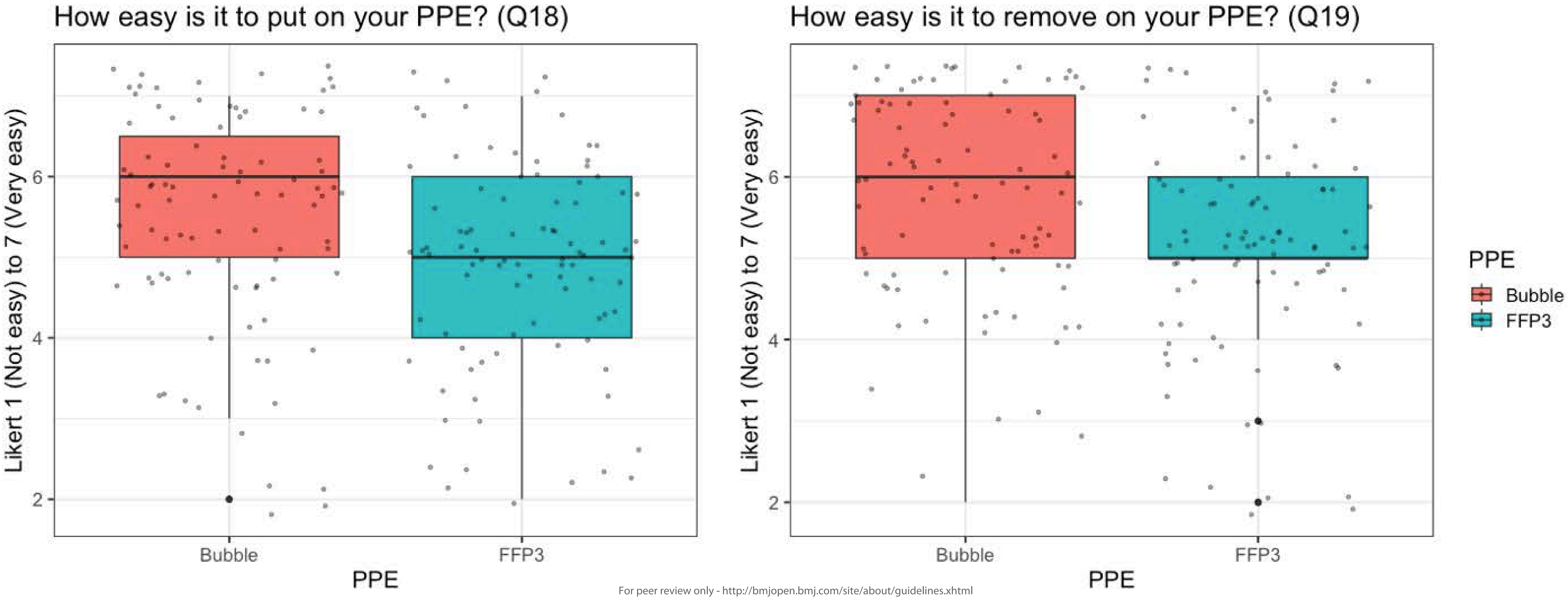


For peer review only - http://bmjopen.bmj.com/site/about/guidelines.xhtml

Figure S4. (Supplemental) Ease of donning and doffing of Respiratory Protective Equipment.

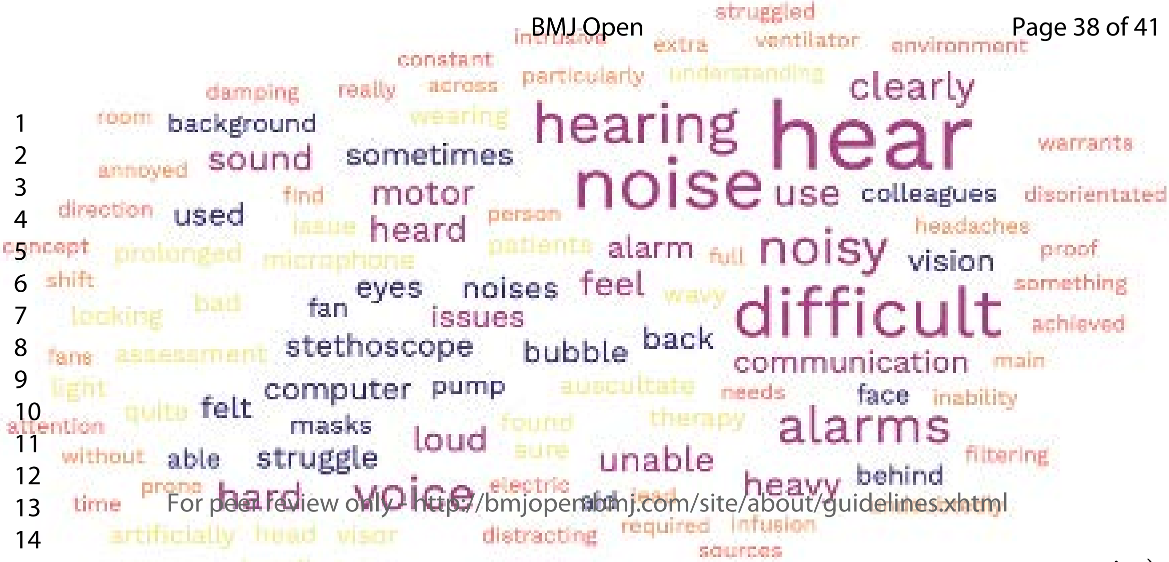


Figure S5. (Supplemental). Word clouds from the free text feedback. Negative comments (all categories).

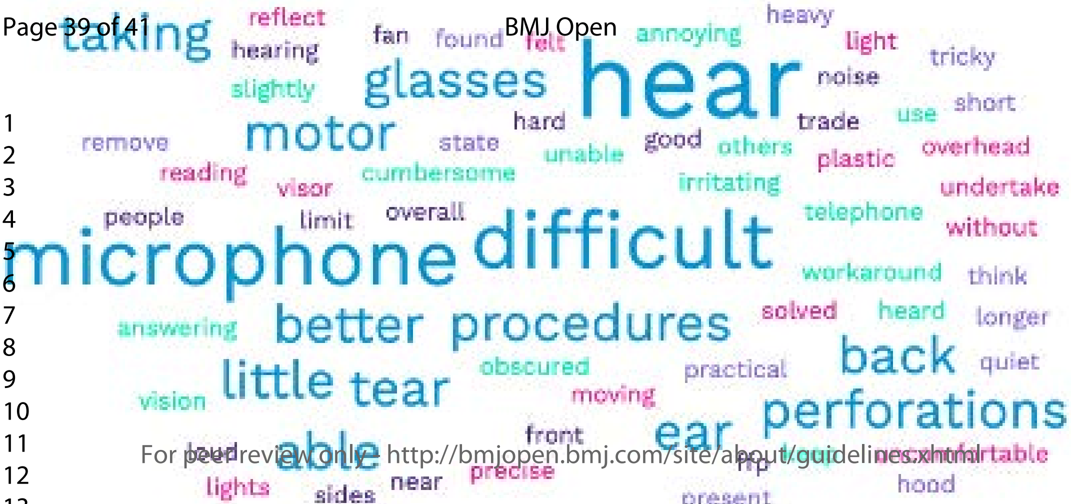




Figure S6 (Supplemental). Word clouds from the free text feedback. Neutral comments (all categories).

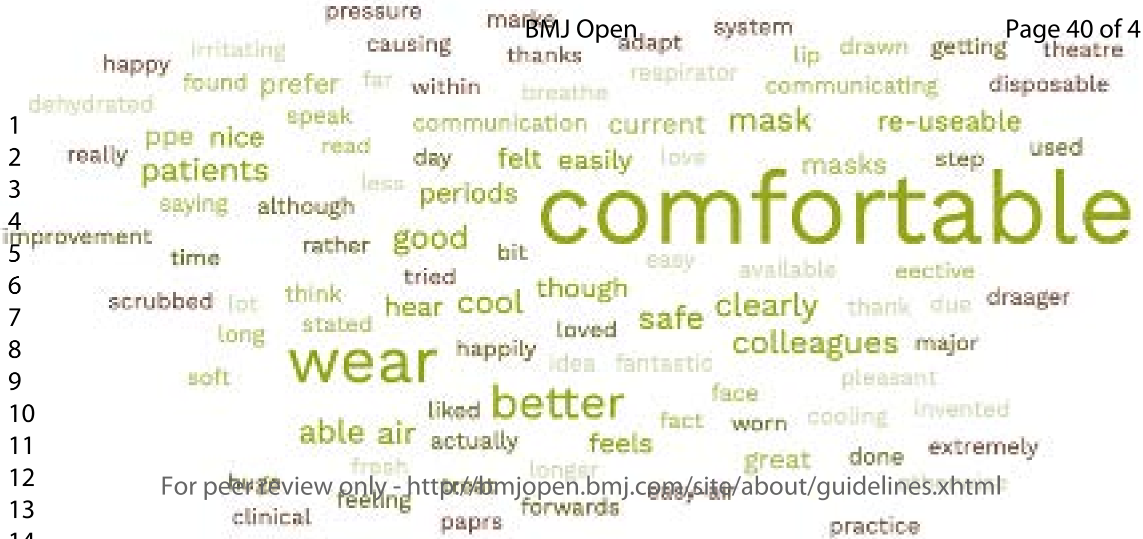


Figure S7 (Supplemental). Word clouds from the free text feedback. Positive comments (all categories).

STROBE Statement—checklist of items that should be included in reports of observational studies

| | Item No | Recommendation | Page No |
|---|---|---|---|
| **Title and abstract** | 1 | (*a*) Indicate the study's design with a commonly used term in the title or the abstract | 1 |
| | | (*b*) Provide in the abstract an informative and balanced summary of what was done and what was found | 2 |
| **Introduction** | | | |
| Background/rationale | 2 | Explain the scientific background and rationale for the investigation being reported | 3 |
| Objectives | 3 | State specific objectives, including any prespecified hypotheses | 4 |
| **Methods** | | | |
| Study design | 4 | Present key elements of study design early in the paper | 5 |
| Setting | 5 | Describe the setting, locations, and relevant dates, including periods of recruitment, exposure, follow-up, and data collection | 5 |
| Participants | 6 | (*a*) *Cohort study*—Give the eligibility criteria, and the sources and methods of selection of participants. Describe methods of follow-up<br>*Case-control study*—Give the eligibility criteria, and the sources and methods of case ascertainment and control selection. Give the rationale for the choice of cases and controls<br>*Cross-sectional study*—Give the eligibility criteria, and the sources and methods of selection of participants | 6 |
| | | (*b*) *Cohort study*—For matched studies, give matching criteria and number of exposed and unexposed<br>*Case-control study*—For matched studies, give matching criteria and the number of controls per case | |
| Variables | 7 | Clearly define all outcomes, exposures, predictors, potential confounders, and effect modifiers. Give diagnostic criteria, if applicable | 6 and Supplemental Material |
| Data sources/ measurement | 8* | For each variable of interest, give sources of data and details of methods of assessment (measurement). Describe comparability of assessment methods if there is more than one group | 6 |
| Bias | 9 | Describe any efforts to address potential sources of bias | 5 |
| Study size | 10 | Explain how the study size was arrived at | 6 |
| Quantitative variables | 11 | Explain how quantitative variables were handled in the analyses. If applicable, describe which groupings were chosen and why | 6 |
| Statistical methods | 12 | (*a*) Describe all statistical methods, including those used to control for confounding | 6 |
| | | (*b*) Describe any methods used to examine subgroups and interactions | N/A |


For peer review only - http://bmjopen.bmj.com/site/about/guidelines.xhtml

| | | | |
|---|---|---|---|
| | | (*c*) Explain how missing data were addressed | Intention to treat. No missing data. |
| | | (*d*) *Cohort study*—If applicable, explain how loss to follow-up was addressed<br>*Case-control study*—If applicable, explain how matching of cases and controls was addressed<br>*Cross-sectional study*—If applicable, describe analytical methods taking account of sampling strategy | |
| | | (*e*) Describe any sensitivity analyses | N/A |

| **Results** | | | |
|---|---|---|---|
| Participants | 13* | (a) Report numbers of individuals at each stage of study—eg numbers potentially eligible, examined for eligibility, confirmed eligible, included in the study, completing follow-up, and analysed | 7 |
| | | (b) Give reasons for non-participation at each stage | 7 |
| | | (c) Consider use of a flow diagram | Not required |
| Descriptive data | 14* | (a) Give characteristics of study participants (eg demographic, clinical, social) and information on exposures and potential confounders | 7 |
| | | (b) Indicate number of participants with missing data for each variable of interest | N/A |
| | | (c) *Cohort study*—Summarise follow-up time (eg, average and total amount) | 7 |
| Outcome data | 15* | *Cohort study*—Report numbers of outcome events or summary measures over time | 7 |
| | | *Case-control study*—Report numbers in each exposure category, or summary measures of exposure | |
| | | *Cross-sectional study*—Report numbers of outcome events or summary measures | |
| Main results | 16 | (*a*) Give unadjusted estimates and, if applicable, confounder-adjusted estimates and their precision (eg, 95% confidence interval). Make clear which confounders were adjusted for and why they were included | 7 |
| | | (*b*) Report category boundaries when continuous variables were categorized | 7 |
| | | (*c*) If relevant, consider translating estimates of relative risk into absolute risk for a meaningful time period | 7 |
| Other analyses | 17 | Report other analyses done—eg analyses of subgroups and interactions, and sensitivity analyses | 8 |
| **Discussion** | | | |
| Key results | 18 | Summarise key results with reference to study objectives | 9 |
| Limitations | 19 | Discuss limitations of the study, taking into account sources of potential bias or imprecision. Discuss both direction and magnitude of any potential bias | 9 |
| Interpretation | 20 | Give a cautious overall interpretation of results considering objectives, limitations, multiplicity of analyses, results from similar studies, and other relevant evidence | 9 |
| Generalisability | 21 | Discuss the generalisability (external validity) of the study results | 10,11 |
| **Other information** | | | |
| Funding | 22 | Give the source of funding and the role of the funders for the present study and, if applicable, for the original study on which the present article is based | 12 |

For peer review only


For peer review only - http://bmjopen.bmj.com/site/about/guidelines.xhtml

BMJ Open

# Bubble-PAPR: Phase I clinical evaluation of the comfort and perception of a prototype powered air-purifying respirator for use by healthcare workers in an acute hospital setting.

| | |
|---|---|
| Journal: | *BMJ Open* |
| Manuscript ID | bmjopen-2022-066524.R2 |
| Article Type: | Original research |
| Date Submitted by the Author: | 28-Feb-2023 |
| Complete List of Authors: | McGrath, Brendan; Manchester University NHS Foundation Trust; Manchester Academic Health Science Centre, Immunity and Respiratory Medicine<br>Shelton, Clifford; NHS Manchester; Lancaster University Lancaster Medical School<br>Gardner, Angela; Manchester University NHS Foundation Trust<br>Coleman, Ruth; Manchester University NHS Foundation Trust<br>Lynch, James; Manchester University NHS Foundation Trust<br>Alexander, Peter G; Manchester University NHS Foundation Trust; Manchester Academic Health Science Centre, Immunity and Respiratory Medicine<br>Cooper, Glen; The University of Manchester School of Mechanical Aerospace and Civil Engineering |
| <b>Primary Subject Heading</b>: | Intensive care |
| Secondary Subject Heading: | Anaesthesia, Communication, Infectious diseases, Intensive care, Nursing |
| Keywords: | OCCUPATIONAL & INDUSTRIAL MEDICINE, INTENSIVE & CRITICAL CARE, Infection control < INFECTIOUS DISEASES, ANAESTHETICS, RESPIRATORY MEDICINE (see Thoracic Medicine) |
| | |

SCHOLARONE™
Manuscripts

*I, the Submitting Author has the right to grant and does grant on behalf of all authors of the Work (as defined in the below author licence), an exclusive licence and/or a non-exclusive licence for contributions from authors who are: i) UK Crown employees; ii) where BMJ has agreed a CC-BY licence shall apply, and/or iii) in accordance with the terms applicable for US Federal Government officers or employees acting as part of their official duties; on a worldwide, perpetual, irrevocable, royalty-free basis to BMJ Publishing Group Ltd ("BMJ") its licensees and where the relevant Journal is co-owned by BMJ to the co-owners of the Journal, to publish the Work in this journal and any other BMJ products and to exploit all rights, as set out in our licence.*

*The Submitting Author accepts and understands that any supply made under these terms is made by BMJ to the Submitting Author unless you are acting as an employee on behalf of your employer or a postgraduate student of an affiliated institution which is paying any applicable article publishing charge ("APC") for Open Access articles. Where the Submitting Author wishes to make the Work available on an Open Access basis (and intends to pay the relevant APC), the terms of reuse of such Open Access shall be governed by a Creative Commons licence – details of these licences and which Creative Commons licence will apply to this Work are set out in our licence referred to above.*

*Other than as permitted in any relevant BMJ Author's Self Archiving Policies, I confirm this Work has not been accepted for publication elsewhere, is not being considered for publication elsewhere and does not duplicate material already published. I confirm all authors consent to publication of this Work and authorise the granting of this licence.*

For peer review only

**Title:**
Bubble-PAPR: Phase I clinical evaluation of the comfort and perception of a prototype powered air-purifying respirator for use by healthcare workers in an acute hospital setting.
**REVISION**

**Short title:**
Bubble-PAPR: a phase 1 study

**Authors**
Brendan A McGrath, Consultant & Honorary Professor[1,2]
Cliff Shelton, Consultant Clinical Senior Lecturer[1,3]
Angela Gardner, Clinical Fellow[1]
Ruth Coleman, Advanced Critical Care Practitioner[1]
James Lynch, Advanced Critical Care Practitioner[1]
Peter G Alexander, Consultant & Honorary Senior Lecturer[1,2]
Glen Cooper, Reader

1. Acute Intensive Care Unit, Wythenshawe Hospital, Manchester University NHS Foundation Trust
2. Manchester Academic Critical Care, Division of Infection, Immunity and Respiratory Medicine, School of Biological Sciences, Faculty of Biology, Medicine and Health, the University of Manchester, Manchester Academic Health Science Centre.
3. Lancaster Medical School, Lancaster University
4. Department of Mechanical, Aerospace & Civil Engineering, the University of Manchester

**Corresponding author**
Brendan A McGrath (CI)
Acute Intensive Care Unit
Wythenshawe Hospital
Southmoor Road
Wythenshawe
Manchester. M23 9LT
0161 291 6420
Brendan.mcgrath@manchester.ac.uk


For peer review only - http://bmjopen.bmj.com/site/about/guidelines.xhtml

**Structured abstract**

**Objectives**: We aimed to design and produce a low-cost, ergonomic, hood-integrated Powered Air-Purifying Respirator (Bubble-PAPR) for pandemic healthcare use, offering optimal and equitable protection to all staff. We hypothesised that participants would rate Bubble-PAPR more highly than current FFP3 face mask respiratory protective equipment (RPE).

**Design**: Rapid design and evaluation cycles occurred based on the identified user needs. We conducted diary card and focus group exercises to identify relevant tasks requiring RPE. Lab-based safety standards established against British Standard BS-EN-12941 and EU2016/425 covering materials; inward particulate leakage; breathing resistance; clean air filtration and supply; carbon dioxide elimination; exhalation means; and electrical safety. Questionnaire-based usability data from participating frontline healthcare staff before (usual RPE) and after using Bubble-PAPR.

**Setting**: Overseen by a trial safety committee, evaluation progressed sequentially through laboratory, simulated, low-risk, then high-risk clinical environments of a single tertiary NHS hospital.

**Participants:** 15 staff completed diary cards and focus groups. 91 staff from a range of clinical and non-clinical roles completed the study, wearing Bubble-PAPR for a median of 45 minutes (IQR 30-80 [15-120]). Participants self-reported a range of heights (mean 1.7m [SD 0.1, range 1.5-2.0]), weights (72.4kg [16.0, 47-127]) and body mass indices (25.3 [4.7,16.7-42.9]).

**Outcome measures**: Pre-use particulometer "fit testing" and evaluation against standards by independent biomedical engineer. Primary: perceived comfort (Likert scale). Secondary: perceived safety, communication.

**Results**: Mean fit factor 16,961 (ten participants). Bubble-PAPR mean comfort score 5.64(SD 1.55) versus usual FFP3 2.96(1.44) (mean difference 2.68 (95% CI 2.23-3.14, p<0.001). Secondary outcomes, Bubble-PAPR mean(SD) vs FFP3 mean(SD), [mean difference (95%CI)] were: *How safe do you feel?* 6.2(0.9) vs 5.4(1.0), [0.73 (0.45-0.99)]; *Speaking to other staff* 7.5 (2.4) vs 5.1 (2.4), [2.38 (1.66-3.11)]; *Heard by other staff* 7.1 (2.3) vs 4.9(2.3), [2.16 (1.45-2.88)]; *Speaking to patients* 7.8(2.1) vs 4.8(2.4), [2.99 (2.36-3.62)]; *Heard by patients* 7.4(2.4) vs 4.7(2.5), [2.7(1.97-3.43)]; all p<0.01.

**Conclusions**: Bubble-PAPR achieved its primary purpose of keeping staff safe from airborne particulate material whilst improving comfort and the user experience when compared with usual FFP3 masks. The design and development of Bubble-PAPR were conducted using a careful evaluation strategy addressing key regulatory and safety steps.


**Trial registration**: IRAS ID:288493, REC Ref:21/WA/0018. ClinicalTrials.gov (NCT04681365).



For peer review only - http://bmjopen.bmj.com/site/about/guidelines.xhtml

**Strengths and limitations of this study**

- We employed user-centred design, engineering optimisation and staged feasibility testing to develop a novel Powered Air-Purifying Respirator (Bubble-PAPR) for use specifically in frontline healthcare settings.
- The design of Bubble-PAPR met regulatory standards and our evaluation demonstrated that it met the key requirements of comfort and perceived safety identified as essential requirements by healthcare staff.
- The design and development of Bubble-PAPR were conducted using a careful strategy addressing key regulatory and safety steps, measured against UK/European standards, in contrast to many devices rapidly developed and deployed during the pandemic.
- Limitations of our study include: design and evaluation undertaken at a single large hospital, using similar staff groups; lack of formal independent cost analysis.
- Bubble-PAPR is an excellent example of developing a cosmopolitan network (social networks across historical, political, and cultural boundaries). These networks could become a key feature of future system resilience.

For peer review only


For peer review only - http://bmjopen.bmj.com/site/about/guidelines.xhtml

## Introduction

The COVID-19 global pandemic created a worldwide shortage of personal protective equipment (PPE)[1] and highlighted significant usability issues in current PPE products.[2] In addition to direct contact, airborne diseases may be spread by aerosol or droplet transmission. Aerosol transmission may be mitigated by the appropriate use of respiratory protective equipment (RPE), a particular classification of personal protective equipment (PPE). However, respiratory protective equipment is used as part of a hierarchy of control measures. This is because RPE only protects individual workers, is prone to failure or misuse (wrong RPE for the wrong task/environment) and wearers may get a false sense of security, which may lead to neglect of other aspects of infection prevention and control, such as isolation requirements.[3] A range of inspiratory filtering devices exist: dust masks, half-face masks, full-face masks and powered (fan-assisted) respirators. Powered respirators include: half/full-face masks, helmets, hoods and visors. Though not used in healthcare, for completeness, breathing apparatuses are systems that supply an independent, positive pressure supply of breathing-quality air.

Face masks may be classified by considering the level of protection they offer the wearer to inhalation of environmental contaminants. Simple surgical face masks or 'nuisance' dust masks do not entirely filter droplets or aerosols. Filtering face piece (FFP) masks comprise layers of synthetic non-woven material with interleaved filtration layers and provide protection against small airborne particles (aerosols). Different types and constructions of FFP masks can be classified by their ability to filter small particles. Particulate filters can be classified as low (P1) to high (P3) efficiency, filtering between 80% of particles smaller than 2 micrometres to 99.95% of particles smaller than 0.5 micrometres, respectively (Table 1).[4] Respiratory protection can therefore be considered in terms of a combination of the filtering ability of the device relative to the exposure environment and its fit on the wearer's face. A device is considered adequate if it has the capacity to reduce the wearer's exposure to a hazardous substance to acceptable levels (to comply with occupational exposure limit values). Devices can be reusable, but the majority are single-use. Masks are difficult to recycle due to their layered construction and the pandemic contributed to an unprecedented rise in RPE-related clinical waste.[5]

The majority of RPE used in healthcare settings are disposable face masks adopted from industry. Masks are not designed to be worn for long periods or repeated shifts, may restrict the visual field, limit communication, cause facial damage due to their tight fit, and require multiple time-consuming 'fit tests' for each model of the device for each staff member. All these issues were highlighted in the context of the 2002-2004 SARS epidemic.[6] More appropriate solutions for prolonged and repeated use include powered air-purifying respirators (PAPRs). But, again, these are not designed primarily for healthcare, are heavy, noisy, expensive, difficult to clean to clinical standards, and not suitable for the specific needs in frontline healthcare environments.

There have been several widely reported 'homemade' or 'MacGyvered' devices that well-intentioned groups or individuals developed to protect staff and patients during the pandemic.[7] In a time of crisis, these innovations were often rapidly developed without significant funding and delivered to areas of need during a time of global RPE shortage. However, due to the urgency of the situation, few of these devices sought or achieved independent certification or provided data to support safety.[8] Turner and colleagues proposed a framework for the safer adoption of novel devices[7] which: defines the problem and reviews existing solutions, benchmarks safety indices for the devices, and then evaluates it in a structured manner through simulated, low- and then high-risk clinical settings Table S1



For peer review only - http://bmjopen.bmj.com/site/about/guidelines.xhtml

(Supplemental). Broad stakeholder feedback is encouraged through iterative review cycles, re-design and improvements.

**Table 1.** Classification of particulate filters, with a worked example and fit testing. Data from EU Standard 149:2001 Respiratory Protective Devices.

**P1** – Filters about 80% of particles smaller than 2 micrometres
**P2** – Filters about 94% of particles smaller than 0.5 micrometres
**P3** – Filters about 99.95% of particles smaller than 0.5 micrometres

A respiratory protective device is considered adequate if it has the capacity to reduce the wearer's exposure to a hazardous substance to acceptable levels. The ratio of airborne particles outside:inside the filtering device gives a **nominal (theoretical) protection factor**. An **assigned protection factor** reflects the actual workplace conditions. For example: an airborne dust contaminant with an occupational exposure limit of 5mg/m$^3$ may be present in the workplace in concentrations up to 60mg/m$^3$ (determined by monitoring). A particle filter is needed to reduce the concentration by at least a factor of 12 (60/5=12). A P3 filter with an assigned protection factor of 20 would be suitable (as this is greater than the factor of 12 required). Other considerations such as exposure time, useability and disposal of the device need to be considered prior to undertaking a **fit test** with the intended wearer.

A fit test verifies that a **specific model** of device works as intended with a **particular individual**. For example, different face shapes and facial hair can interfere with a particular system's ability to filter environmental contaminants effectively.

**Qualitative fit testing** assesses the inward leakage past a mask of airborne compounds detectable by the wearer (typically bitter/sweet tasting substances), aerosolised using a spray device.

**Quantitative fit testing** measures particulate concentrations inside and outside of devices, typically undertaken by measuring sodium chloride aerosolised in water to generate a 'particle' count. Quantitative fit testing generates a **fit factor** – the ratio of airborne particle counts outside:inside. The fit factor takes account of the whole device (the filter, hood and airflow in the case of a PAPR). Fit factors for PAPRs are very high (optimal protection) and so if correctly worn, fit testing prior to use is not usually required.

Considering the above, our project aimed to design and produce a low-cost, ergonomic, hood-integrated PAPR for use in frontline healthcare settings. Our objectives were to focus on user-centred design, engineering optimisation, staged feasibility testing, certification, intellectual property protection and then rapid manufacture and distribution. We also aimed to design the PAPR to be re-used, refurbished and recycled where possible, using readily available, simple and interchangeable key parts which proved difficult to source during the early stages of the pandemic. Finally, by designing an available, affordable PAPR system that could be cleaned appropriately and re-used between different staff, we aimed to provide equitable access to high-quality RPE that offered optimal protection to *all* staff, wherever they worked.[9] We hypothesised that participants would rate Bubble-PAPR more highly than current FFP3 face mask RPE across the domains of comfort, perceived safety and communication.



For peer review only - http://bmjopen.bmj.com/site/about/guidelines.xhtml

## Methods

The design team brought together frontline clinical staff based in the Wythenshawe Hospital Acute Intensive Care Unit (ICU) of Manchester University NHS Foundation Trust (MFT), an experienced product design consultancy (Designing Science Limited, Middlesex, UK) and the technical expertise of the School of Engineering at the University of Manchester (UoM). Research Ethical and Health Research Authority approval (IRAS ID:288493, REC Ref:21/WA/0018) was granted. The study was sponsored by MFT, who acted as the manufacturer of this in-house prototype device, which became known as Bubble-PAPR. The study protocol, analysis plan and recruitment metrics were registered and reported at ClinicalTrials.gov (NCT04681365). Participating staff were provided with participant information sheets, a detailed explanation and demonstration of the safe use of Bubble-PAPR, and written consent was obtained. User needs assessment was conducted through a series of workplace diary card exercises documenting typical activities undertaken by frontline healthcare staff, synthesised in focus groups. Staff were invited to participate (by email and posters in rest areas) from clinical locations where RPE was mandated within the hospital. The first two respondents from each area were recruited to the diary card and focus group activities. Rapid design and evaluation cycles occurred based on the identified user needs. In addition, evaluation of early prototypes occurred in simulated clinical environments, collecting usability data from participants.

### Patient and public involvement

Public and Patient involvement was undertaken through the Manchester Academic Critical Care research group's patient forum. There were powerful accounts from patients who regularly described not being able to understand what hospital staff wearing PPE were saying and being troubled that they had no idea what their carers looked like. These reports led us to focus on prioritising the ease of communication with Bubble-PAPR. Staff participants who were invited to wear Bubble-PAPR were recruited from clinical locations where RPE was mandated, by direct invitation from the research team.

A Trial Safety Committee was established to oversee the results of laboratory and bench testing of the prototype, initial safety data, usability, and adverse event data at each stage of the evaluation. The Committee met prior to commencing clinical evaluation. It was tasked with the decision to allow the evaluation to proceed between phases: simulated clinical environment, low-risk (non-infectious) clinical environment and high-risk clinical environment (COVID-19 wards and ICUs). Early iterations of Bubble-PAPR included 3-D-printed collars and key parts (such as the impellor), along with a variety of designs of the hood. A final iteration of Bubble-PAPR included a medical-grade foam collar, precision-machined internal components and a revised (smaller) hood was further tested in high-risk environments. Prior to first use, several device safety checks were independently undertaken by the MFT Electrical and Biomedical Engineering Department and INSPEC International Ltd, Salford, UK). A short report addressing the qualitative and qualitative criteria detailed in the relevant standards, and summarised in Table S2, S3 and S5 (Supplemental), was presented to the Trial Safety Committee. The first ten study participants to wear Bubble-PAPR underwent 'fit testing' with a particulometer (TSI Portacount Fit Tester 8040, TSI Instruments Ltd, Buckinghamshire, UK) following a standard protocol derived from the UK Government's Health and Safety Executive.[10] Fit testing is not required before wearing PAPRs, including Bubble-PAPR. The purpose of fit testing was to collect device performance data and to allow the research team to assure the Trial Safety Committee that Bubble-PAPR was



For peer review only - http://bmjopen.bmj.com/site/about/guidelines.xhtml

performing to an appropriate standard. This INDG-479 protocol requires a 'Fit Factor' pass level of 100 for FFP3/N95 face masks and 500 for full face masks/hoods. Participants followed this standard protocol during quantitative fit testing which involved the following exercises undertaken for at least 60 seconds: normal breathing; deep breathing; turning head from side-to-side; moving head up and down; talking; bending over to 90 degrees; repeat normal breathing. European Conformity Standard EN12941 requires an applied fit factor of 40 for a 'loose-fitting hood' PAPR; the equivalent of a nominal protection factor of at least 500 (accepting an inward leakage of 0.2% with a P3 class filter. See Table 1). By comparison, the minimal fit factor for an FFP3 mask in a clinical environment is 100. Tests were conducted in an ICU side room with a particle generator to reach background counts between 70,000 to 100,000 particles/$cm^3$.

The primary outcome was based on Davis' technology acceptance model (perceived usefulness and perceived ease-of-use overcoming barriers to adoption)[11]. First, staff were asked to rate their experiences using current RPE (a variety of re-useable or disposable FFP3 masks) using a series of questions based on Likert-type scales (see Supplemental material). Next, safe use of the Bubble-PAPR was explained, and instructions for use were provided, supported by videos of donning, doffing, cleaning and storage. Bubble-PAPR was then worn during simulated/clinical use where the usual tasks were undertaken (identified in the focus groups, including verbal communication between colleagues and patients; writing; typing; reading notes, computer screens and monitors; manual handling; invasive procedures; emergency resuscitation; airway management; and maintenance of a clean/safe bedside environment). In order to evaluate critical communication and the stability of the Bubble-PAPR, the simulated environment tests also included high-stakes team-based tasks such as managing a cardio-respiratory arrest, cardiopulmonary resuscitation, assessment and management of the critically ill patient and complex airway management. Finally, after removal (doffing) of Bubble-PAPR, staff were immediately invited to complete a second questionnaire focused on the prototype. Free text comments were also invited.

The primary endpoint was staff rating of the comfort of Bubble-PAPR (versus current FFP3 face masks). Secondary endpoints focused on communication and perceived safety. Specifically, this was staff ratings of the prototype in terms of: how safe participants felt, ease of communication with colleagues, and ease of communication with patients (again, Bubble-PAPR versus current FFP3 face masks). Additional questions explored wearer anxiety, ease of use, and performance whilst undertaking usual work tasks. In parallel, in-house device feasibility testing was conducted in the hospital environment to test ergonomics and air particle filtration. The research framework for this study was based around in-house exemption for device development from the UK Medicines and Healthcare products Regulatory Agency (MHRA). This means that the hospital, acting as manufacturer, can use a device it has developed itself internally. Such a device is not required to undergo to independent testing and therefore it will not achieve a certificate of conformity (UK-Conformity Assessed or Conformitè Europëenne marking). However, in order to assure the study Sponsor and staff participants of the safety and efficacy of Bubble-PAPR, we tested against existing conformity standards for PAPRs relevant at the time of development (British Standard BS EN 12941 [Respiratory Protective Devices: Powered filtering devices incorporating a helmet or hood] and the European Union Personal Protective Equipment Directive EU2016/425). [4,12] Some of the testing was undertaken internally by independent biomedical engineers, with the flowrate and carbon dioxide testing undertaken externally.


For peer review only - http://bmjopen.bmj.com/site/about/guidelines.xhtml

A pilot evaluation was conducted in August 2020 to test the questionnaires and to assess the likely population means for the test scores (Table S3 Supplemental). We calculated a sample size of 20 participants would be required for each phase of the evaluation to detect a significant difference between usual PPE and Bubble-PAPR, based on a mean difference of 2.5 (SD 0.9) points on the 7-point Likert scale identified during the pilot evaluation (alpha = 0.05, 90% power). In addition, we allowed for a 5% dropout and missing data rate, concluding 22 participants per phase. All variables were explored via appropriate graphical and descriptive statistics to evaluate distributions, data completeness and form. Analyses were conducted in RStudio 2020 (Boston, MA, www.rstudio.com). Analyses were performed separately for each phase for presentation to the Trial Safety Committee, with a pooled analysis conducted at the study conclusion. Comparisons between groups (current RPE vs Bubble-PAPR) were made using a paired t-test or Wilcoxon signed-rank test as appropriate.

For peer review only



For peer review only - http://bmjopen.bmj.com/site/about/guidelines.xhtml

**Results**

The final design of Bubble-PAPR is shown schematically in Figure 1 (www.bubble-papr.com, with detailed technical drawings available by searching the patent number [PCT/GB2021/052147] at www.espacenet.com). The device safety checks and fit testing results are presented in Tables S2, S3 and S4 (Supplemental), respectively, demonstrating a mean fit factor of 16,961. Additional particulometer tests were undertaken with deliberate tears up to 20 cm in the hood using a dummy head. The lowest fit factor recorded with the damaged hood was 1,123. Therefore, the Trial Safety Committee concluded that the Bubble-PAPR performed its primary purpose of adequately protecting staff from airborne environmental contaminants.

Fifteen staff contributed to the diary and focus group exercises. Nurses (n=7), Doctors (4) Physiotherapists (2), Advanced Practitioners (1), Speech and Language Therapists (1) representing Emergency Medicine, Critical Care, Orthopaedics and Obstetric specialties generated a list of tasks to be undertaken. One staff member from the 16 invited could not attend the focus group meeting. Staff reported a range of patient-facing activities, including: verbal communication between colleagues and patients; writing; typing; reading notes, computer screens and monitors; manual handling; invasive procedures; emergency resuscitation; airway management; and maintenance of a clean/safe bedside environment. Over the course of the evaluation, staff completed all of the tasks identified by the diary exercise whilst wearing Bubble-PAPR in the clinical environment. Ninety-one staff wore Bubble-PAPR for a median of 45 (IQR 30-90, range 10-150) minutes between 3rd March and 21st December 2021. All relevant staff working in relevant clinical areas were approached until a maximum of six staff had been recruited per shift (the most that the research team could reasonably accommodate per shift), or the recruitment target had been met. No staff who were approached during their clinical shifts were unwilling or unable to trial Bubble-PAPR. There were no Bubble-PAPR-related safety incidents reported during the study. Staff undertook all clinical duties identified by the focus groups and diary card exercise, either in the simulation suite (n=22) or clinical settings (n=22 low-risk, n=25 high-risk, n=22 high-risk with final iteration). Participants predominantly declared as female (69%), and were from a range of clinical and non-clinical roles, Figure S1 (Supplemental). Staff self-reported a range of heights (mean 1.7m [SD 0.1, range 1.5-2.0]), weights (72.4kg [16.0, 47-127]) and body mass indices (25.3 [4.7,16.7-42.9]), Figure S2 (Supplemental). Fifty-two percent of participants reported that they normally wore glasses, with 31% wearing glasses during the evaluation. All participants described at least 6 month's experience with FFP3 face masks on a regular basis ("most shifts"), with a combination of re-useable (typically 3M™ 6000 Series Respirators) and single use (typically 3M™ Aura™ 9330 or equivalent) face masks. No participants described using PAPRs in the six months prior to recruitment. All participants completed all mandatory questionnaire sections.

With pooled data for the primary outcome, "*How comfortable do you feel in your PPE?*" (Likert scale bounded by 1 [very uncomfortable] to 7 [very comfortable]), Bubble-PAPR mean score was 5.64 (SD 1.55) versus usual FFP3 face mask 2.96 (1.44), Figure 2. There was a mean difference of 2.68 (95% CI 2.23-3.14, p<0.001). Secondary outcomes focused on communication and perceived safety. For the question, "*How safe do you feel in your PPE?*", Bubble-PAPR mean score was 6.15 (0.94) vs usual FFP3 face mask 5.43 (0.98); mean difference 0.73 (95% CI 0.45-1.00, p<0.001), Figure 2. Figure 3 demonstrates communication outcomes for all 91 comparisons of Bubble-PAPR versus usual FFP3 face



For peer review only - http://bmjopen.bmj.com/site/about/guidelines.xhtml

masks. All adjusted comparisons were significant ($p<0.001$) in favour of Bubble-PAPR for communicating with both colleagues and patients (Table 2 and Table S5 Supplemental).

Secondary outcomes where a lower Likert response was considered better are presented in Figure S3 (Supplemental). These focussed on whether staff were worried about themselves or others whilst wearing RPE, whether the devices caused pressure or pain or if communication was impaired. Finally, staff were asked if they had to cut short a clinical (or simulated) encounter due to discomfort with their RPE. Again, there was a significant difference in favour of Bubble-PAPR for all metrics (all $p<0.001$, Table 2 and Table S5 Supplemental).

For peer review only



For peer review only - http://bmjopen.bmj.com/site/about/guidelines.xhtml

**Table 2.** Rating scales, summary results and comparisons across the primary outcome questionnaire domains.

| | PPE | Q8 Speak to staff | Q9 Be heard by staff | Q10 Speak to patient | Q11 Be heard by patient | Q14 How safe does it feel | Q17 Comfortable |
|---|---|---|---|---|---|---|---|
| Rating scale | From: | 0 - no confidence | 0 - no confidence | 0 - no confidence | 0 - no confidence | 1 – very unsafe | 1 – very uncomfortable |
| | To: | 10 - fully confident | 10 - fully confident | 10 - fully confident | 10 - fully confident | 7 very safe | 7 very comfortable |
| | | | | | | | |
| RPE type | FFP3 | 5.1 (2.4) [1 - 10] | 4.9 (2.3) [1 - 10] | 4.8 (2.4) [1 - 10] | 4.7 (2.5) [1 - 10] | 5.4 (1.0) [3 – 7] | 3 (1.4) [1 – 6] |
| | Bubble | 7.5 (2.4) [1 - 10] | 7.1 (2.3) [1 - 10] | 7.8 (2.1) [2 - 10] | 7.4 (2.4) [1 - 10] | 6.2 (0.9) [3 – 7] | 5.6 (1.6) [1 – 7] |
| Comparison | Mean difference | 2.38 | 2.16 | 2.99 | 2.7 | 0.73 | 2.68 |
| | 95% CI | 1.66 to 3.11 | 1.45 to 2.88 | 2.36 to 3.62 | 1.97 to 3.43 | 0.45 to 0.99 | 2.23 to 3.14 |
| | | Favours Bubble | Favours Bubble | Favours Bubble | Favours Bubble | Favours Bubble | Favours Bubble |
| | Adjusted p | <0.001 | <0.001 | <0.001 | <0.001 | <0.001 | <0.001 |

For peer review only

During the initial phases, there was no significant difference between staff reporting ease of donning and doffing of Bubble-PAPR and usual PPE (the FFP3 face masks which staff had used for many months at the time of the evaluation). However, pooled results saw staff becoming more familiar with the Bubble, and Bubble-PAPR was rated easier to don and doff when compared with usual FFP3 face masks (adjusted p=0.003 and 0.002 respectively), Table 2 and Figure S4 (Supplemental). One hundred and thirty-two additional free text comments were reviewed and categorised into positive (n=47, 35.6%), negative (67, 50.8%) and neutral (18, 13.6%) comments (Figures S5-7 Supplemental). Most comments focused on the noise of the device, which improved throughout the project as the impellor and motor were made quieter in later design iterations. The categories and nature of comments were as follows: Noise (33 comments [3 neutral, 30 negative]); Comfort (24 comments [20 positive, 2 neutral, 2 negative]); Communication (22 comments [5 positive, 6 neutral, 11 negative]); General (21 comments [17 positive, 2 neutral, 2 negative]); Vision (14 comments [1 positive, 4 neutral, 9 negative]); Wear and fit (10 comments (2 positive, 1 neutral, 7 negative); Stethoscope (5 negative comments); Safety (2 positive comments); Battery (1 negative comment).



For peer review only - http://bmjopen.bmj.com/site/about/guidelines.xhtml

**Discussion**

Our project developed an innovative prototype PAPR explicitly designed for prolonged healthcare use in high-risk clinical environments. Bubble-PAPR achieved its primary purpose of protecting staff by exceeding recognised safety standards for PAPRs, whilst also being rated significantly higher for comfort (the primary outcome), perceived safety, and communication with colleagues and patients (secondary outcomes) than usual FFP3 face masks. Bubble-PAPR was used in all relevant simulated and clinical scenarios identified by detailed staff diary cards, making the results of this study extremely relevant to hospital-based healthcare workers.

Bubble-PAPR was rapidly developed based on the lived experiences of frontline staff during the early stages of the coronavirus pandemic, addressing the unmet needs of reliable, high-quality, universal and available RPE with improved comfort and communication when compared to usual FFP3 face masks. Staff overwhelmingly recognised the importance of facial visualisation when communicating with colleagues and patients. When combined with the improved comfort of wearing a PAPR over usual RPE, participants rated Bubble-PAPR consistently highly across all comparator domains.

This relatively simple evaluation study was preceded by a rapid design and prototyping phase, producing a working prototype within a few weeks. Despite the speed and agility demonstrated by the design team, we adhered to relevant conformity standards for PAPRs, following a tiered evaluation within the governance structure of an approved and regulated research project. Bubble-PAPR was only introduced into higher-risk environments following review by the Trial Safety Committee. This structured approach contrasted with some other rapidly developed or adopted pandemic RPE systems.[7, 13, 14] Whilst the PPE shortages experienced during the pandemic drove many of these innovations and adaptations, we recognised the importance of a methodical approach to design, development and testing of our prototype, both in the laboratory and clinical settings. We recommend others to follow the framework proposed by Duggan et al. for the development of novel medical devices, with regular reviews of safety and useability data within the framework of a robust and transparent clinical trial.[7]

Our study has some limitations. Some of the endpoints were self-reported by participating staff and not independently verified. This included communication between colleagues, and between staff and patients. However, staff were performing their usual clinical duties whilst wearing Bubble-PAPR and any limitations of two-way communication were recognised and reported. The design of Bubble-PAPR addressed many of the issues identified by the same staff who subsequently evaluated the prototype. Whilst our study protocol allowed evaluation only within our Trust owing to the ‘in-house’ manufacturing exemption for testing, it is not unreasonable to expect similar results if our prototype were evaluated elsewhere. Although this may be considered a weakness of the study, many of the shortcomings of the PPE provided to frontline health workers around the world are well described and are essentially the same as those identified in our project.[15, 16] Furthermore, we evaluated Bubble-PAPR against single-use and reusable FFP3 face masks, which could be construed as comparing two different classes of RPE. However, Bubble-PAPR was designed and developed to provide a viable alternative to FFP3 class face masks, in contrast to the more usual healthcare use of PAPRs. Other PAPRs are more complex, more cumbersome (belt-worn fans and hoses), more costly, and typically are selectively available on a limited basis to specific users or groups because of these factors.



For peer review only - http://bmjopen.bmj.com/site/about/guidelines.xhtml

Although a pricing structure is currently unavailable, the simplicity of the design and components (designed with pandemic supply chain limitations in mind) means that Bubble-PAPR is likely to cost around 25-50% of the list price of equivalent PAPRs. Our detailed analysis of work diary cards from various clinical staff ensured that Bubble-PAPR was used for all relevant procedures identified by participating staff in our settings that were undertaken by medical, nursing, healthcare assistant, allied healthcare professional (speech and language therapy, physiotherapy, pharmacy), administrative and domestic staff in the clinical area. Staff were able to undertake their usual duties with this simple, collar-worn PAPR. Limitations of the design include the inability to use a conventional stethoscope (although Bluetooth stethoscopes were used effectively), potential visual distortions if the visor section of the hood became creased, and the residual noise during use (common amongst PAPRs). Although the design is simple, with visual/mechanical indicators instead of electronic indicators or alarms, this did not impact on conformity testing or function. Post-pandemic conformity requirements will vary around the world and future iterations of Bubble-PAPR may need to adapt to meet country-specific requirements. Addressing the actual activities undertaken by specific staff groups, testing safety, performance and the user experience, is unique within published respiratory protective equipment product evaluation studies.[17, 18]

Our study did not directly evaluate the patient experience with staff wearing different RPE. However, the patient experience was reflected in the user specifications identified around communication, and anecdotal feedback was positive from patients, especially around facial visibility and verbal and non-verbal communication. In addition, when contrasted with FFP3 face masks, speech and language therapists reported that demonstrating speech and swallow exercises was suddenly possible with Bubble-PAPR and that the transparent nature of the hood overcame the communication barriers that can be so devastating for those with hearing impairments.[19] Although designed to be potentially recyclable, future work should address the environmental impact of PVC hoods with reusable collars compared to single-use or reusable FFP3 face masks.

For peer review only



For peer review only - http://bmjopen.bmj.com/site/about/guidelines.xhtml

**Conclusions**

Our study has demonstrated that Bubble-PAPR achieved its primary purpose of keeping staff safe from airborne particulate material whilst improving comfort, communication and the user experience when compared with usual FFP3 face masks worn throughout the pandemic. It is likely that the patient experience was also enhanced. Bubble-PAPR has been patented (PCT/GB2021/052147) and subsequently licenced to a UK-based healthcare manufacturer for large-scale manufacture and distribution to frontline NHS and other workers. The pandemic drove unprecedented collaboration between clinicians, academics and industry. Bubble-PAPR is an excellent example of developing a cosmopolitan network across historical, political, and cultural boundaries that could become a key feature of future system resilience.

For peer review only


For peer review only - http://bmjopen.bmj.com/site/about/guidelines.xhtml

**Acknowledgements**

We are grateful to our funders (detailed below) for supporting this project and the staff who participated in the evaluation. In addition, we are indebted to the designers, engineers and staff who gave their time freely during the early stages of the coronavirus pandemic to work tirelessly on designing, building, testing and refining Bubble-PAPR. Specifically:

Patrick Hall, Designing Science Ltd (www.designingscience.co.uk)
Andrew Spragg, Industrial design consultant
Andrew Forbes, XK Design Ltd
James Corden, Manchester University Hospital NHS Foundation Trust Innovation Team
Nick Duggan, Innovation Consultant, Zuas (www.zuas.io)
GAMA Healthcare Ltd, Hemel Hempstead, UK (www.gamahealthcare.com)

**Conflict of interests**

Manchester University NHS Foundation Trust, the University of Manchester and Designing Science Ltd have agreed commercial terms to license Bubble-PAPR for manufacture and development.

**Funding**

This project was supported by unrestricted grants and funding from:

- Engineering and Physical Sciences Research Council (EPSRC) Impact Acceleration Account 302
- Oxford Road Corridor (grant award number N/A)
- Health Innovation Manchester 'Momentum' special projects fund 2021 (grant award number N/A)
- Acute ICU Charitable Research Fund, Manchester University NHS Foundation Trust (grant award number N/A)
- Manchester University NHS Foundation Trust (grant award number N/A)

**Data availability statement**

Due to the commercial sensitivity of the intellectual property licensed at the conclusion of this project, the full dataset is not publicly available. However, the corresponding author will consider requests to disclose the dataset on an individual basis if necessary.

**Trial registration**

Research Ethical and Health Research Authority approval (IRAS ID:288493, REC Ref:21/WA/0018). Manchester University NHS Foundation Trust sponsored the study, acting as the manufacturer of this in-house prototype device. The study protocol, analysis plan and recruitment metrics were registered and reported at ClinicalTrials.gov (NCT04681365).



For peer review only - http://bmjopen.bmj.com/site/about/guidelines.xhtml

### Author contributions

All authors critically revised the manuscript for important intellectual content and approved the final manuscript. BAM attests that all listed authors meet authorship criteria and that no others meeting the criteria have been omitted.

**BAM:** conception and design, collection, analysis and interpretation of data, drafting and revision of the manuscript, and final approval of the version to be published. Participant recruitment.

**CS:** qualitative work package conception and design, analysis and interpretation of data. Participant recruitment. Drafting and revision of the final manuscript, and final approval of the version to be published.

**AG:** qualitative work package design, analysis and interpretation of data. Drafting and revision of the final manuscript, and final approval of the version to be published.

**RC:** design, collection, and interpretation of data, drafting and revision of the manuscript, and final approval of the version to be published.

**JL:** design, collection, and interpretation of data, drafting and revision of the manuscript, and final approval of the version to be published.

**PGA:** design, collection, and interpretation of data, drafting and revision of the manuscript, and final approval of the version to be published.

**GC:** design, collection, and interpretation of data, drafting and revision of the manuscript, and final approval of the version to be published. Manufacturing and engineering lead.

**Provenance and peer review**
Not commissioned; externally peer reviewed.

**Word count of main document:** 3111

### Ethics approval

Research Ethical and Health Research Authority approval (IRAS ID:288493, REC Ref:21/WA/0018) was granted from Wales REC5 on 27th January 2021. The study was sponsored by Manchester University NHS Foundation Trust, who acted as the manufacturer of this in-house prototype device. The study protocol, analysis plan and recruitment metrics were registered and reported at ClinicalTrials.gov (NCT04681365).

For peer review only



For peer review only - http://bmjopen.bmj.com/site/about/guidelines.xhtml

**Table and Figure legends**

**Table 1.** Classification of particulate filters, with a worked example and fit testing.

**Table 2.** Rating scales, summary results and comparisons across the questionnaire domains.

**Table S1. (Supplemental)** Proposed framework for the safer adoption of a MacGyvered device. Adapted from Turner and colleagues.[7]

**Table S2. (Supplemental)** Lab-based testing of the Bubble PAPR prior to clinical evaluation.

**Table S3. (Supplemental).** Pilot data. Q14 & Q17 are Likert Scale items (rated 1-7) and Q8-11 are Visual Analogue Scale items (rated 0-100).

**Table S4. (Supplemental)** Fit testing data from the first 10 participants.

**Figure 1.** Bubble-PAPR comprises a medical-grade foam neck collar and a separate PVC hood. The universal fit collar draws air in through a filter via an impellor powered by an external battery. The collar has a mechanical low flow indicator and can be cleaned and reused by different users. The semi-rigid hood is pulled over the collar before donning and is secured by integrated straps.

**Figure 2.** Reported comfort (primary) and safety (secondary) outcomes for Bubble-PAPR vs usual FFP3 face masks.

**Figure 3.** Secondary communication outcomes where a higher Likert scale response was considered better.

**Figure S1. (Supplemental)** Participant job roles.

**Figure S2. (Supplemental)** Self-reported weight, height and BMI of staff participants.

**Figure S3. (Supplemental)** Secondary outcomes where a lower Likert scale response was considered better.

**Figure S4. (Supplemental)** Ease of donning and doffing of Respiratory Protective Equipment.

**Figure S5. (Supplemental).** Word clouds from the free text feedback. Negative comments (all categories).

**Figure S6 (Supplemental).** Word clouds from the free text feedback. Neutral comments (all categories).

**Figure S7 (Supplemental).** Word clouds from the free text feedback. Positive comments (all categories).



For peer review only - http://bmjopen.bmj.com/site/about/guidelines.xhtml
For peer review only

## References


1. Fadela Chaib, World Health Organisation. Shortage of personal protective equipment endangering health workers worldwide. 3rd March 2020. Available from www.who.int/news/item/03-03-2020-shortage-of-personal-protective-equipment-endangering-health-workers-worldwide [Accessed 5th May 2020].

2. Hignett S, Welsh R, Banerjee J. Human factors issues of working in personal protective equipment during the COVID-19 pandemic. *Anaesthesia* 2021; 76: 134-135.

3. Cook TM. Personal protective equipment during the coronavirus disease (COVID) 2019 pandemic - a narrative review. *Anaesthesia* 2020; 75: 920-927.

4. British Standard BS EN12941 Respiratory protective devices - Powered filtering devices incorporating a helmet or a hood - Requirements, testing, marking (British Standard). 1999. Available from https://standards.globalspec.com/ [Accessed 5th April 2020].

5. Rizan C, Reed M, Bhutta MF. Environmental impact of personal protective equipment distributed for use by health and social care services in England in the first six months of the COVID-19 pandemic. *Journal of the Royal Society of Medicine* 2021; 114: 250-263.

6. Foo CC, Goon AT, Leow YH, Goh CL. Adverse skin reactions to personal protective equipment against severe acute respiratory syndrome--a descriptive study in Singapore. *Contact Dermatitis* 2006; 55: 291-294.

7. Turner MC, Duggan LV, Glezerson BA, Marshall SD. Thinking outside the (acrylic) box: a framework for the local use of custom-made medical devices. *Anaesthesia* 2020; 75: 1566-1569.

8. Gould CL, Alexander PDG, Allen CN, McGrath BA, Shelton CL. Protecting staff and patients during airway management in the COVID-19 pandemic: are intubation boxes safe. *British Journal of Anaesthesia* 2020; 125: e292-e293.

9. Shelton C, El-Boghdadly K, Appleby JB. The 'haves' and 'have-nots' of personal protective equipment during the COVID-19 pandemic: the ethics of emerging inequalities amongst healthcare workers. *Journal of Medical Ethics* 2021; medethics-2021-107501.

10. Great Britain Health and Safety Executive. Guidance on Respiratory Protective Equipment (RPE) Fit Testing: (Rev1). March 2019. Available from https://www.hse.gov.uk/pubns/indg479.htm [Accessed 5th April 2020].

11. Davis FD. Perceived Usefulness, Perceived Ease of Use, and User Acceptance of Information Technology. *MIS Quarterly* 1989; 13: 319-340.

12. European Agency for Health and Safety at Work. Regulation (EU) 2016/425 on Personal Protective Equipment. 2016. Available from https://osha.europa.eu/en/legislation/directive/regulation-eu-2016425-personal-protective-equipment. [Accessed 5th April 2020].

13. Duggan LV, Marshall SD, Scott J, Brindley PG, Grocott HP. The MacGyver bias and attraction of homemade devices in healthcare. *Canadian Journal of Anesthesia/Journal Canadien d'Anesthésie* 2019; 66: 757-761.

14. Addi RA, Benksim A, Cherkaaoui M. Easybreath Decathlon Mask: An Efficient Personal Protective Equipment (PPE) against COVID-19 in Africa. *Journal of Clinical and Experimental Investigations* 2020; 11: em00738.

15. Kim H, Hegde S, LaFiura C et al. Access to personal protective equipment in exposed healthcare workers and COVID-19 illness, severity, symptoms and duration: a population-based case-control study in six countries. *BMJ Global Health* 2021; 6: e004611.

16. Hoernke K, Djellouli N, Andrews L et al. Frontline healthcare workers' experiences with personal protective equipment during the COVID-19 pandemic in the UK: a rapid qualitative appraisal. *BMJ Open* 2021; 11: e046199.




For peer review only - http://bmjopen.bmj.com/site/about/guidelines.xhtml

17. Galanis P, Vraka I, Fragkou D, Bilali A, Kaitelidou D. Impact of personal protective equipment use on health care workers' physical health during the COVID-19 pandemic: A systematic review and meta-analysis. *American Journal of Infection Control* 2021; 49: 1305-1315.

18. Houghton C, Meskell P, Delaney H et al. Barriers and facilitators to healthcare workers' adherence with infection prevention and control (IPC) guidelines for respiratory infectious diseases: a rapid qualitative evidence synthesis. *Cochrane Database of Systematic Reviews* 2020; 4: CD013582.

19. Poostchi A, Kuet ML, Richardson PS, Patel MK. Covid-19: face masks can be devastating for people with hearing loss but alternatives are available. *BMJ* 2020; 370: m3326.

For peer review only


For peer review only - http://bmjopen.bmj.com/site/about/guidelines.xhtml

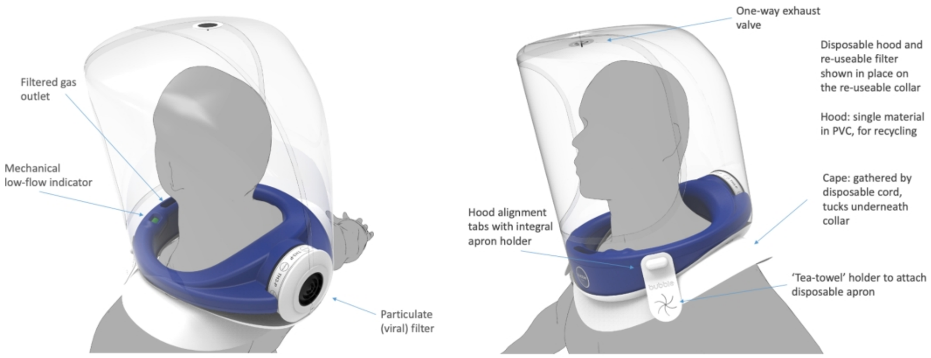


Bubble-PAPR comprises a medical-grade foam neck collar and a separate PVC hood. The universal fit collar draws air in through a filter via an impellor powered by an external battery. The collar has a mechanical low flow indicator and can be cleaned and reused by different users. The semi-rigid hood is pulled over the collar before donning and is secured by integrated straps.

762x283mm (72 x 72 DPI)

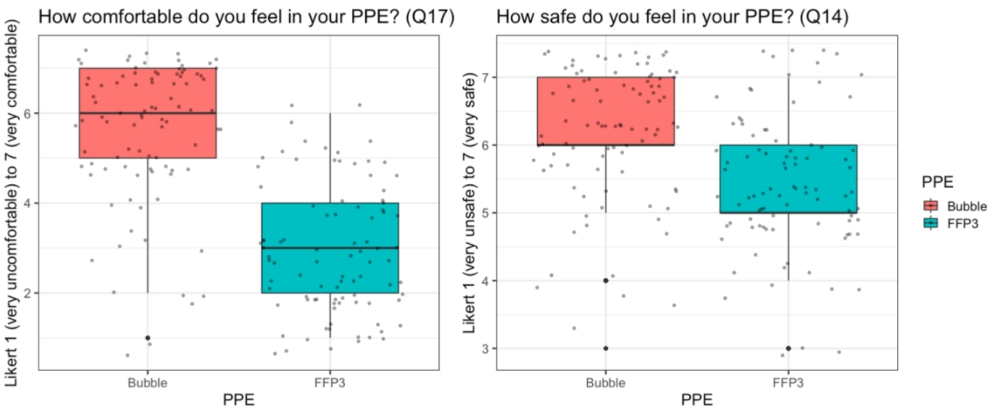


Reported comfort (primary) and safety (secondary) outcomes for Bubble-PAPR vs usual FFP3 face masks.

537x225mm (72 x 72 DPI)

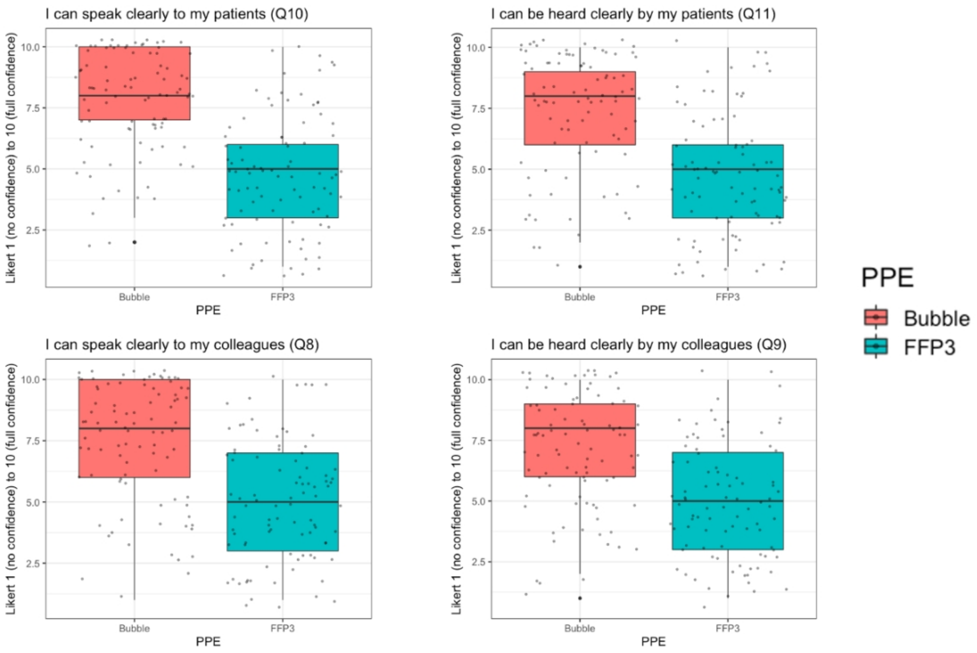


Secondary communication outcomes where a higher Likert scale response was considered better.

446x295mm (72 x 72 DPI)

**Table S1. (Supplemental)** Proposed framework for the safer adoption of a MacGyvered device. Adapted from Turner and colleagues.[7]

1. Define the problem and rule out the suitability of existing solutions
2. List benchmark safety indices for the device
3. Seek broader feedback from all stakeholders on the design's utility and potential pitfalls.
4. Perform laboratory-based and in situ simulations.
5. Introduce into low-risk clinical settings after local due process and patient consent.
6. Introduce into higher-risk clinical settings with a discrete group of trained 'super-users'.
7. Encourage an iterative cycle of feedback, review, re-design and improvement.
8. **Do not:** adopt, publish, endorse or disseminate via social media a MacGyvered device without data to support safety.

For peer review only


For peer review only - http://bmjopen.bmj.com/site/about/guidelines.xhtml

**Table S2. (Supplemental). Lab-based testing of the Bubble PAPR prior to clinical evaluation**

All of the bench tests detailed below were carried out by the Electrical and Biomedical Engineering (EMBE) team based at Wythenshawe Hospital (MFT), the University of Manchester Mechanical Aerospace and Civil Engineering team (UoM) or by INSPEC International, Salford, UK. A PAPR unit was supplied, and the Instructions for Use were followed by the independent tester, with a judgement made if they fulfilled particular requirements. Some requirements are supplemented by the qualitative or quantitative data collected in the questionnaires. Standards used were British Standard EN12941 (BS, 2008) and the European Regulations for Respiratory Protective Equipment EU2016/425 (ER, 2016).

| Relevant section of standard | Standard detail | Test location | Test detail | Results/notes | Pass/Fail |
|---|---|---|---|---|---|
| BS 6.1.1<br>ER 1.3.2 | Suitable resistance to wear and tear | MFT | PAPR units inspected after 1 week of continual use. Images taken before and after. | Opinion. Baseline inspection +/- photograph. Review after 1 week | Pass 14/3/21 |
| | | | | | |
| BS 6.1.4<br>ER 1.2.1.2 | No sharp edges | MFT | Visual and physical inspection<br>Reports from staff evaluation | Opinion. Baseline inspection +/- photograph. Review after 1 week | Pass 14/3/21 |
| | | | | | |
| BS 6.3.2 | Fits a range of head sizes | MFT | Ten participants will undergo fit testing.<br>These participants will have height, weight and head circumference measured as part of this standard process. | Fit test data shared with EBME team. All fit factors >500 as per BS EN 12941 standard. | Pass 25/2/21 |
| | | | | | |
| BS 6.3.3.1 | Does not distort vision | MFT | The optical area appears transparent. | Inspection by EBME team | Pass 25/2/21 |
| BS 6.3.3.2 | Permits appropriate field of view | MFT | Reports from staff evaluation | Review of results from initial staff evaluations | Pass 14/3/21 |



For peer review only - http://bmjopen.bmj.com/site/about/guidelines.xhtml

| ER2.3 | | | | | |
|---|---|---|---|---|---|
| Relevant section of standard | Standard detail | Test location | Test detail | Results/notes | Pass/Fail |
| BS 6.9 | Cannot reverse airflow | MFT | Normal use.<br>Simulate blocked filter and blocked air duct.<br>Flowmeter. | | Pass 14/3/21 |
| BS 6.9 | Battery safe – protection from short circuit | MFT | EBME check on battery packs (suitable for purpose / recommended packs) | As per manufacturer documentation. Will not be separately tested. | Pass 25/2/21 |
| | | | | | |
| ER2.12 | Appropriate markings | MFT | Yoke manufactured with section for appropriate sticker | | Pass 25/2/21 |
| | | | | | |
| ER3.10.1 | Appropriate training provided | MFT | Instructions for use provided.<br>Training videos provided. | Instructions for Use provided to EBME.<br>Training videos available. | Pass 25/2/21 |
| | | | | | |
| No specific clause | Cleanable | MFT | Specification of foam material for yoke details cleaning methods, durability and material fatigue. | Specification provided to EBME. | Pass 25/2/21 |
| BS 6.16 | Mass shall not exceed 5kg. A maximum of 1.5kg shall be carried on the head. | MFT | Weigh the assembled Bubble PAPR | Assembled Bubble PAPR Weight = 1.4kg | Pass 14/3/21 |


For peer review only - http://bmjopen.bmj.com/site/about/guidelines.xhtml

| Relevant section of standard | Standard detail | Test location | Test detail | Results/notes | Pass/Fail |
|---|---|---|---|---|---|
| BS 6.1.3 | Repeated cleaning and disinfection – does not deteriorate | MFT | PAPR units are inspected after 1 week of continual use. Images taken before and after.<br><br>Specification of foam material for yoke details cleaning methods, durability and material fatigue. | Spec sheet and review after 1 week of use. | Pass 14/3/21 |
| | | | | | |
| BS 6.4 | Ingress protection test for 10 test subjects – using either or both methods (bitter and/or particulometer). | MFT | 10 users will undergo fit testing with particulometer. | Fit test data shared with EBME team. All fit factors >500 as per BS EN 12941 standard. | Pass 25/2/21 |
| ER 3.10.2 | Appropriate protection to eye and skin irritants | MFT | | | |


For peer review only - http://bmjopen.bmj.com/site/about/guidelines.xhtml

| Relevant section of standard | Standard detail | Test location | Test detail | Results/notes | Pass/Fail |
|---|---|---|---|---|---|
| BS 6.2 (6.4) | Repeated after hood/yoke is conditioned at maximum specified temperature and humidity.<br><br>Complete unit is stored for 72 +/- 1 hours at the upper extreme of temperature and humidity specified by the manufacturer. Unit is allowed to return to ambient conditions for 4 hours, then stored for 72 +/- 1 hours at the lower extreme of temperature and humidity. | MFT | Unit is subjected to particulometer fit testing after appropriate temperature conditioning. | *Conditioning beyond use on the ICU should not be required for the MFT in-house evaluation*<br><br>All fit tests took place on the Acute ICU at Wythenshawe | Pass 25/2/21 |
| | | | | | |
| BS 6.5 | Positive pressure inside the hood remains below 5mbar | MFT | 1 user and 1 dummy test head setup. Pressure measurement inside hood during regular use. | Measured pressure below 5mBar | Pass 14/3/21 |
| BS 6.6.2 | Exceeds manufacturer's minimum specified airflow for a period of at least 4 hours.<br><br>(The UK/EU regs do not specify a minimal flow. US regulations do, but this is not immediately relevant) | MFT, INSPEC | Test head and flow meter.<br><br>*Note the flow meter arrangement is slightly complex as the measurement itself can interfere with flow.* | Breathe Safety report reviewed. | Pass 25/2/21 |


For peer review only - http://bmjopen.bmj.com/site/about/guidelines.xhtml

| Relevant section of standard | Standard detail | Test location | Test detail | Results/notes | Pass/Fail |
|---|---|---|---|---|---|
| BS 6.7 | Check function of minimum airflow indicator. | MFT, INSPEC | Apply different flow rates to the yoke measured by external flow meter and evaluate performance of the minimum airflow indicator in units. | Breathe Safety report reviewed. | Pass 25/2/21 |
| | | | | | |
| EN 143<br>BS 6.8 | Clogging of filter | MFT, INSPEC | Flow through filter and yoke tested after 4 hours of use with an external flow meter. | Breathe Safety report reviewed. | Pass 25/2/21 |
| | | | | | |
| 6.13 | The carbon dioxide content of the inhalation air (dead space inside the hood) shall not exceed an average of 1% by volume.<br><br>There is a specific test-rig setup for this. A physiological surrogate model should provide adequate assurance that the $CO_2$ content inside the hood is <1% during normal use. | MFT, INSPEC | Oxygen and carbon dioxide (gas analysis) inside the hood measured as partial pressures and/or percentages using MFT EBME equipment. | Breathe Safety report reviewed. | Pass 25/2/21 |


For peer review only - http://bmjopen.bmj.com/site/about/guidelines.xhtml

| Relevant section of standard | Standard detail | Test location | Test detail | Results/notes | Pass/Fail |
|---|---|---|---|---|---|
| BS 6.15 | Exhalation means (valve) maximum flowrate and safe operation:<br><br>Hood performs adequately during normal use. Specifically; exhalation means:<br>• functions and can be replaced (new hood)<br>• functions in orientations encountered during normal use<br>• is protected against dirt and mechanical damage | MFT | Test subject wears hood.<br><br>Eventually, this section is supplemented with reports from staff evaluation. | Review of initial feedback from users in sim setting | Pass 14/3/21 |
| BS 6.15 | Exhalation means (valve) maximum flowrate and safe operation:<br><br>Continuous flow rate of 300 +/- 15 L/min is applied for a period of 60+/- 6 secs. | MFT | Flow generator (ventilator) and flow meter.<br><br>Visual inspection of exhalation valve. | Opinion of EBME team. The flow generator works as described with the test flowmeter supplied. Exhalation Valve inspection pass. | Pass 14/3/21 |

For peer review only

**Table S3. (Supplemental). Pilot data. Q14 & Q17 are Likert Scale items (rated 1-7) and Q8-11 are Visual Analogue Scale items (rated 0-100).**

| | Current PPE | | | | | | BUBBLE-PAPR | | | | | |
|---|---|---|---|---|---|---|---|---|---|---|---|---|
| Participant | Q14 Safe to wear | Q17 Comfort to wear | Q8 Speak clearly to colleagues | Q9 Be heard by colleagues | Q10 Speak clearly to patients | Q11 Be heard by patients | Q14 Safe to wear | Q17 Comfort to wear | Q8 Speak clearly to colleagues | Q9 Be heard by colleagues | Q10 Speak clearly to patients | Q11 Be heard by patients |
| 1 | 3 | 3 | 10 | 20 | 0 | 0 | 4 | 5 | 75 | 75 | 80 | 80 |
| 2 | 4 | 2 | 15 | 20 | 10 | 30 | 7 | 5 | 85 | 80 | 60 | 70 |
| 3 | 4 | 4 | 20 | 20 | 30 | 30 | 5 | 6 | 90 | 90 | 90 | 90 |
| 4 | 5 | 2 | 25 | 25 | 10 | 10 | 6 | 6 | 95 | 95 | 95 | 85 |
| 5 | 5 | 4 | 33 | 40 | 25 | 25 | 6 | 5 | 75 | 70 | 85 | 90 |
| 6 | 6 | 3 | 30 | 25 | 30 | 30 | 6 | 5 | 65 | 70 | 55 | 66 |
| 7 | 7 | 2 | 20 | 30 | 25 | 25 | 7 | 5 | 70 | 70 | 65 | 65 |
| 8 | 4 | 3 | 45 | 50 | 20 | 30 | 7 | 6 | 90 | 95 | 90 | 90 |
| Mean | 4.8 | 2.9 | 24.8 | 28.8 | 18.8 | 22.5 | 6.0 | 5.4 | 80.6 | 80.6 | 77.5 | 79.5 |
| SD | 1.3 | 0.8 | 11.1 | 10.9 | 10.9 | 11.3 | 1.1 | 0.5 | 10.8 | 11.2 | 15.4 | 11.0 |

McGrath et al. Supplemental Tables

For peer review only - http://bmjopen.bmj.com/site/about/guidelines.xhtml

**Table S4. (Supplemental) Fit testing data from the first 10 participants.**

Test protocol HSE INDG 479. Pass level set at a fit factor of 500.

| Subject | Self-reported height | Self-reported weight | BMI | Normal Breathing 1 | Deep Breathing | Head Side to Side | Head Up and Down | Talking | Bending at the waist | Normal Breathing 2 | Overall Fit Factor |
|---|---|---|---|---|---|---|---|---|---|---|---|
| **1** | 1.86 | 74 | 21.4 | 79705 | 43647 | 125478 | 11125 | 107899 | 1339 | 76152 | **7757** |
| **2** | 1.95 | 75 | 19.7 | 53792 | 52343 | 59440 | 51673 | 52733 | 50433 | 45961 | **52075** |
| **3** | NR | NR | NR | 38867 | 36699 | 37097 | 41474 | 39500 | 36884 | 37465 | **38217** |
| **4** | 1.82 | 65 | 19.6 | 17745 | 6622 | 3149 | 5028 | 31996 | 30520 | 31326 | **8539** |
| **5** | 1.65 | 55 | 20.2 | 24945 | 25215 | 3885 | 8097 | 28877 | 29107 | 24393 | **12268** |
| **6** | 1.67 | 58 | 20.8 | 24617 | 25608 | 25581 | 25225 | 20107 | 1924 | 23517 | **9088** |
| **7** | 1.52 | 47 | 20.3 | 28747 | 30700 | 33203 | 15275 | 31671 | 8327 | 26041 | **19829** |
| **8** | 1.65 | 65 | 23.9 | 27282 | 31318 | 34900 | 9697 | 29093 | 3514 | 25770 | **12544** |
| **9** | 1.83 | 115 | 34.3 | 11182 | 1123 | 11028 | 24524 | 24692 | 2537 | 23704 | **4408** |
| **10** | 1.59 | 75 | 29.7 | 25760 | 3419 | 16125 | 2433 | 25523 | 1552 | 26154 | **4588** |
| | | | | | | | | | | | |
| **Mean values** | | | | **33264** | **25669** | **34989** | **19455** | **39209** | **16614** | **34048** | **16931** |


For peer review only - http://bmjopen.bmj.com/site/about/guidelines.xhtml

**Table S5.** Rating scales, summary results and comparisons across all of the questionnaire domains.

| | PPE | Q1 Confidence in donning | Q2 Confidence in donning without dislodging other PPE | Q3 Wear with glasses/goggles | Q4 Protect yourself from respiratory infection | Q5 Protect patient from infection from you | Q6 Safely care for your patient | Q7 Safely roll patient | Q8 Speak to staff | Q9 Be heard by staff | Q10 Speak to patient | Q11 Be heard by patient | Q12 Doff safely | Q13 Doff without dislodging glasses |
|---|---|---|---|---|---|---|---|---|---|---|---|---|---|---|
| Rating scale | From: | 0 - no confidence | 0 - no confidence | 0 - no confidence | 0 - no confidence | 0 - no confidence | 0 - no confidence | 0 - no confidence | 0 - no confidence | 0 - no confidence | 0 - no confidence | 0 - no confidence | 0 - no confidence | 0 - no confidence |
| | To: | 10 - fully confident | 10 - fully confident | 10 - fully confident | 10 - fully confident | 10 - fully confident | 10 - fully confident | 10 - fully confident | 10 - fully confident | 10 - fully confident | 10 - fully confident | 10 - fully confident | 10 - fully confident | 10 - fully confident |
| | | | | | | | | | | | | | | |
| RPE type | FFP3 | 8.9 (1.4) [3 - 10] | 8.3 (2) [2 - 10] | 6.9 (2.6) [2 - 10] | 8.2 (1.6) [4 - 10] | 8.2 (1.7) [2 - 10] | 8.4 (1.4) [5 - 10] | 8.2 (1.8) [2 - 10] | 5.1 (2.4) [1 - 10] | 4.9 (2.3) [1 - 10] | 4.8 (2.4) [1 - 10] | 4.7 (2.5) [1 - 10] | 8.1 (1.9) [2 - 10] | 6.2 (2.5) [1 - 10] |
| | Bubble | 7.4 (1.8) [3 - 10] | 7.7 (1.8) [2 - 10] | 7.6 (1.9) [3 - 10] | 8.6 (1.6) [3 - 10] | 8.5 (1.8) [2 - 10] | 8.0 (2) [2 - 10] | 7.8 (2.2) [2 - 10] | 7.5 (2.4) [1 - 10] | 7.1 (2.3) [1 - 10] | 7.8 (2.1) [2 - 10] | 7.4 (2.4) [1 - 10] | 8.0 (1.8) [2 - 10] | 7.8 (1.9) [2 - 10] |
| Comparison | Mean difference | -1.48 | -0.55 | 0.7 | 0.43 | 0.3 | -0.42 | -0.42 | 2.38 | 2.16 | 2.99 | 2.7 | -0.1 | 1.66 |
| | 95% CI | -1.9 to -0.99 | -1.12 to 0.02 | -0.00 to 1.40 | -0.04 to 0.89 | -0.18 to 0.78 | -0.91 to 0.07 | -0.98 to 0.15 | 1.66 to 3.11 | 1.45 to 2.88 | 2.36 to 3.62 | 1.97 to 3.43 | -0.63 to 0.43 | 0.98 to 2.34 |
| | | Favours FFP3 | No difference | Favours Bubble | No difference | No difference | No difference | No difference | Favours Bubble | Favours Bubble | Favours Bubble | Favours Bubble | No difference | Favours Bubble |
| | Adjusted p | <0.001 | 0.058 | 0.049 | 0.070 | 0.217 | 0.092 | 0.144 | <0.001 | <0.001 | <0.001 | <0.001 | 0.711 | <0.001 |

| | PPE | Q14 How safe does it feel | Q15 Worried about own health | Q16 Worried others health | Q17 Comfortable | Q18 Don ease | Q19 Doff ease | Q20a Restricted communication | Q20b Vision distorted with Bubble | Q20c Read monitors, computers, and notes with Bubble | Q21 Pressure marks on head/face | Q22 Pain on head/face | Q23 Leave clinical area early due to RPE |
|---|---|---|---|---|---|---|---|---|---|---|---|---|---|
| Rating scale | From: | 1 - very unsafe | 1 - not worried at all | 1 - not worried at all | 1 - very uncomfortable | 1 - not at all easy | 1 - not at all easy | 1 - not at all restricted | 1 - not at all affected | 1 - clear at all times | 1 - never | 1 - never | 1 - never |
| | To: | 7 very safe | 7 - very worried | 7 - very worried | 7 - very comfortable | 7 - very easy | 7 - very easy | 7 - very restricted | 7 - very affected | 7 - not clear at all | 7 - always | 7 - always | 7 - always |
| | | | | | | | | | | | | | |
| RPE type | FFP3 | 5.4 (1.0) [3 - 7] | 3.2 (1.5) [1 - 7] | 3.2 (1.5) [1 - 7] | 3 (1.4) [1 - 6] | 4.9 (1.3) [2 - 7] | 5.1 (1.3) [2 - 7] | 5.4 (1.4) [1 - 7] | - | - | 5.8 (1.4) [1 - 7] | 5.3 (1.4) [1 - 7] | 3.6 (1.6) [1 - 7] |
| | Bubble | 6.2 (0.9) [3 - 7] | 2.3 (1.6) [1 - 7] | 2.3 (1.7) [1 - 7] | 5.6 (1.6) [1 - 7] | 5.5 (1.4) [2 - 7] | 5.7 (1.2) [2 - 7] | 3.9 (1.7) [1 - 7] | 3.2 (1.9) [1 - 7] | 5.6 (1.5) [2 - 7] | 1.3 (0.8) [1 - 6] | 1.4 (0.9) [1 - 6] | 1.5 (1) [1 - 6] |
| Comparison | Mean difference | 0.73 | -0.92 | -0.93 | 2.68 | 0.62 | 0.63 | -1.49 | - | - | -4.54 | -3.99 | -2.13 |
| | 95% CI | 0.45 to 0.99 | -1.36 to -0.49 | -1.36 to -0.48 | 2.23 to 3.14 | 0.21 to 1.02 | 0.26 to 0.99 | -1.95 to -1.04 | - | - | -4.90 to -4.17 | -4.35 to -3.63 | -2.51 to - 1.75 |
| | | Favours Bubble | Favours Bubble | Favours Bubble | Favours Bubble | Favours Bubble | Favours Bubble | Favours Bubble | No comparator | No comparator | Favours Bubble | Favours Bubble | Favours Bubble |
| | Adjusted p | <0.001 | <0.001 | <0.001 | <0.001 | 0.003 | 0.002 | <0.001 | - | - | <0.001 | <0.001 | <0.001 |

For peer review only

Figure S1. (Supplemental) Participant job roles.

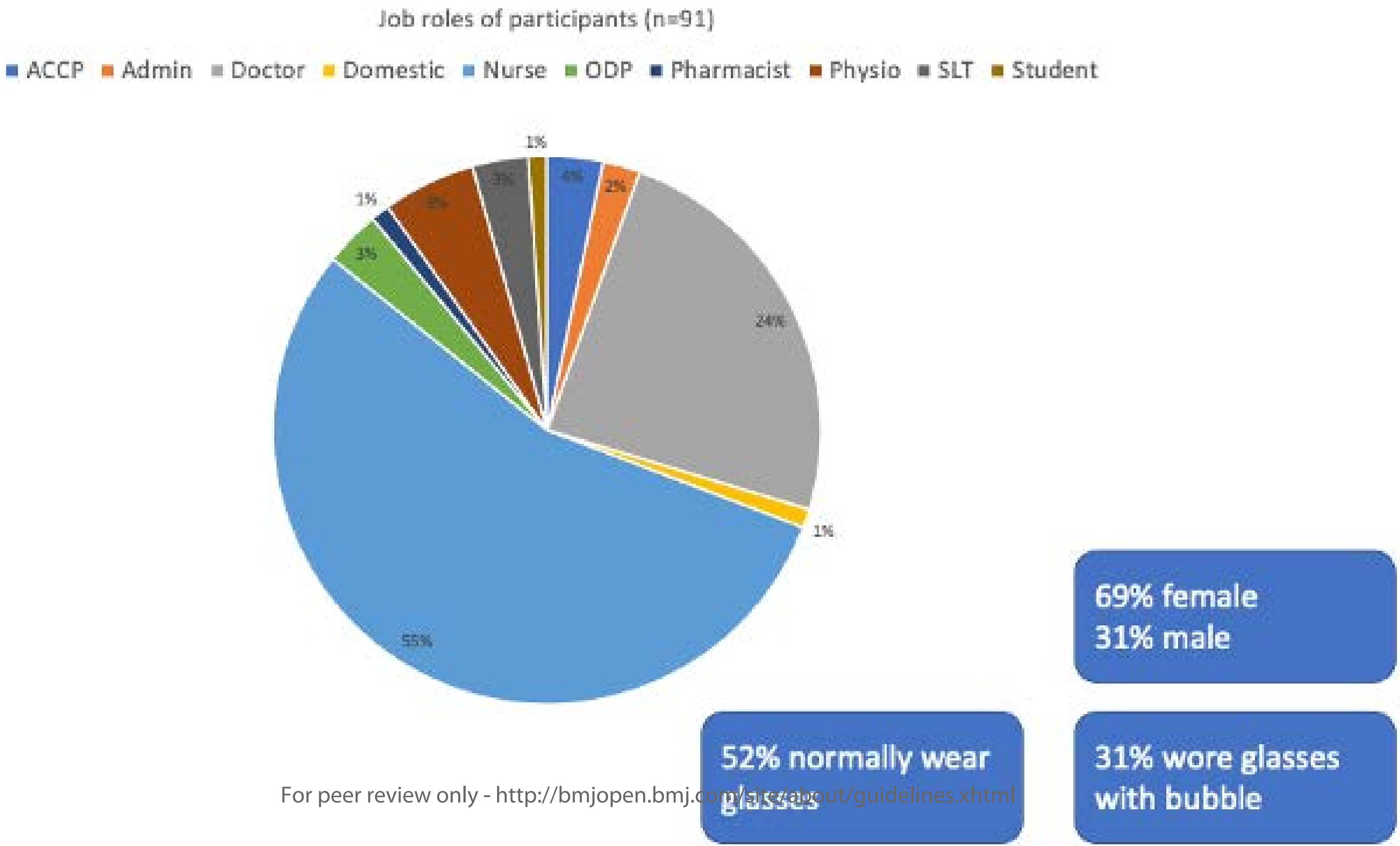

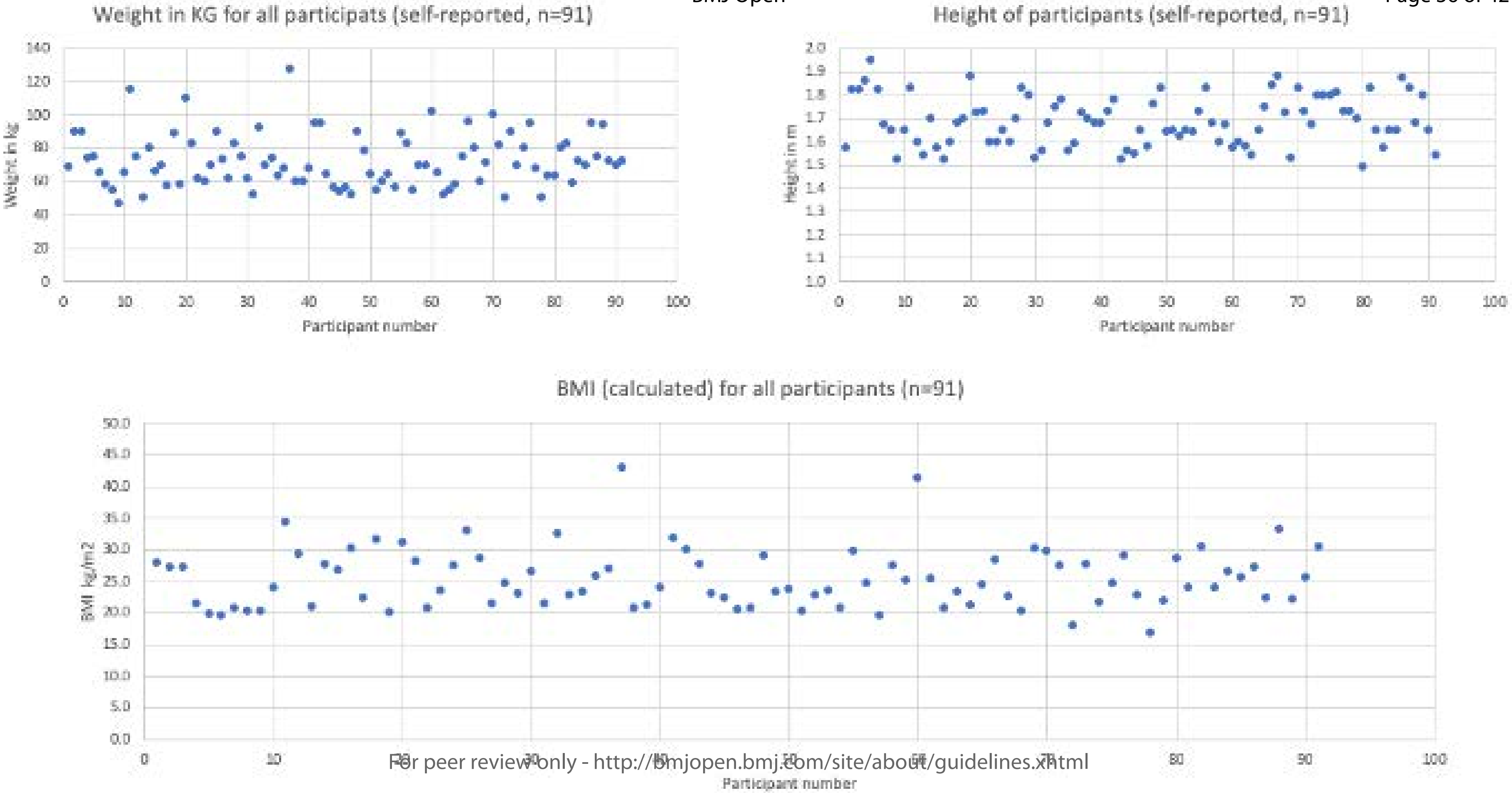




Figure S2. (Supplemental) Self-reported weight, height and BMI of staff participants.

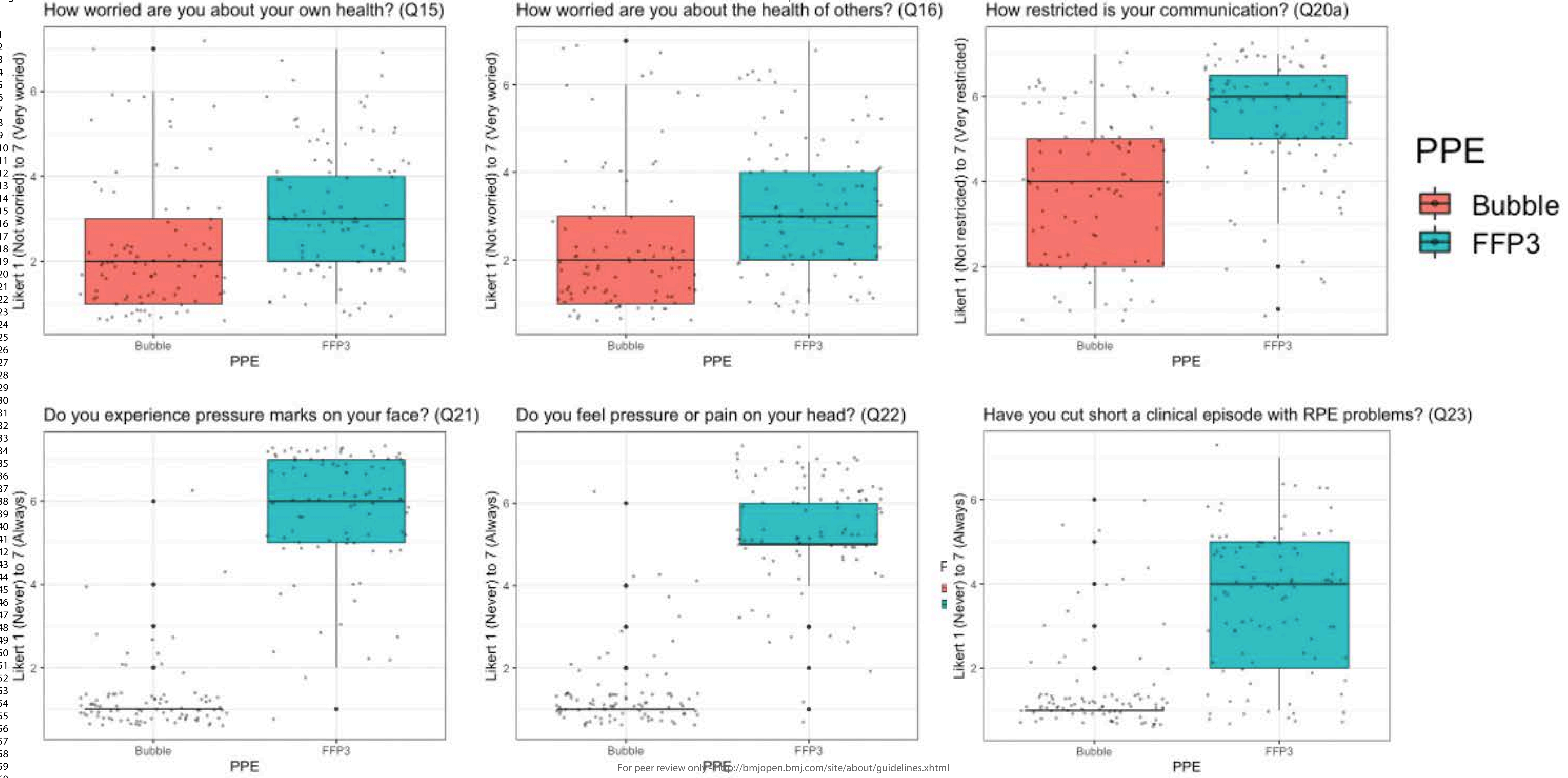




Figure S3. (Supplemental) Secondary outcomes where a lower Likert scale response was considered better.

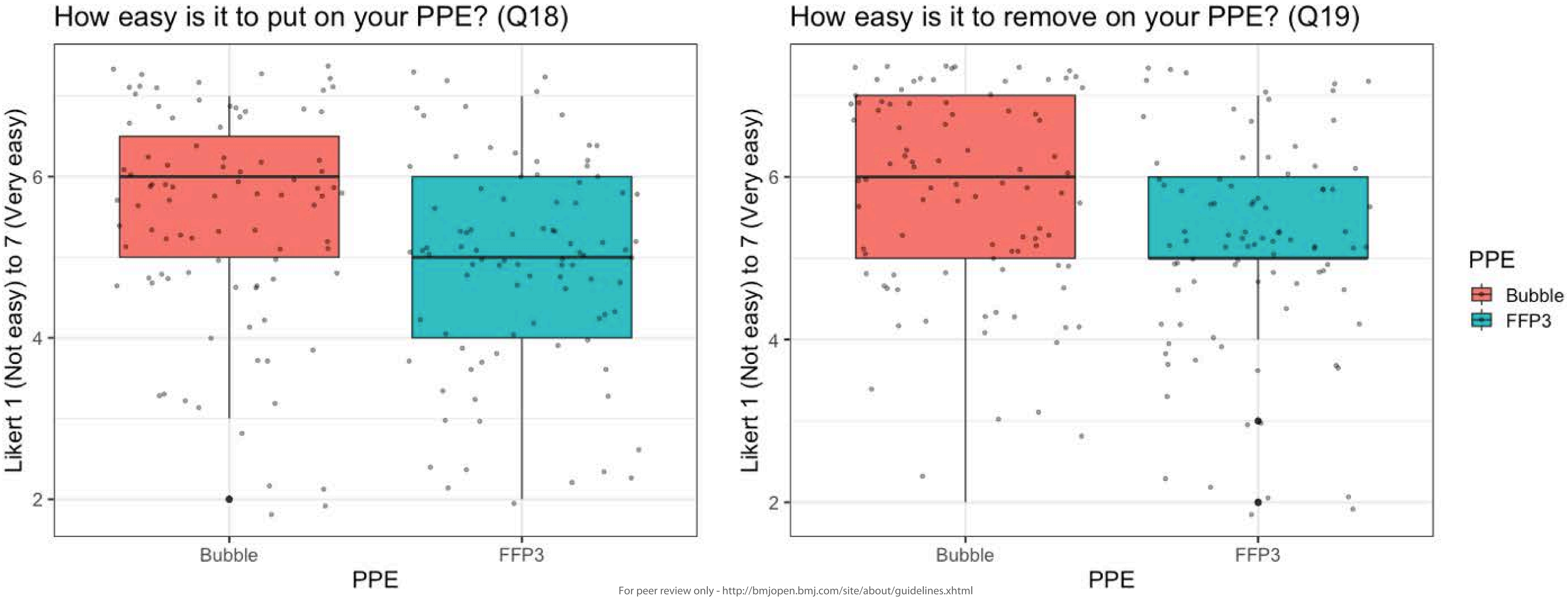


For peer review only - http://bmjopen.bmj.com/site/about/guidelines.xhtml

Figure S4. (Supplemental) Ease of donning and doffing of Respiratory Protective Equipment.

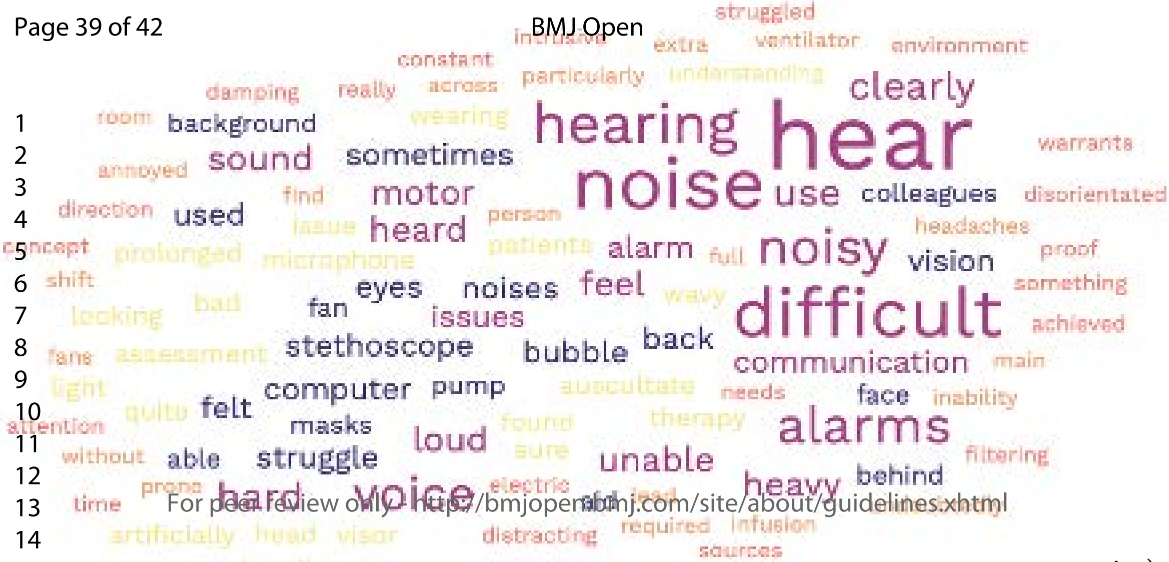




Figure S5. (Supplemental). Word clouds from the free text feedback. Negative comments (all categories).

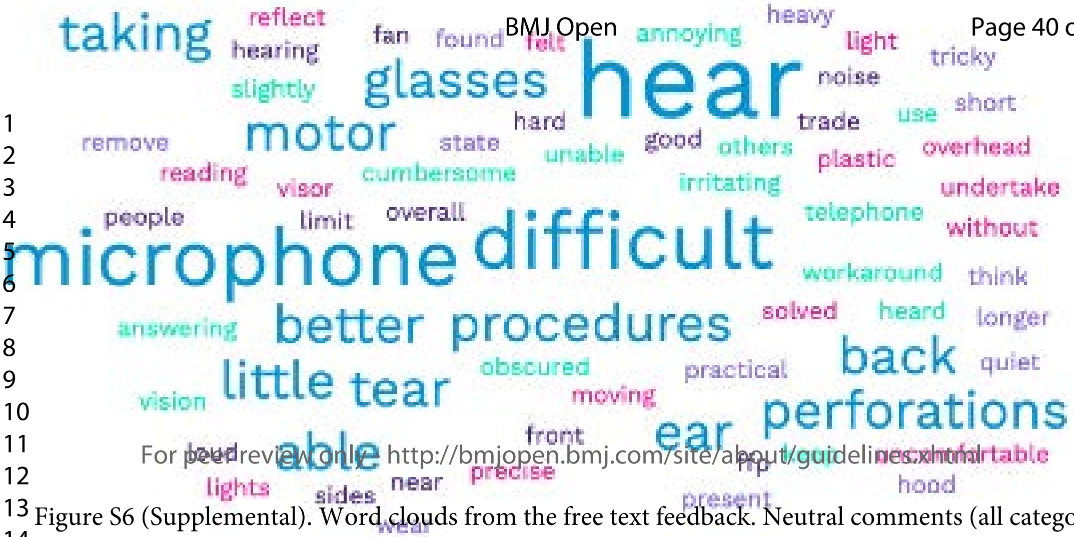

Figure S6 (Supplemental). Word clouds from the free text feedback. Neutral comments (all categories).

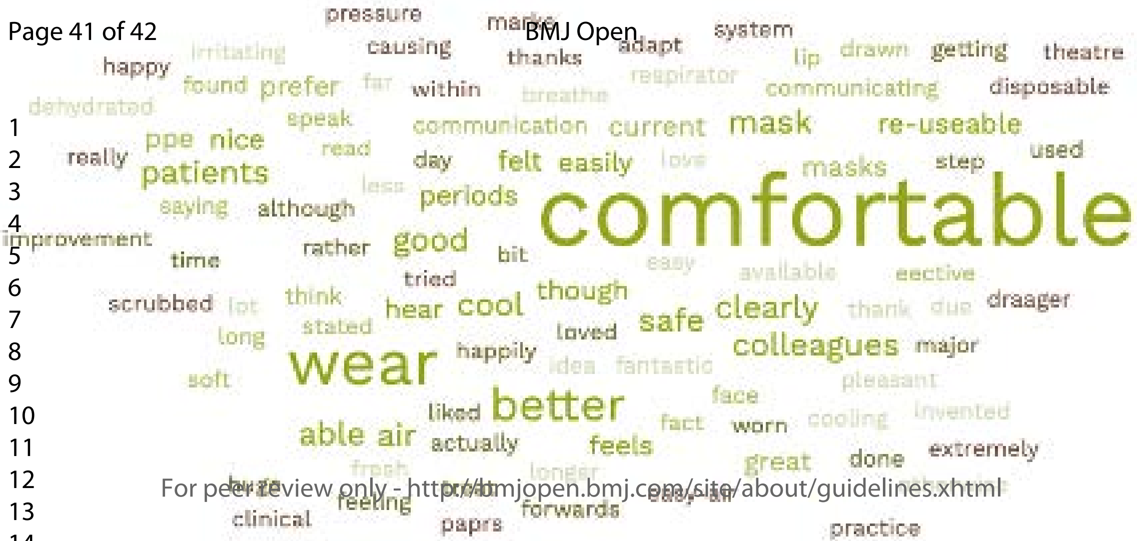


For peer review only - http://bmjopen.bmj.com/site/about/guidelines.xhtml

Figure S7 (Supplemental). Word clouds from the free text feedback. Positive comments (all categories).

STROBE Statement—checklist of items that should be included in reports of observational studies

| | Item No | Recommendation | Page No |
|---|---|---|---|
| **Title and abstract** | 1 | (*a*) Indicate the study's design with a commonly used term in the title or the abstract | 1 |
| | | (*b*) Provide in the abstract an informative and balanced summary of what was done and what was found | 2 |
| **Introduction** | | | |
| Background/rationale | 2 | Explain the scientific background and rationale for the investigation being reported | 3 |
| Objectives | 3 | State specific objectives, including any prespecified hypotheses | 4 |
| **Methods** | | | |
| Study design | 4 | Present key elements of study design early in the paper | 5 |
| Setting | 5 | Describe the setting, locations, and relevant dates, including periods of recruitment, exposure, follow-up, and data collection | 5 |
| Participants | 6 | (*a*) *Cohort study*—Give the eligibility criteria, and the sources and methods of selection of participants. Describe methods of follow-up<br>*Case-control study*—Give the eligibility criteria, and the sources and methods of case ascertainment and control selection. Give the rationale for the choice of cases and controls<br>*Cross-sectional study*—Give the eligibility criteria, and the sources and methods of selection of participants | 6 |
| | | (*b*) *Cohort study*—For matched studies, give matching criteria and number of exposed and unexposed<br>*Case-control study*—For matched studies, give matching criteria and the number of controls per case | |
| Variables | 7 | Clearly define all outcomes, exposures, predictors, potential confounders, and effect modifiers. Give diagnostic criteria, if applicable | 6 and Supplemental Material |
| Data sources/ measurement | 8* | For each variable of interest, give sources of data and details of methods of assessment (measurement). Describe comparability of assessment methods if there is more than one group | 6 |
| Bias | 9 | Describe any efforts to address potential sources of bias | 5 |
| Study size | 10 | Explain how the study size was arrived at | 6 |
| Quantitative variables | 11 | Explain how quantitative variables were handled in the analyses. If applicable, describe which groupings were chosen and why | 6 |
| Statistical methods | 12 | (*a*) Describe all statistical methods, including those used to control for confounding | 6 |
| | | (*b*) Describe any methods used to examine subgroups and interactions | N/A |



For peer review only - http://bmjopen.bmj.com/site/about/guidelines.xhtml

| | | | |
|---|---|---|---|
| | | (*c*) Explain how missing data were addressed | Intention to treat. No missing data. |
| | | (*d*) *Cohort study*—If applicable, explain how loss to follow-up was addressed<br>*Case-control study*—If applicable, explain how matching of cases and controls was addressed<br>*Cross-sectional study*—If applicable, describe analytical methods taking account of sampling strategy | |
| | | (*e*) Describe any sensitivity analyses | N/A |

| **Results** | | | |
|---|---|---|---|
| Participants | 13* | (a) Report numbers of individuals at each stage of study—eg numbers potentially eligible, examined for eligibility, confirmed eligible, included in the study, completing follow-up, and analysed | 7 |
| | | (b) Give reasons for non-participation at each stage | 7 |
| | | (c) Consider use of a flow diagram | Not required |
| Descriptive data | 14* | (a) Give characteristics of study participants (eg demographic, clinical, social) and information on exposures and potential confounders | 7 |
| | | (b) Indicate number of participants with missing data for each variable of interest | N/A |
| | | (c) *Cohort study*—Summarise follow-up time (eg, average and total amount) | 7 |
| Outcome data | 15* | *Cohort study*—Report numbers of outcome events or summary measures over time | 7 |
| | | *Case-control study*—Report numbers in each exposure category, or summary measures of exposure | |
| | | *Cross-sectional study*—Report numbers of outcome events or summary measures | |
| Main results | 16 | (*a*) Give unadjusted estimates and, if applicable, confounder-adjusted estimates and their precision (eg, 95% confidence interval). Make clear which confounders were adjusted for and why they were included | 7 |
| | | (*b*) Report category boundaries when continuous variables were categorized | 7 |
| | | (*c*) If relevant, consider translating estimates of relative risk into absolute risk for a meaningful time period | 7 |
| Other analyses | 17 | Report other analyses done—eg analyses of subgroups and interactions, and sensitivity analyses | 8 |
| **Discussion** | | | |
| Key results | 18 | Summarise key results with reference to study objectives | 9 |
| Limitations | 19 | Discuss limitations of the study, taking into account sources of potential bias or imprecision. Discuss both direction and magnitude of any potential bias | 9 |
| Interpretation | 20 | Give a cautious overall interpretation of results considering objectives, limitations, multiplicity of analyses, results from similar studies, and other relevant evidence | 9 |
| Generalisability | 21 | Discuss the generalisability (external validity) of the study results | 10,11 |
| **Other information** | | | |
| Funding | 22 | Give the source of funding and the role of the funders for the present study and, if applicable, for the original study on which the present article is based | 12 |


For peer review only - http://bmjopen.bmj.com/site/about/guidelines.xhtml

BMJ Open

# Bubble-PAPR: a phase 1 clinical evaluation of the comfort and perception of a prototype powered air-purifying respirator for use by healthcare workers in an acute hospital setting

| | |
|---|---|
| Journal: | *BMJ Open* |
| Manuscript ID | bmjopen-2022-066524.R3 |
| Article Type: | Original research |
| Date Submitted by the Author: | 31-Mar-2023 |
| Complete List of Authors: | McGrath, Brendan; Manchester University NHS Foundation Trust; Manchester Academic Health Science Centre, Immunity and Respiratory Medicine<br>Shelton, Clifford; NHS Manchester; Lancaster University Lancaster Medical School<br>Gardner, Angela; Manchester University NHS Foundation Trust<br>Coleman, Ruth; Manchester University NHS Foundation Trust<br>Lynch, James; Manchester University NHS Foundation Trust<br>Alexander, Peter G; Manchester University NHS Foundation Trust; Manchester Academic Health Science Centre, Immunity and Respiratory Medicine<br>Cooper, Glen; The University of Manchester School of Mechanical Aerospace and Civil Engineering |
| <b>Primary Subject Heading</b>: | Intensive care |
| Secondary Subject Heading: | Anaesthesia, Communication, Infectious diseases, Intensive care, Nursing |
| Keywords: | OCCUPATIONAL & INDUSTRIAL MEDICINE, INTENSIVE & CRITICAL CARE, Infection control < INFECTIOUS DISEASES, ANAESTHETICS, RESPIRATORY MEDICINE (see Thoracic Medicine) |
| | |

SCHOLARONE™
Manuscripts

*I, the Submitting Author has the right to grant and does grant on behalf of all authors of the Work (as defined in the below author licence), an exclusive licence and/or a non-exclusive licence for contributions from authors who are: i) UK Crown employees; ii) where BMJ has agreed a CC-BY licence shall apply, and/or iii) in accordance with the terms applicable for US Federal Government officers or employees acting as part of their official duties; on a worldwide, perpetual, irrevocable, royalty-free basis to BMJ Publishing Group Ltd ("BMJ") its licensees and where the relevant Journal is co-owned by BMJ to the co-owners of the Journal, to publish the Work in this journal and any other BMJ products and to exploit all rights, as set out in our licence.*

*The Submitting Author accepts and understands that any supply made under these terms is made by BMJ to the Submitting Author unless you are acting as an employee on behalf of your employer or a postgraduate student of an affiliated institution which is paying any applicable article publishing charge ("APC") for Open Access articles. Where the Submitting Author wishes to make the Work available on an Open Access basis (and intends to pay the relevant APC), the terms of reuse of such Open Access shall be governed by a Creative Commons licence – details of these licences and which Creative Commons licence will apply to this Work are set out in our licence referred to above.*

*Other than as permitted in any relevant BMJ Author's Self Archiving Policies, I confirm this Work has not been accepted for publication elsewhere, is not being considered for publication elsewhere and does not duplicate material already published. I confirm all authors consent to publication of this Work and authorise the granting of this licence.*

For peer review only

# Bubble-PAPR: a phase 1 clinical evaluation of the comfort and perception of a prototype powered air-purifying respirator for use by healthcare workers in an acute hospital setting

**Authors**
Brendan A McGrath, Consultant & Honorary Professor[1,2]
Cliff Shelton, Consultant Clinical Senior Lecturer[1,3]
Angela Gardner, Clinical Fellow[1]
Ruth Coleman, Advanced Critical Care Practitioner[1]
James Lynch, Advanced Critical Care Practitioner[1]
Peter G Alexander, Consultant & Honorary Senior Lecturer[1,2]
Glen Cooper, Reader[4]

1. Acute Intensive Care Unit, Wythenshawe Hospital, Manchester University NHS Foundation Trust, Manchester, UK
2. Manchester Academic Critical Care, Division of Infection, Immunity and Respiratory Medicine, School of Biological Sciences, Faculty of Biology, Medicine and Health, University of Manchester, Manchester Academic Health Science Centre, Manchester, UK
3. Lancaster Medical School, Lancaster University, Lancaster, UK
4. Department of Mechanical, Aerospace & Civil Engineering, University of Manchester, Manchester, UK

**Correspondence to:**
Brendan A McGrath (CI)
Acute Intensive Care Unit, Wythenshawe Hospital, Southmoor Road, Wythenshawe, Manchester M23 9LT, UK
brendan.mcgrath@manchester.ac.uk



For peer review only - http://bmjopen.bmj.com/site/about/guidelines.xhtml

**Abstract**

**Objectives:** We aimed to design and produce a low-cost, ergonomic, hood-integrated Powered Air-Purifying Respirator (Bubble-PAPR) for pandemic healthcare use, offering optimal and equitable protection to all staff. We hypothesised that participants would rate Bubble-PAPR more highly than current FFP3 face mask respiratory protective equipment (RPE) in the domains of comfort, perceived safety and communication.

**Design:** Rapid design and evaluation cycles occurred based on the identified user needs. We conducted diary card and focus group exercises to identify relevant tasks requiring RPE. Lab-based safety standards established against British Standard BS-EN-12941 and EU2016/425 covering materials; inward particulate leakage; breathing resistance; clean air filtration and supply; carbon dioxide elimination; exhalation means; and electrical safety. Questionnaire-based usability data from participating frontline healthcare staff before (usual RPE) and after using Bubble-PAPR.

**Setting:** Overseen by a trial safety committee, evaluation progressed sequentially through laboratory, simulated, low-risk, then high-risk clinical environments of a single tertiary NHS hospital.

**Participants:** 15 staff completed diary cards and focus groups. 91 staff from a range of clinical and non-clinical roles completed the study, wearing Bubble-PAPR for a median of 45 minutes (IQR 30-80 [15-120]). Participants self-reported a range of heights (mean 1.7m [SD 0.1, range 1.5-2.0]), weights (72.4kg [16.0, 47-127]) and body mass indices (25.3 [4.7,16.7-42.9]).

**Outcome measures:** Pre-use particulometer "fit testing" and evaluation against standards by independent biomedical engineer. Primary: perceived comfort (Likert scale). Secondary: perceived safety, communication.

**Results:** Mean fit factor 16,961 (ten participants). Bubble-PAPR mean comfort score 5.64(SD 1.55) versus usual FFP3 2.96(1.44) (mean difference 2.68 (95% CI 2.23-3.14, $p<0.001$). Secondary outcomes, Bubble-PAPR mean(SD) vs FFP3 mean(SD), [mean difference (95%CI)] were: *How safe do you feel?* 6.2(0.9) vs 5.4(1.0), [0.73 (0.45-0.99)]; *Speaking to other staff* 7.5 (2.4) vs 5.1 (2.4), [2.38 (1.66-3.11)]; *Heard by other staff* 7.1 (2.3) vs 4.9(2.3), [2.16 (1.45-2.88)]; *Speaking to patients* 7.8(2.1) vs 4.8(2.4), [2.99 (2.36-3.62)]; *Heard by patients* 7.4(2.4) vs 4.7(2.5), [2.7(1.97-3.43)]; all $p<0.01$.

**Conclusions:** Bubble-PAPR achieved its primary purpose of keeping staff safe from airborne particulate material whilst improving comfort and the user experience when compared with usual FFP3 masks. The design and development of Bubble-PAPR were conducted using a careful evaluation strategy addressing key regulatory and safety steps.

**Trial registration:** ClinicalTrials.gov, NCT04681365.




For peer review only - http://bmjopen.bmj.com/site/about/guidelines.xhtml

**Strengths and limitations of this study**

- We employed user-centred design, engineering optimisation and staged feasibility testing to develop a novel Powered Air-Purifying Respirator (Bubble-PAPR) for use specifically in frontline healthcare settings.
- The design of Bubble-PAPR met regulatory standards and our evaluation demonstrated that it met the key requirements of comfort and perceived safety identified as essential requirements by healthcare staff.
- The design and development of Bubble-PAPR were conducted using a careful strategy addressing key regulatory and safety steps, measured against UK/European standards, in contrast to many devices rapidly developed and deployed during the pandemic.
- The development of Bubble-PAPR is an excellent example of growing a cosmopolitan network (social networks across historical, political, and cultural boundaries).
- Limitations of our study include that design and evaluation were undertaken at a single large hospital, using similar staff groups, and a lack of formal independent cost analysis.

For peer review only

## Introduction

The COVID-19 global pandemic created a worldwide shortage of personal protective equipment (PPE)[1] and highlighted significant usability issues in current PPE products.[2] In addition to direct contact, airborne diseases may be spread by aerosol or droplet transmission. Aerosol transmission may be mitigated by the appropriate use of respiratory protective equipment (RPE), a particular classification of personal protective equipment (PPE). However, respiratory protective equipment is used as part of a hierarchy of control measures. This is because RPE only protects individual workers, is prone to failure or misuse (wrong RPE for the wrong task/environment) and wearers may get a false sense of security, which may lead to neglect of other aspects of infection prevention and control, such as isolation requirements.[3] A range of inspiratory filtering devices exist: dust masks, half-face masks, full-face masks and powered (fan-assisted) respirators. Powered respirators include: half/full-face masks, helmets, hoods and visors. Though not used in healthcare, for completeness, breathing apparatuses are systems that supply an independent, positive pressure supply of breathing-quality air.

Face masks may be classified by considering the level of protection they offer the wearer to inhalation of environmental contaminants. Simple surgical face masks or 'nuisance' dust masks do not entirely filter droplets or aerosols. Filtering face piece (FFP) masks comprise layers of synthetic non-woven material with interleaved filtration layers and provide protection against small airborne particles (aerosols). Different types and constructions of FFP masks can be classified by their ability to filter small particles. Particulate filters can be classified as low (P1) to high (P3) efficiency, filtering between 80% of particles smaller than 2 micrometres to 99.95% of particles smaller than 0.5 micrometres, respectively (Box 1).[4] Respiratory protection can therefore be considered in terms of a combination of the filtering ability of the device relative to the exposure environment and its fit on the wearer's face. A device is considered adequate if it has the capacity to reduce the wearer's exposure to a hazardous substance to acceptable levels (to comply with occupational exposure limit values). Devices can be reusable, but the majority are single use. Masks are difficult to recycle due to their layered construction and the pandemic contributed to an unprecedented rise in RPE-related clinical waste.[5]

The majority of RPE used in healthcare settings are disposable face masks adopted from industry. Masks are not designed to be worn for long periods or repeated shifts, may restrict the visual field, limit communication, cause facial damage due to their tight fit, and require multiple time-consuming 'fit tests' for each model of the device for each staff member. All these issues were highlighted in the context of the 2002-2004 SARS epidemic.[6] More appropriate solutions for prolonged and repeated use include powered air-purifying respirators (PAPRs). But, again, these are not designed primarily for healthcare, are heavy, noisy, expensive, difficult to clean to clinical standards, and not suitable for the specific needs in frontline healthcare environments.

There have been several widely reported 'homemade' or 'MacGyvered' devices that well-intentioned groups or individuals developed to protect staff and patients during the pandemic.[7] In a time of crisis, these innovations were often rapidly developed without significant funding and delivered to areas of need during a time of global RPE shortage. However, due to the urgency of the situation, few of these devices sought or achieved independent certification or provided data to support safety.[8] Turner and colleagues proposed a framework for the safer adoption of novel devices[7] which: defines the problem and reviews existing solutions, benchmarks safety indices for the devices, and then evaluates it in a structured manner through simulated, low- and then high-risk clinical settings (Table S1



For peer review only - http://bmjopen.bmj.com/site/about/guidelines.xhtml

[Supplemental]). Broad stakeholder feedback is encouraged through iterative review cycles, re-design and improvements.

**Box 1.** Classification of particulate filters, with a worked example and fit testing
Data from EU Standard 149:2001 Respiratory Protective Devices

**P1** – Filters about 80% of particles smaller than 2 micrometres
**P2** – Filters about 94% of particles smaller than 0.5 micrometres
**P3** – Filters about 99.95% of particles smaller than 0.5 micrometres

A respiratory protective device is considered adequate if it has the capacity to reduce the wearer's exposure to a hazardous substance to acceptable levels. The ratio of airborne particles outside:inside the filtering device gives a **nominal (theoretical) protection factor**. An **assigned protection factor** reflects the actual workplace conditions. For example: an airborne dust contaminant with an occupational exposure limit of 5mg/m$^3$ may be present in the workplace in concentrations up to 60mg/m$^3$ (determined by monitoring). A particle filter is needed to reduce the concentration by at least a factor of 12 (60/5=12). A P3 filter with an assigned protection factor of 20 would be suitable (as this is greater than the factor of 12 required). Other considerations such as exposure time, useability and disposal of the device need to be considered prior to undertaking a **fit test** with the intended wearer.

A fit test verifies that a **specific model** of device works as intended with a **particular individual**. For example, different face shapes and facial hair can interfere with a particular system's ability to filter environmental contaminants effectively.

**Qualitative fit testing** assesses the inward leakage past a mask of airborne compounds detectable by the wearer (typically bitter/sweet tasting substances), aerosolised using a spray device.

**Quantitative fit testing** measures particulate concentrations inside and outside of devices, typically undertaken by measuring sodium chloride aerosolised in water to generate a 'particle' count. Quantitative fit testing generates a **fit factor** – the ratio of airborne particle counts outside:inside. The fit factor takes account of the whole device (the filter, hood and airflow in the case of a PAPR). Fit factors for PAPRs are very high (optimal protection) and so if correctly worn, fit testing prior to use is not usually required.

Considering the above, our project aimed to design and produce a low-cost, ergonomic, hood-integrated PAPR for use in frontline healthcare settings. Our objectives were to focus on user-centred design, engineering optimisation, staged feasibility testing, certification, intellectual property protection and then rapid manufacture and distribution. We also aimed to design the PAPR to be re-used, refurbished and recycled where possible, using readily available, simple and interchangeable key parts which proved difficult to source during the early stages of the pandemic. Finally, by designing an available, affordable PAPR system that could be cleaned appropriately and re-used between different staff, we aimed to provide equitable access to high-quality RPE that offered optimal protection to *all* staff, wherever they worked.[9] In this phase 1 clinical evaluation, we hypothesised that participants would rate Bubble-PAPR more highly than current FFP3 face mask RPE across the domains of comfort, perceived safety and communication.


For peer review only - http://bmjopen.bmj.com/site/about/guidelines.xhtml

**Methods**

The design team brought together frontline clinical staff based in the Wythenshawe Hospital Acute Intensive Care Unit (ICU) of Manchester University NHS Foundation Trust (MFT), an experienced product design consultancy (Designing Science Limited, Middlesex, UK) and the technical expertise of the School of Engineering at the University of Manchester (UoM). Research Ethical and Health Research Authority approval (IRAS ID:288493, REC Ref:21/WA/0018) was granted. The study was sponsored by MFT, who acted as the manufacturer of this in-house prototype device, which became known as Bubble-PAPR. The study protocol, analysis plan and recruitment metrics were registered and reported at ClinicalTrials.gov (NCT04681365). Participating staff were provided with participant information sheets, a detailed explanation and demonstration of the safe use of Bubble-PAPR, and written consent was obtained. User needs assessment was conducted through a series of workplace diary card exercises documenting typical activities undertaken by frontline healthcare staff, synthesised in focus groups. Staff were invited to participate (by email and posters in rest areas) from clinical locations where RPE was mandated within the hospital. The first two respondents from each area were recruited to the diary card and focus group activities. Rapid design and evaluation cycles occurred based on the identified user needs. In addition, evaluation of early prototypes occurred in simulated clinical environments, collecting usability data from participants.

Patient and public involvement

Patient and public involvement was undertaken through the Manchester Academic Critical Care research group's patient forum. There were powerful accounts from patients who regularly described not being able to understand what hospital staff wearing PPE were saying and being troubled that they had no idea what their carers looked like. These reports led us to focus on prioritising the ease of communication with Bubble-PAPR. Staff participants who were invited to wear Bubble-PAPR were recruited from clinical locations where RPE was mandated, by direct invitation from the research team.

Study procedures

A Trial Safety Committee was established to oversee the results of laboratory and bench testing of the prototype, initial safety data, usability, and adverse event data at each stage of the evaluation. The Committee met prior to commencing clinical evaluation. It was tasked with the decision to allow the evaluation to proceed between phases: simulated clinical environment, low-risk (non-infectious) clinical environment and high-risk clinical environment (COVID-19 wards and ICUs). Early iterations of Bubble-PAPR included 3-D-printed collars and key parts (such as the impellor), along with a variety of designs of the hood. A final iteration of Bubble-PAPR included a medical-grade foam collar, precision-machined internal components and a revised (smaller) hood was further tested in high-risk environments. Prior to first use, several device safety checks were independently undertaken by the MFT Electrical and Biomedical Engineering Department and INSPEC International Ltd, Salford, UK). A short report addressing the quantitative and qualitative criteria detailed in the relevant standards, and summarised in Tables S2, S3 and S5 (Supplemental), was presented to the Trial Safety Committee. The first ten study participants to wear Bubble-PAPR underwent ‘fit testing’ with a particulometer (TSI Portacount Fit Tester 8040, TSI Instruments Ltd, Buckinghamshire, UK) following a standard protocol derived from the UK Government’s Health and Safety Executive.[10] Fit testing is not required before wearing PAPRs, including Bubble-PAPR. The purpose of fit testing was to collect device performance



For peer review only - http://bmjopen.bmj.com/site/about/guidelines.xhtml

data and to allow the research team to assure the Trial Safety Committee that Bubble-PAPR was performing to an appropriate standard. This INDG-479 protocol requires a 'Fit Factor' pass level of 100 for FFP3/N95 face masks and 500 for full face masks/hoods. Participants followed this standard protocol during quantitative fit testing which involved the following exercises undertaken for at least 60 seconds: normal breathing; deep breathing; turning head from side-to-side; moving head up and down; talking; bending over to 90 degrees; repeat normal breathing. European Conformity Standard EN12941 requires an applied fit factor of 40 for a 'loose-fitting hood' PAPR; the equivalent of a nominal protection factor of at least 500 (accepting an inward leakage of 0.2% with a P3 class filter see Box 1). By comparison, the minimal fit factor for an FFP3 mask in a clinical environment is 100. Tests were conducted in an ICU side room with a particle generator to reach background counts between 70,000 to 100,000 particles/$cm^3$.

Outcomes

The primary outcome was based on Davis' technology acceptance model (perceived usefulness and perceived ease-of-use overcoming barriers to adoption)[11]. First, staff were asked to rate their experiences using current RPE (a variety of re-useable or disposable FFP3 masks) using a series of questions based on Likert-type scales (see Supplemental material). Next, safe use of the Bubble-PAPR was explained, and instructions for use were provided, supported by videos of donning, doffing, cleaning and storage. Bubble-PAPR was then worn during simulated/clinical use where the usual tasks were undertaken (identified in the focus groups, including verbal communication between colleagues and patients; writing; typing; reading notes, computer screens and monitors; manual handling; invasive procedures; emergency resuscitation; airway management; and maintenance of a clean/safe bedside environment). In order to evaluate critical communication and the stability of the Bubble-PAPR, the simulated environment tests also included high-stakes team-based tasks such as managing a cardio-respiratory arrest, cardiopulmonary resuscitation, assessment and management of the critically ill patient and complex airway management. Finally, after removal (doffing) of Bubble-PAPR, staff were immediately invited to complete a second questionnaire focused on the prototype. Free text comments were also invited.

The primary endpoint was staff rating of the comfort of Bubble-PAPR (versus current FFP3 face masks). Secondary endpoints focused on communication and perceived safety. Specifically, this was staff ratings of the prototype in terms of: how safe participants felt, ease of communication with colleagues, and ease of communication with patients (again, Bubble-PAPR versus current FFP3 face masks). Additional questions explored wearer anxiety, ease of use, and performance whilst undertaking usual work tasks. In parallel, in-house device feasibility testing was conducted in the hospital environment to test ergonomics and air particle filtration. The research framework for this study was based around in-house exemption for device development from the UK Medicines and Healthcare products Regulatory Agency (MHRA). This means that the hospital, acting as manufacturer, can use a device it has developed itself internally. Such a device is not required to undergo to independent testing and therefore it will not achieve a certificate of conformity (UK-Conformity Assessed or Conformitè Europëenne marking). However, in order to assure the study Sponsor and staff participants of the safety and efficacy of Bubble-PAPR, we tested against existing conformity standards for PAPRs relevant at the time of development (British Standard BS EN 12941 [Respiratory Protective Devices: Powered filtering devices incorporating a helmet or hood] and the European Union Personal Protective Equipment Directive EU2016/425). [4, 12] Some of the testing was undertaken


For peer review only - http://bmjopen.bmj.com/site/about/guidelines.xhtml

internally by independent biomedical engineers, with the flowrate and carbon dioxide testing undertaken externally.

Sample size and statistical analysis

A pilot evaluation was conducted in August 2020 to test the questionnaires and to assess the likely population means for the test scores (Table S3 [Supplemental]). We calculated a sample size of 20 participants would be required for each phase of the evaluation to detect a significant difference between usual PPE and Bubble-PAPR, based on a mean difference of 2.5 (SD 0.9) points on the 7-point Likert scale identified during the pilot evaluation (alpha = 0.05, 90% power). In addition, we allowed for a 5% dropout and missing data rate, concluding 22 participants per phase. All variables were explored via appropriate graphical and descriptive statistics to evaluate distributions, data completeness and form. Analyses were conducted in RStudio 2020 (Boston, MA, www.rstudio.com). Analyses were performed separately for each phase for presentation to the Trial Safety Committee, with a pooled analysis conducted at the study conclusion. Comparisons between groups (current RPE vs Bubble-PAPR) were made using a paired t-test or Wilcoxon signed-rank test as appropriate.

For peer review only



For peer review only - http://bmjopen.bmj.com/site/about/guidelines.xhtml

**Results**

The final design of Bubble-PAPR is shown schematically in Figure 1 (www.bubble-papr.com, with detailed technical drawings available by searching the patent number [PCT/GB2021/052147] at www.espacenet.com). The device safety checks and fit testing results are presented in Tables S2, S3 and S4 (Supplemental), respectively, demonstrating a mean fit factor of 16,961. Additional particulometer tests were undertaken with deliberate tears up to 20 cm in the hood using a dummy head. The lowest fit factor recorded with the damaged hood was 1,123. Therefore, the Trial Safety Committee concluded that the Bubble-PAPR performed its primary purpose of adequately protecting staff from airborne environmental contaminants.

Fifteen staff contributed to the diary and focus group exercises. Nurses (n=7), Doctors (4) Physiotherapists (2), Advanced Practitioners (1), Speech and Language Therapists (1) representing Emergency Medicine, Critical Care, Orthopaedics and Obstetric specialties generated a list of tasks to be undertaken. One staff member from the 16 invited could not attend the focus group meeting. Staff reported a range of patient-facing activities, including: verbal communication between colleagues and patients; writing; typing; reading notes, computer screens and monitors; manual handling; invasive procedures; emergency resuscitation; airway management; and maintenance of a clean/safe bedside environment. Over the course of the evaluation, staff completed all of the tasks identified by the diary exercise whilst wearing Bubble-PAPR in the clinical environment. Ninety-one staff wore Bubble-PAPR for a median of 45 (IQR 30-90, range 10-150) minutes between 3rd March and 21st December 2021. All relevant staff working in relevant clinical areas were approached until a maximum of six staff had been recruited per shift (the most that the research team could reasonably accommodate per shift), or the recruitment target had been met. No staff who were approached during their clinical shifts were unwilling or unable to trial Bubble-PAPR. There were no Bubble-PAPR-related safety incidents reported during the study. Staff undertook all clinical duties identified by the focus groups and diary card exercise, either in the simulation suite (n=22) or clinical settings (n=22 low-risk, n=25 high-risk, n=22 high-risk with final iteration). Participants predominantly declared as female (69%), and were from a range of clinical and non-clinical roles, Figure S1 (Supplemental). Staff self-reported a range of heights (mean 1.7m [SD 0.1, range 1.5-2.0]), weights (72.4kg [16.0, 47-127]) and body mass indices (25.3 [4.7,16.7-42.9]), Figure S2 (Supplemental). Fifty-two percent of participants reported that they normally wore glasses, with 31% wearing glasses during the evaluation. All participants described at least 6 month's experience with FFP3 face masks on a regular basis ("most shifts"), with a combination of re-useable (typically 3M™ 6000 Series Respirators) and single use (typically 3M™ Aura™ 9330 or equivalent) face masks. No participants described using PAPRs in the six months prior to recruitment. All participants completed all mandatory questionnaire sections.

With pooled data for the primary outcome, "*How comfortable do you feel in your PPE?*" (Likert scale bounded by 1 [very uncomfortable] to 7 [very comfortable]), Bubble-PAPR mean score was 5.64 (SD 1.55) versus usual FFP3 face mask 2.96 (1.44; Figure 2). There was a mean difference of 2.68 (95% CI 2.23-3.14, p<0.001). Secondary outcomes focused on communication and perceived safety. For the question, "*How safe do you feel in your PPE?*", Bubble-PAPR mean score was 6.15 (0.94) vs usual FFP3 face mask 5.43 (0.98); mean difference 0.73 (95% CI 0.45-1.00, p<0.001; Figure 2). Figure 3 demonstrates communication outcomes for all 91 comparisons of Bubble-PAPR versus usual FFP3 face


For peer review only - http://bmjopen.bmj.com/site/about/guidelines.xhtml

masks. All adjusted comparisons were significant (p<0.001) in favour of Bubble-PAPR for communicating with both colleagues and patients (Table 1 and Table S5 [Supplemental]).

Secondary outcomes where a lower Likert response was considered better are presented in Figure S3 (Supplemental). These focussed on whether staff were worried about themselves or others whilst wearing RPE, whether the devices caused pressure or pain or if communication was impaired. Finally, staff were asked if they had to cut short a clinical (or simulated) encounter due to discomfort with their RPE. Again, there was a significant difference in favour of Bubble-PAPR for all metrics (all p<0.001, Table 1 and Table S5 [Supplemental]).

For peer review only



For peer review only - http://bmjopen.bmj.com/site/about/guidelines.xhtml

**Table 1.** Rating scales, summary results and comparisons across the primary outcome questionnaire domains

| | PPE | Q8 Speak to staff | Q9 Be heard by staff | Q10 Speak to patient | Q11 Be heard by patient | Q14 How safe does it feel | Q17 Comfortable |
|---|---|---|---|---|---|---|---|
| Rating scale | From: | 0 - no confidence | 0 - no confidence | 0 - no confidence | 0 - no confidence | 1 – very unsafe | 1 – very uncomfortable |
| | To: | 10 - fully confident | 10 - fully confident | 10 - fully confident | 10 - fully confident | 7 very safe | 7 very comfortable |
| | | | | | | | |
| RPE type | FFP3 | 5.1 (2.4) [1 - 10] | 4.9 (2.3) [1 - 10] | 4.8 (2.4) [1 - 10] | 4.7 (2.5) [1 - 10] | 5.4 (1.0) [3 – 7] | 3 (1.4) [1 – 6] |
| | Bubble | 7.5 (2.4) [1 - 10] | 7.1 (2.3) [1 - 10] | 7.8 (2.1) [2 - 10] | 7.4 (2.4) [1 - 10] | 6.2 (0.9) [3 – 7] | 5.6 (1.6) [1 – 7] |
| Comparison | Mean difference | 2.38 | 2.16 | 2.99 | 2.7 | 0.73 | 2.68 |
| | 95% CI | 1.66 to 3.11 | 1.45 to 2.88 | 2.36 to 3.62 | 1.97 to 3.43 | 0.45 to 0.99 | 2.23 to 3.14 |
| | | Favours Bubble | Favours Bubble | Favours Bubble | Favours Bubble | Favours Bubble | Favours Bubble |
| | Adjusted p | <0.001 | <0.001 | <0.001 | <0.001 | <0.001 | <0.001 |

For peer review only



For peer review only - http://bmjopen.bmj.com/site/about/guidelines.xhtml

During the initial phases, there was no significant difference between staff reporting ease of donning and doffing of Bubble-PAPR and usual PPE (the FFP3 face masks which staff had used for many months at the time of the evaluation). However, pooled results saw staff becoming more familiar with the Bubble, and Bubble-PAPR was rated easier to don and doff when compared with usual FFP3 face masks (adjusted p=0.003 and 0.002 respectively), Table 1 and Figure S4 (Supplemental). One hundred and thirty-two additional free text comments were reviewed and categorised into positive (n=47, 35.6%), negative (67, 50.8%) and neutral (18, 13.6%) comments (Figures S5-7 [Supplemental]). Most comments focused on the noise of the device, which improved throughout the project as the impellor and motor were made quieter in later design iterations. The categories and nature of comments were as follows: Noise (33 comments [3 neutral, 30 negative]); Comfort (24 comments [20 positive, 2 neutral, 2 negative]); Communication (22 comments [5 positive, 6 neutral, 11 negative]); General (21 comments [17 positive, 2 neutral, 2 negative]); Vision (14 comments [1 positive, 4 neutral, 9 negative]); Wear and fit (10 comments (2 positive, 1 neutral, 7 negative); Stethoscope (5 negative comments); Safety (2 positive comments); Battery (1 negative comment).

For peer review only



For peer review only - http://bmjopen.bmj.com/site/about/guidelines.xhtml

**Discussion**

Our project developed an innovative prototype PAPR explicitly designed for prolonged healthcare use in high-risk clinical environments. Bubble-PAPR achieved its primary purpose of protecting staff by exceeding recognised safety standards for PAPRs, whilst also being rated significantly higher for comfort (the primary outcome), perceived safety, and communication with colleagues and patients (secondary outcomes) than usual FFP3 face masks. Bubble-PAPR was used in all relevant simulated and clinical scenarios identified by detailed staff diary cards, making the results of this study extremely relevant to hospital-based healthcare workers.

Bubble-PAPR was rapidly developed based on the lived experiences of frontline staff during the early stages of the coronavirus pandemic, addressing the unmet needs of reliable, high-quality, universal and available RPE with improved comfort and communication when compared to usual FFP3 face masks. Staff overwhelmingly recognised the importance of facial visualisation when communicating with colleagues and patients. When combined with the improved comfort of wearing a PAPR over usual RPE, participants rated Bubble-PAPR consistently highly across all comparator domains.

This relatively simple evaluation study was preceded by a rapid design and prototyping phase, producing a working prototype within a few weeks. Despite the speed and agility demonstrated by the design team, we adhered to relevant conformity standards for PAPRs, following a tiered evaluation within the governance structure of an approved and regulated research project. Bubble-PAPR was only introduced into higher-risk environments following review by the Trial Safety Committee. This structured approach contrasted with some other rapidly developed or adopted pandemic RPE systems.[7, 13, 14] Whilst the PPE shortages experienced during the pandemic drove many of these innovations and adaptations, we recognised the importance of a methodical approach to design, development and testing of our prototype, both in the laboratory and clinical settings. We recommend others to follow the framework proposed by Duggan et al. for the development of novel medical devices, with regular reviews of safety and useability data within the framework of a robust and transparent clinical trial.[7] The development of Bubble-PAPR required the rapid formation of a cosmopolitan network of frontline healthcare staff, designers, engineers, academics, innovators, marketing experts, manufacturers and funders. Our collaborative had not all worked together before and members crossed historical, political, and cultural boundaries to work effectively together. Post-pandemic, cosmopolitan networks such as this could become a key feature of future system resilience and facilitate new ways of working.

Our study has some limitations. Some of the endpoints were self-reported by participating staff and not independently verified. This included communication between colleagues, and between staff and patients. However, staff were performing their usual clinical duties whilst wearing Bubble-PAPR and any limitations of two-way communication were recognised and reported. The design of Bubble-PAPR addressed many of the issues identified by the same staff who subsequently evaluated the prototype. Whilst our study protocol allowed evaluation only within our Trust owing to the 'in-house' manufacturing exemption for testing, it is not unreasonable to expect similar results if our prototype were evaluated elsewhere. Although this may be considered a weakness of the study, many of the shortcomings of the PPE provided to frontline health workers around the world are well described and are essentially the same as those identified in our project.[15, 16] Furthermore, we evaluated Bubble-


For peer review only - http://bmjopen.bmj.com/site/about/guidelines.xhtml

PAPR against single-use and reusable FFP3 face masks, which could be construed as comparing two different classes of RPE. However, Bubble-PAPR was designed and developed to provide a viable alternative to FFP3 class face masks, in contrast to the more usual healthcare use of PAPRs. Other PAPRs are more complex, more cumbersome (belt-worn fans and hoses), more costly, and typically are selectively available on a limited basis to specific users or groups because of these factors. Although a pricing structure is currently unavailable, the simplicity of the design and components (designed with pandemic supply chain limitations in mind) means that Bubble-PAPR is likely to cost around 25-50% of the list price of equivalent PAPRs. Our detailed analysis of work diary cards from various clinical staff ensured that Bubble-PAPR was used for all relevant procedures identified by participating staff in our settings that were undertaken by medical, nursing, healthcare assistant, allied healthcare professional (speech and language therapy, physiotherapy, pharmacy), administrative and domestic staff in the clinical area. Staff were able to undertake their usual duties with this simple, collar-worn PAPR. Limitations of the design include the inability to use a conventional stethoscope (although Bluetooth stethoscopes were used effectively), potential visual distortions if the visor section of the hood became creased, and the residual noise during use (common amongst PAPRs). Although the design is simple, with visual/mechanical indicators instead of electronic indicators or alarms, this did not impact on conformity testing or function. Addressing the actual activities undertaken by specific staff groups, testing safety, performance and the user experience, is unique within published respiratory protective equipment product evaluation studies.[17, 18] High acuity activities such as CPR and tracheal intubation were undertaken whilst wearing Bubble-PAPR but we collected data only around perceived comfort, safety and self-reported efficacy. Bubble-PAPR meets current industrial standards for the safe use of respiratory protection, but such standards are not usually designed with healthcare procedures in mind. Post-pandemic conformity requirements will vary around the world and future iterations of Bubble-PAPR may need to adapt to meet country-specific requirements.

Our study did not directly evaluate the patient experience with staff wearing different RPE. However, the patient experience was reflected in the user specifications identified around communication, and anecdotal feedback was positive from patients, especially around facial visibility and verbal and non-verbal communication. In addition, when contrasted with FFP3 face masks, speech and language therapists reported that demonstrating speech and swallow exercises was suddenly possible with Bubble-PAPR and that the transparent nature of the hood overcame the communication barriers that can be so devastating for those with hearing impairments.[19] Although designed to be potentially recyclable, future work should address the environmental impact of PVC hoods with reusable collars compared to single-use or reusable FFP3 face masks.



For peer review only - http://bmjopen.bmj.com/site/about/guidelines.xhtml

**Conclusions**

Our study has demonstrated that Bubble-PAPR achieved its primary purpose of keeping staff safe from airborne particulate material whilst improving comfort, communication and the user experience when compared with usual FFP3 face masks worn throughout the pandemic. It is likely that the patient experience was also enhanced. Bubble-PAPR has been patented (PCT/GB2021/052147) and subsequently licenced to a UK-based healthcare manufacturer for large-scale manufacture and distribution to frontline NHS and other workers. The pandemic drove unprecedented collaboration between clinicians, academics and industry. The development of Bubble-PAPR is an excellent example of growing a cosmopolitan network across historical, political, and cultural boundaries that could become a key feature of future system resilience.

For peer review only



For peer review only - http://bmjopen.bmj.com/site/about/guidelines.xhtml

**Acknowledgements**
We are grateful to our funders (detailed below) for supporting this project and the staff who participated in the evaluation. In addition, we are indebted to the designers, engineers and staff who gave their time freely during the early stages of the coronavirus pandemic to work tirelessly on designing, building, testing and refining Bubble-PAPR. Specifically: Patrick Hall, Designing Science Ltd (www.designingscience.co.uk); Andrew Spragg, Industrial design consultant; Andrew Forbes, XK Design Ltd; James Corden, Manchester University Hospital NHS Foundation Trust Innovation Team; Nick Duggan, Innovation Consultant, Zuas (www.zuas.io); and GAMA Healthcare Ltd, Hemel Hempstead, UK (www.gamahealthcare.com).

**Competing interests**
Manchester University NHS Foundation Trust, the University of Manchester and Designing Science Ltd have agreed commercial terms to license Bubble-PAPR for manufacture and development. No other competing interests are declared.

**Funding**
This project was supported by unrestricted grants and funding from Engineering and Physical Sciences Research Council (EPSRC) Impact Acceleration Account 302, Oxford Road Corridor (grant award number N/A), Health Innovation Manchester 'Momentum' special projects fund 2021 (grant award number N/A), Acute ICU Charitable Research Fund, Manchester University NHS Foundation Trust (grant award number N/A), and Manchester University NHS Foundation Trust (grant award number N/A).

**Data availability statement**

Due to the commercial sensitivity of the intellectual property licensed at the conclusion of this project, the full dataset is not publicly available. However, the corresponding author will consider requests to disclose the dataset on an individual basis as necessary.

**Ethics approval and registration**

Research Ethical and Health Research Authority approval (IRAS ID:288493, REC Ref:21/WA/0018) was granted from Wales REC5 on 27th January 2021. The study was sponsored by Manchester University NHS Foundation Trust, who acted as the manufacturer of this in-house prototype device. The study protocol, analysis plan and recruitment metrics were registered and reported at ClinicalTrials.gov (NCT04681365).

**Contributors**
All authors critically revised the manuscript for important intellectual content and approved the final manuscript. BAM attests that all listed authors meet authorship criteria and that no others meeting the criteria have been omitted. BAM: conception and design, collection, analysis and interpretation of data, drafting and revision of the manuscript, and final approval of the version to be published. Participant recruitment. CS: qualitative work package conception and design, analysis and interpretation of data. Participant recruitment. Drafting and revision of the final manuscript, and final approval of the version to be published. AG: qualitative work package design, analysis and interpretation of data. Drafting and revision of the final manuscript, and final approval of the version to be published. RC: design, collection, and interpretation of data, drafting and revision of the



For peer review only - http://bmjopen.bmj.com/site/about/guidelines.xhtml

manuscript, and final approval of the version to be published. JL: design, collection, and interpretation of data, drafting and revision of the manuscript, and final approval of the version to be published. PGA: design, collection, and interpretation of data, drafting and revision of the manuscript, and final approval of the version to be published. GC: design, collection, and interpretation of data, drafting and revision of the manuscript, and final approval of the version to be published. Manufacturing and engineering lead.

**Provenance and peer review**

Not commissioned; externally peer reviewed.

For peer review only



For peer review only - http://bmjopen.bmj.com/site/about/guidelines.xhtml

## References


1. Fadela Chaib, World Health Organisation. Shortage of personal protective equipment endangering health workers worldwide. 3rd March 2020. Available from www.who.int/news/item/03-03-2020-shortage-of-personal-protective-equipment-endangering-health-workers-worldwide [Accessed 5th May 2020].

2. Hignett S, Welsh R, Banerjee J. Human factors issues of working in personal protective equipment during the COVID-19 pandemic. *Anaesthesia* 2021; 76: 134-135.

3. Cook TM. Personal protective equipment during the coronavirus disease (COVID) 2019 pandemic - a narrative review. *Anaesthesia* 2020; 75: 920-927.

4. British Standard BS EN12941 Respiratory protective devices - Powered filtering devices incorporating a helmet or a hood - Requirements, testing, marking (British Standard). 1999. Available from https://standards.globalspec.com/ [Accessed 5th April 2020].

5. Rizan C, Reed M, Bhutta MF. Environmental impact of personal protective equipment distributed for use by health and social care services in England in the first six months of the COVID-19 pandemic. *Journal of the Royal Society of Medicine* 2021; 114: 250-263.

6. Foo CC, Goon AT, Leow YH, Goh CL. Adverse skin reactions to personal protective equipment against severe acute respiratory syndrome--a descriptive study in Singapore. *Contact Dermatitis* 2006; 55: 291-294.

7. Turner MC, Duggan LV, Glezerson BA, Marshall SD. Thinking outside the (acrylic) box: a framework for the local use of custom-made medical devices. *Anaesthesia* 2020; 75: 1566-1569.

8. Gould CL, Alexander PDG, Allen CN, McGrath BA, Shelton CL. Protecting staff and patients during airway management in the COVID-19 pandemic: are intubation boxes safe. *British Journal of Anaesthesia* 2020; 125: e292-e293.

9. Shelton C, El-Boghdadly K, Appleby JB. The 'haves' and 'have-nots' of personal protective equipment during the COVID-19 pandemic: the ethics of emerging inequalities amongst healthcare workers. *Journal of Medical Ethics* 2021; medethics-2021-107501.

10. Great Britain Health and Safety Executive. Guidance on Respiratory Protective Equipment (RPE) Fit Testing: (Rev1). March 2019. Available from https://www.hse.gov.uk/pubns/indg479.htm [Accessed 5th April 2020].

11. Davis FD. Perceived Usefulness, Perceived Ease of Use, and User Acceptance of Information Technology. *MIS Quarterly* 1989; 13: 319-340.

12. European Agency for Health and Safety at Work. Regulation (EU) 2016/425 on Personal Protective Equipment. 2016. Available from https://osha.europa.eu/en/legislation/directive/regulation-eu-2016425-personal-protective-equipment. [Accessed 5th April 2020].

13. Duggan LV, Marshall SD, Scott J, Brindley PG, Grocott HP. The MacGyver bias and attraction of homemade devices in healthcare. *Canadian Journal of Anesthesia/Journal Canadien d'Anesthésie* 2019; 66: 757-761.

14. Addi RA, Benksim A, Cherkaaoui M. Easybreath Decathlon Mask: An Efficient Personal Protective Equipment (PPE) against COVID-19 in Africa. *Journal of Clinical and Experimental Investigations* 2020; 11: em00738.

15. Kim H, Hegde S, LaFiura C et al. Access to personal protective equipment in exposed healthcare workers and COVID-19 illness, severity, symptoms and duration: a population-based case-control study in six countries. *BMJ Global Health* 2021; 6: e004611.

16. Hoernke K, Djellouli N, Andrews L et al. Frontline healthcare workers' experiences with personal protective equipment during the COVID-19 pandemic in the UK: a rapid qualitative appraisal. *BMJ Open* 2021; 11: e046199.



For peer review only - http://bmjopen.bmj.com/site/about/guidelines.xhtml
For peer review only

17. Galanis P, Vraka I, Fragkou D, Bilali A, Kaitelidou D. Impact of personal protective equipment use on health care workers' physical health during the COVID-19 pandemic: A systematic review and meta-analysis. *American Journal of Infection Control* 2021; 49: 1305-1315.

18. Houghton C, Meskell P, Delaney H et al. Barriers and facilitators to healthcare workers' adherence with infection prevention and control (IPC) guidelines for respiratory infectious diseases: a rapid qualitative evidence synthesis. *Cochrane Database of Systematic Reviews* 2020; 4: CD013582.

19. Poostchi A, Kuet ML, Richardson PS, Patel MK. Covid-19: face masks can be devastating for people with hearing loss but alternatives are available. *BMJ* 2020; 370: m3326.

**Figure legends**

**Figure 1.** Bubble-PAPR comprises a medical-grade foam neck collar and a separate PVC hood. The universal fit collar draws air in through a filter via an impellor powered by an external battery. The collar has a mechanical low flow indicator and can be cleaned and reused by different users. The semi-rigid hood is pulled over the collar before donning and is secured by integrated straps.

**Figure 2.** Reported comfort (primary) and safety (secondary) outcomes for Bubble-PAPR vs usual FFP3 face masks

**Figure 3.** Secondary communication outcomes where a higher Likert scale response was considered better

**Supplemental material legends**

**Table S1 (Supplemental).** Proposed framework for the safer adoption of a MacGyvered device. Adapted from Turner and colleagues.[7]

**Table S2 (Supplemental).** Lab-based testing of the Bubble PAPR prior to clinical evaluation.

**Table S3 (Supplemental).** Pilot data. Q14 & Q17 are Likert Scale items (rated 1-7) and Q8-11 are Visual Analogue Scale items (rated 0-100).

**Table S4 (Supplemental).** Fit testing data from the first 10 participants.

**Table S5 (Supplemental).** Rating scales, summary results and comparisons across all of the questionnaire domains.

**Figure S1 (Supplemental).** Participant job roles.

**Figure S2 (Supplemental).** Self-reported weight, height and BMI of staff participants.

**Figure S3 (Supplemental).** Secondary outcomes where a lower Likert scale response was considered better.

**Figure S4 (Supplemental).** Ease of donning and doffing of Respiratory Protective Equipment.

**Figure S5 (Supplemental).** Word clouds from the free text feedback. Negative comments (all categories).

**Figure S6 (Supplemental).** Word clouds from the free text feedback. Neutral comments (all categories).

**Figure S7 (Supplemental).** Word clouds from the free text feedback. Positive comments (all categories).


For peer review only - http://bmjopen.bmj.com/site/about/guidelines.xhtml

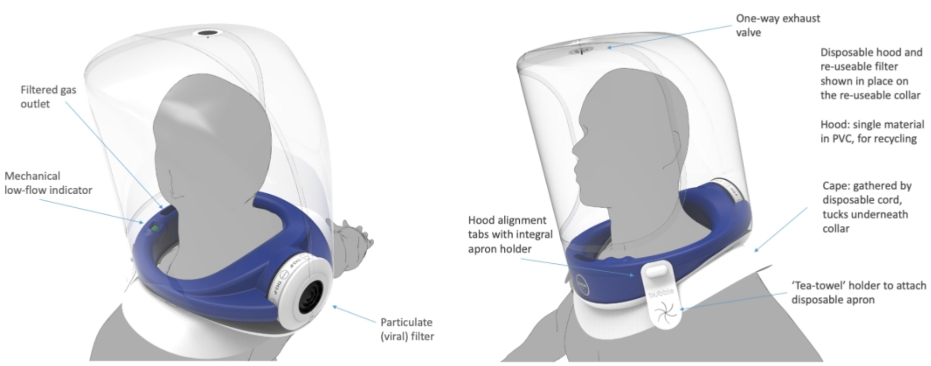


Bubble-PAPR comprises a medical-grade foam neck collar and a separate PVC hood. The universal fit collar draws air in through a filter via an impellor powered by an external battery. The collar has a mechanical low flow indicator and can be cleaned and reused by different users. The semi-rigid hood is pulled over the collar before donning and is secured by integrated straps.

762x283mm (72 x 72 DPI)

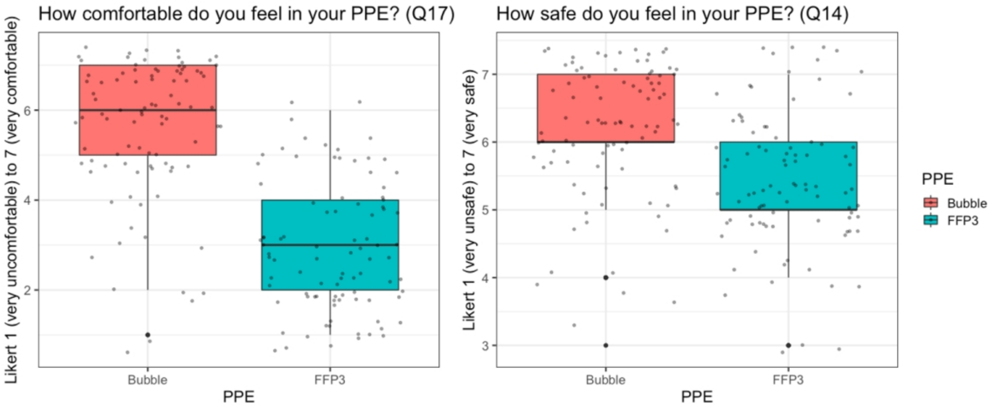


Reported comfort (primary) and safety (secondary) outcomes for Bubble-PAPR vs usual FFP3 face masks.

537x225mm (72 x 72 DPI)

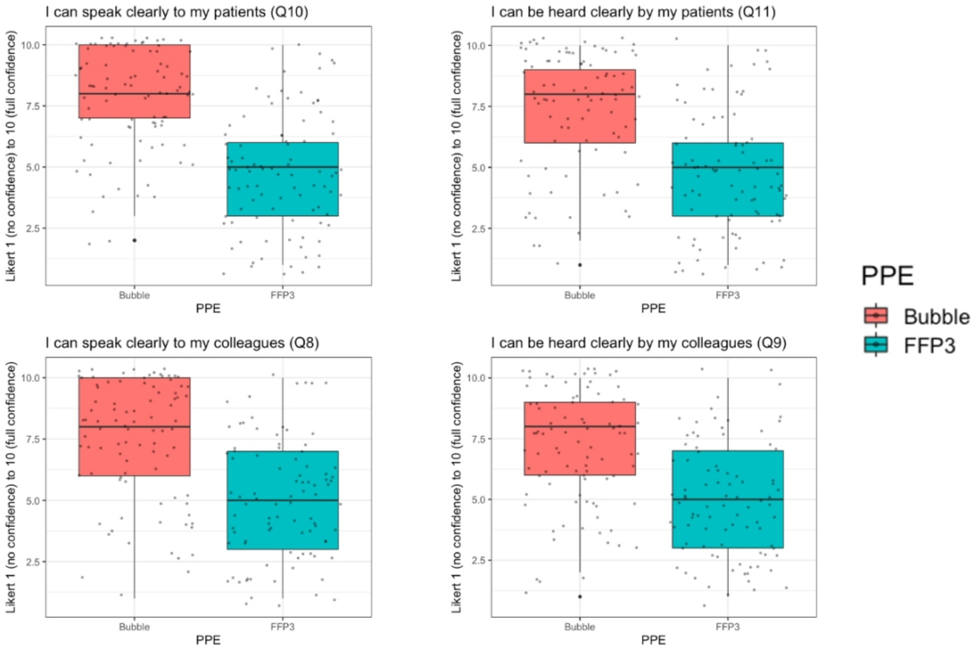


Secondary communication outcomes where a higher Likert scale response was considered better.

446x295mm (72 x 72 DPI)

**Table S1. (Supplemental)** Proposed framework for the safer adoption of a MacGyvered device. Adapted from Turner and colleagues.[7]

1. Define the problem and rule out the suitability of existing solutions
2. List benchmark safety indices for the device
3. Seek broader feedback from all stakeholders on the design's utility and potential pitfalls.
4. Perform laboratory-based and in situ simulations.
5. Introduce into low-risk clinical settings after local due process and patient consent.
6. Introduce into higher-risk clinical settings with a discrete group of trained 'super-users'.
7. Encourage an iterative cycle of feedback, review, re-design and improvement.
8. **Do not:** adopt, publish, endorse or disseminate via social media a MacGyvered device without data to support safety.


For peer review only - http://bmjopen.bmj.com/site/about/guidelines.xhtml

**Table S2. (Supplemental). Lab-based testing of the Bubble PAPR prior to clinical evaluation**

All of the bench tests detailed below were carried out by the Electrical and Biomedical Engineering (EMBE) team based at Wythenshawe Hospital (MFT), the University of Manchester Mechanical Aerospace and Civil Engineering team (UoM) or by INSPEC International, Salford, UK. A PAPR unit was supplied, and the Instructions for Use were followed by the independent tester, with a judgement made if they fulfilled particular requirements. Some requirements are supplemented by the qualitative or quantitative data collected in the questionnaires. Standards used were British Standard EN12941 (BS, 2008) and the European Regulations for Respiratory Protective Equipment EU2016/425 (ER, 2016).

| Relevant section of standard | Standard detail | Test location | Test detail | Results/notes | Pass/Fail |
|---|---|---|---|---|---|
| BS 6.1.1<br>ER 1.3.2 | Suitable resistance to wear and tear | MFT | PAPR units inspected after 1 week of continual use.<br>Images taken before and after. | Opinion. Baseline inspection +/- photograph. Review after 1 week | Pass 14/3/21 |
| | | | | | |
| BS 6.1.4<br>ER 1.2.1.2 | No sharp edges | MFT | Visual and physical inspection<br>Reports from staff evaluation | Opinion. Baseline inspection +/- photograph. Review after 1 week | Pass 14/3/21 |
| | | | | | |
| BS 6.3.2 | Fits a range of head sizes | MFT | Ten participants will undergo fit testing.<br>These participants will have height, weight and head circumference measured as part of this standard process. | Fit test data shared with EBME team. All fit factors >500 as per BS EN 12941 standard. | Pass 25/2/21 |
| | | | | | |
| BS 6.3.3.1 | Does not distort vision | MFT | The optical area appears transparent. | Inspection by EBME team | Pass 25/2/21 |
| BS 6.3.3.2 | Permits appropriate field of view | MFT | Reports from staff evaluation | Review of results from initial staff evaluations | Pass 14/3/21 |


For peer review only - http://bmjopen.bmj.com/site/about/guidelines.xhtml

| ER2.3 | | | | | |
|---|---|---|---|---|---|
| Relevant section of standard | Standard detail | Test location | Test detail | Results/notes | Pass/Fail |
| BS 6.9 | Cannot reverse airflow | MFT | Normal use.<br>Simulate blocked filter and blocked air duct.<br>Flowmeter. | | Pass 14/3/21 |
| BS 6.9 | Battery safe – protection from short circuit | MFT | EBME check on battery packs (suitable for purpose / recommended packs) | As per manufacturer documentation. Will not be separately tested. | Pass 25/2/21 |
| | | | | | |
| ER2.12 | Appropriate markings | MFT | Yoke manufactured with section for appropriate sticker | | Pass 25/2/21 |
| | | | | | |
| ER3.10.1 | Appropriate training provided | MFT | Instructions for use provided.<br>Training videos provided. | Instructions for Use provided to EBME.<br>Training videos available. | Pass 25/2/21 |
| | | | | | |
| No specific clause | Cleanable | MFT | Specification of foam material for yoke details cleaning methods, durability and material fatigue. | Specification provided to EBME. | Pass 25/2/21 |
| BS 6.16 | Mass shall not exceed 5kg. A maximum of 1.5kg shall be carried on the head. | MFT | Weigh the assembled Bubble PAPR | Assembled Bubble PAPR Weight = 1.4kg | Pass 14/3/21 |


For peer review only - http://bmjopen.bmj.com/site/about/guidelines.xhtml

| Relevant section of standard | Standard detail | Test location | Test detail | Results/notes | Pass/Fail |
|---|---|---|---|---|---|
| BS 6.1.3 | Repeated cleaning and disinfection – does not deteriorate | MFT | PAPR units are inspected after 1 week of continual use. Images taken before and after.<br><br>Specification of foam material for yoke details cleaning methods, durability and material fatigue. | Spec sheet and review after 1 week of use. | Pass 14/3/21 |
| | | | | | |
| BS 6.4 | Ingress protection test for 10 test subjects – using either or both methods (bitter and/or particulometer). | MFT | 10 users will undergo fit testing with particulometer. | Fit test data shared with EBME team. All fit factors >500 as per BS EN 12941 standard. | Pass 25/2/21 |
| ER 3.10.2 | Appropriate protection to eye and skin irritants | MFT | | | |



For peer review only

For peer review only - http://bmjopen.bmj.com/site/about/guidelines.xhtml

| Relevant section of standard | Standard detail | Test location | Test detail | Results/notes | Pass/Fail |
|---|---|---|---|---|---|
| BS 6.2 (6.4) | Repeated after hood/yoke is conditioned at maximum specified temperature and humidity.<br><br>Complete unit is stored for 72 +/- 1 hours at the upper extreme of temperature and humidity specified by the manufacturer. Unit is allowed to return to ambient conditions for 4 hours, then stored for 72 +/- 1 hours at the lower extreme of temperature and humidity. | MFT | Unit is subjected to particulometer fit testing after appropriate temperature conditioning. | *Conditioning beyond use on the ICU should not be required for the MFT in-house evaluation*<br><br>All fit tests took place on the Acute ICU at Wythenshawe | Pass 25/2/21 |
| | | | | | |
| BS 6.5 | Positive pressure inside the hood remains below 5mbar | MFT | 1 user and 1 dummy test head setup. Pressure measurement inside hood during regular use. | Measured pressure below 5mBar | Pass 14/3/21 |
| BS 6.6.2 | Exceeds manufacturer's minimum specified airflow for a period of at least 4 hours.<br><br>(The UK/EU regs do not specify a minimal flow. US regulations do, but this is not immediately relevant) | MFT, INSPEC | Test head and flow meter.<br><br>*Note the flow meter arrangement is slightly complex as the measurement itself can interfere with flow.* | Breathe Safety report reviewed. | Pass 25/2/21 |



For peer review only - http://bmjopen.bmj.com/site/about/guidelines.xhtml

| Relevant section of standard | Standard detail | Test location | Test detail | Results/notes | Pass/Fail |
|---|---|---|---|---|---|
| BS 6.7 | Check function of minimum airflow indicator. | MFT, INSPEC | Apply different flow rates to the yoke measured by external flow meter and evaluate performance of the minimum airflow indicator in units. | Breathe Safety report reviewed. | Pass 25/2/21 |
| | | | | | |
| EN 143<br>BS 6.8 | Clogging of filter | MFT, INSPEC | Flow through filter and yoke tested after 4 hours of use with an external flow meter. | Breathe Safety report reviewed. | Pass 25/2/21 |
| | | | | | |
| 6.13 | The carbon dioxide content of the inhalation air (dead space inside the hood) shall not exceed an average of 1% by volume.<br><br>There is a specific test-rig setup for this. A physiological surrogate model should provide adequate assurance that the $CO_2$ content inside the hood is <1% during normal use. | MFT, INSPEC | Oxygen and carbon dioxide (gas analysis) inside the hood measured as partial pressures and/or percentages using MFT EBME equipment. | Breathe Safety report reviewed. | Pass 25/2/21 |

For peer review only


For peer review only - http://bmjopen.bmj.com/site/about/guidelines.xhtml

| Relevant section of standard | Standard detail | Test location | Test detail | Results/notes | Pass/Fail |
|---|---|---|---|---|---|
| BS 6.15 | Exhalation means (valve) maximum flowrate and safe operation:<br><br>Hood performs adequately during normal use. Specifically; exhalation means:<br>• functions and can be replaced (new hood)<br>• functions in orientations encountered during normal use<br>• is protected against dirt and mechanical damage | MFT | Test subject wears hood.<br><br>Eventually, this section is supplemented with reports from staff evaluation. | Review of initial feedback from users in sim setting | Pass 14/3/21 |
| BS 6.15 | Exhalation means (valve) maximum flowrate and safe operation:<br><br>Continuous flow rate of 300 +/- 15 L/min is applied for a period of 60+/- 6 secs. | MFT | Flow generator (ventilator) and flow meter.<br><br>Visual inspection of exhalation valve. | Opinion of EBME team. The flow generator works as described with the test flowmeter supplied. Exhalation Valve inspection pass. | Pass 14/3/21 |


For peer review only - http://bmjopen.bmj.com/site/about/guidelines.xhtml

**Table S3. (Supplemental). Pilot data. Q14 & Q17 are Likert Scale items (rated 1-7) and Q8-11 are Visual Analogue Scale items (rated 0-100).**

| | Current PPE | | | | | | BUBBLE-PAPR | | | | | |
|---|---|---|---|---|---|---|---|---|---|---|---|---|
| | Q14 Safe to wear | Q17 Comfort to wear | Q8 Speak clearly to colleagues | Q9 Be heard by colleagues | Q10 Speak clearly to patients | Q11 Be heard by patients | Q14 Safe to wear | Q17 Comfort to wear | Q8 Speak clearly to colleagues | Q9 Be heard by colleagues | Q10 Speak clearly to patients | Q11 Be heard by patients |
| Participant | | | | | | | | | | | | |
| 1 | 3 | 3 | 10 | 20 | 0 | 0 | 4 | 5 | 75 | 75 | 80 | 80 |
| 2 | 4 | 2 | 15 | 20 | 10 | 30 | 7 | 5 | 85 | 80 | 60 | 70 |
| 3 | 4 | 4 | 20 | 20 | 30 | 30 | 5 | 6 | 90 | 90 | 90 | 90 |
| 4 | 5 | 2 | 25 | 25 | 10 | 10 | 6 | 6 | 95 | 95 | 95 | 85 |
| 5 | 5 | 4 | 33 | 40 | 25 | 25 | 6 | 5 | 75 | 70 | 85 | 90 |
| 6 | 6 | 3 | 30 | 25 | 30 | 30 | 6 | 5 | 65 | 70 | 55 | 66 |
| 7 | 7 | 2 | 20 | 30 | 25 | 25 | 7 | 5 | 70 | 70 | 65 | 65 |
| 8 | 4 | 3 | 45 | 50 | 20 | 30 | 7 | 6 | 90 | 95 | 90 | 90 |
| Mean | 4.8 | 2.9 | 24.8 | 28.8 | 18.8 | 22.5 | 6.0 | 5.4 | 80.6 | 80.6 | 77.5 | 79.5 |
| SD | 1.3 | 0.8 | 11.1 | 10.9 | 10.9 | 11.3 | 1.1 | 0.5 | 10.8 | 11.2 | 15.4 | 11.0 |



For peer review only - http://bmjopen.bmj.com/site/about/guidelines.xhtml

**Table S4. (Supplemental) Fit testing data from the first 10 participants.**

Test protocol HSE INDG 479. Pass level set at a fit factor of 500.

| Subject | Self-reported height | Self-reported weight | BMI | Normal Breathing 1 | Deep Breathing | Head Side to Side | Head Up and Down | Talking | Bending at the waist | Normal Breathing 2 | Overall Fit Factor |
|---|---|---|---|---|---|---|---|---|---|---|---|
| **1** | 1.86 | 74 | 21.4 | 79705 | 43647 | 125478 | 11125 | 107899 | 1339 | 76152 | **7757** |
| **2** | 1.95 | 75 | 19.7 | 53792 | 52343 | 59440 | 51673 | 52733 | 50433 | 45961 | **52075** |
| **3** | NR | NR | NR | 38867 | 36699 | 37097 | 41474 | 39500 | 36884 | 37465 | **38217** |
| **4** | 1.82 | 65 | 19.6 | 17745 | 6622 | 3149 | 5028 | 31996 | 30520 | 31326 | **8539** |
| **5** | 1.65 | 55 | 20.2 | 24945 | 25215 | 3885 | 8097 | 28877 | 29107 | 24393 | **12268** |
| **6** | 1.67 | 58 | 20.8 | 24617 | 25608 | 25581 | 25225 | 20107 | 1924 | 23517 | **9088** |
| **7** | 1.52 | 47 | 20.3 | 28747 | 30700 | 33203 | 15275 | 31671 | 8327 | 26041 | **19829** |
| **8** | 1.65 | 65 | 23.9 | 27282 | 31318 | 34900 | 9697 | 29093 | 3514 | 25770 | **12544** |
| **9** | 1.83 | 115 | 34.3 | 11182 | 1123 | 11028 | 24524 | 24692 | 2537 | 23704 | **4408** |
| **10** | 1.59 | 75 | 29.7 | 25760 | 3419 | 16125 | 2433 | 25523 | 1552 | 26154 | **4588** |
| | | | | | | | | | | | |
| **Mean values** | | | | **33264** | **25669** | **34989** | **19455** | **39209** | **16614** | **34048** | **16931** |



For peer review only - http://bmjopen.bmj.com/site/about/guidelines.xhtml

**Table S5.** Rating scales, summary results and comparisons across all of the questionnaire domains.

| | PPE | Q1 Confidence in donning | Q2 Confidence in donning without dislodging other PPE | Q3 Wear with glasses/goggles | Q4 Protect yourself from respiratory infection | Q5 Protect patient from infection from you | Q6 Safely care for your patient | Q7 Safely roll patient | Q8 Speak to staff | Q9 Be heard by staff | Q10 Speak to patient | Q11 Be heard by patient | Q12 Doff safely | Q13 Doff without dislodging glasses |
|---|---|---|---|---|---|---|---|---|---|---|---|---|---|---|
| Rating scale | From: | 0 - no confidence | 0 - no confidence | 0 - no confidence | 0 - no confidence | 0 - no confidence | 0 - no confidence | 0 - no confidence | 0 - no confidence | 0 - no confidence | 0 - no confidence | 0 - no confidence | 0 - no confidence | 0 - no confidence |
| | To: | 10 - fully confident | 10 - fully confident | 10 - fully confident | 10 - fully confident | 10 - fully confident | 10 - fully confident | 10 - fully confident | 10 - fully confident | 10 - fully confident | 10 - fully confident | 10 - fully confident | 10 - fully confident | 10 - fully confident |
| | | | | | | | | | | | | | | |
| RPE type | FFP3 | 8.9 (1.4) [3 - 10] | 8.3 (2) [2 - 10] | 6.9 (2.6) [2 - 10] | 8.2 (1.6) [4 - 10] | 8.2 (1.7) [2 - 10] | 8.4 (1.4) [5 - 10] | 8.2 (1.8) [2 - 10] | 5.1 (2.4) [1 - 10] | 4.9 (2.3) [1 - 10] | 4.8 (2.4) [1 - 10] | 4.7 (2.5) [1 - 10] | 8.1 (1.9) [2 - 10] | 6.2 (2.5) [1 - 10] |
| | Bubble | 7.4 (1.8) [3 - 10] | 7.7 (1.8) [2 - 10] | 7.6 (1.9) [3 - 10] | 8.6 (1.6) [3 - 10] | 8.5 (1.8) [2 - 10] | 8.0 (2) [2 - 10] | 7.8 (2.2) [2 - 10] | 7.5 (2.4) [1 - 10] | 7.1 (2.3) [1 - 10] | 7.8 (2.1) [2 - 10] | 7.4 (2.4) [1 - 10] | 8.0 (1.8) [2 - 10] | 7.8 (1.9) [2 - 10] |
| Comparison | Mean difference | -1.48 | -0.55 | 0.7 | 0.43 | 0.3 | -0.42 | -0.42 | 2.38 | 2.16 | 2.99 | 2.7 | -0.1 | 1.66 |
| | 95% CI | -1.9 to -0.99 | -1.12 to 0.02 | -0.00 to 1.40 | -0.04 to 0.89 | -0.18 to 0.78 | -0.91 to 0.07 | -0.98 to 0.15 | 1.66 to 3.11 | 1.45 to 2.88 | 2.36 to 3.62 | 1.97 to 3.43 | -0.63 to 0.43 | 0.98 to 2.34 |
| | | Favours FFP3 | No difference | Favours Bubble | No difference | No difference | No difference | No difference | Favours Bubble | Favours Bubble | Favours Bubble | Favours Bubble | No difference | Favours Bubble |
| | Adjusted p | <0.001 | 0.058 | 0.049 | 0.070 | 0.217 | 0.092 | 0.144 | <0.001 | <0.001 | <0.001 | <0.001 | 0.711 | <0.001 |

| | PPE | Q14 How safe does it feel | Q15 Worried about own health | Q16 Worried others health | Q17 Comfortable | Q18 Don ease | Q19 Doff ease | Q20a Restricted communication | Q20b Vision distorted with Bubble | Q20c Read monitors, computers, and notes with Bubble | Q21 Pressure marks on head/face | Q22 Pain on head/face | Q23 Leave clinical area early due to RPE |
|---|---|---|---|---|---|---|---|---|---|---|---|---|---|
| Rating scale | From: | 1 - very unsafe | 1 - not worried at all | 1 - not worried at all | 1 - very uncomfortable | 1 - not at all easy | 1 - not at all easy | 1 - not at all restricted | 1 - not at all affected | 1 - clear at all times | 1 - never | 1 - never | 1 - never |
| | To: | 7 very safe | 7 - very worried | 7 - very worried | 7 - very comfortable | 7 - very easy | 7 - very easy | 7 - very restricted | 7 - very affected | 7 - not clear at all | 7 - always | 7 - always | 7 - always |
| | | | | | | | | | | | | | |
| RPE type | FFP3 | 5.4 (1.0) [3 - 7] | 3.2 (1.5) [1 - 7] | 3.2 (1.5) [1 - 7] | 3 (1.4) [1 - 6] | 4.9 (1.3) [2 - 7] | 5.1 (1.3) [2 - 7] | 5.4 (1.4) [1 - 7] | - | - | 5.8 (1.4) [1 - 7] | 5.3 (1.4) [1 - 7] | 3.6 (1.6) [1 - 7] |
| | Bubble | 6.2 (0.9) [3 - 7] | 2.3 (1.6) [1 - 7] | 2.3 (1.7) [1 - 7] | 5.6 (1.6) [1 - 7] | 5.5 (1.4) [2 - 7] | 5.7 (1.2) [2 - 7] | 3.9 (1.7) [1 - 7] | 3.2 (1.9) [1 - 7] | 5.6 (1.5) [2 - 7] | 1.3 (0.8) [1 - 6] | 1.4 (0.9) [1 - 6] | 1.5 (1) [1 - 6] |
| Comparison | Mean difference | 0.73 | -0.92 | -0.93 | 2.68 | 0.62 | 0.63 | -1.49 | - | - | -4.54 | -3.99 | -2.13 |
| | 95% CI | 0.45 to 0.99 | -1.36 to -0.49 | -1.36 to -0.48 | 2.23 to 3.14 | 0.21 to 1.02 | 0.26 to 0.99 | -1.95 to -1.04 | - | - | -4.90 to -4.17 | -4.35 to -3.63 | -2.51 to - 1.75 |
| | | Favours Bubble | Favours Bubble | Favours Bubble | Favours Bubble | Favours Bubble | Favours Bubble | Favours Bubble | No comparator | No comparator | Favours Bubble | Favours Bubble | Favours Bubble |
| | Adjusted p | <0.001 | <0.001 | <0.001 | <0.001 | 0.003 | 0.002 | <0.001 | - | - | <0.001 | <0.001 | <0.001 |

For peer review only

Figure S1. (Supplemental) Participant job roles.

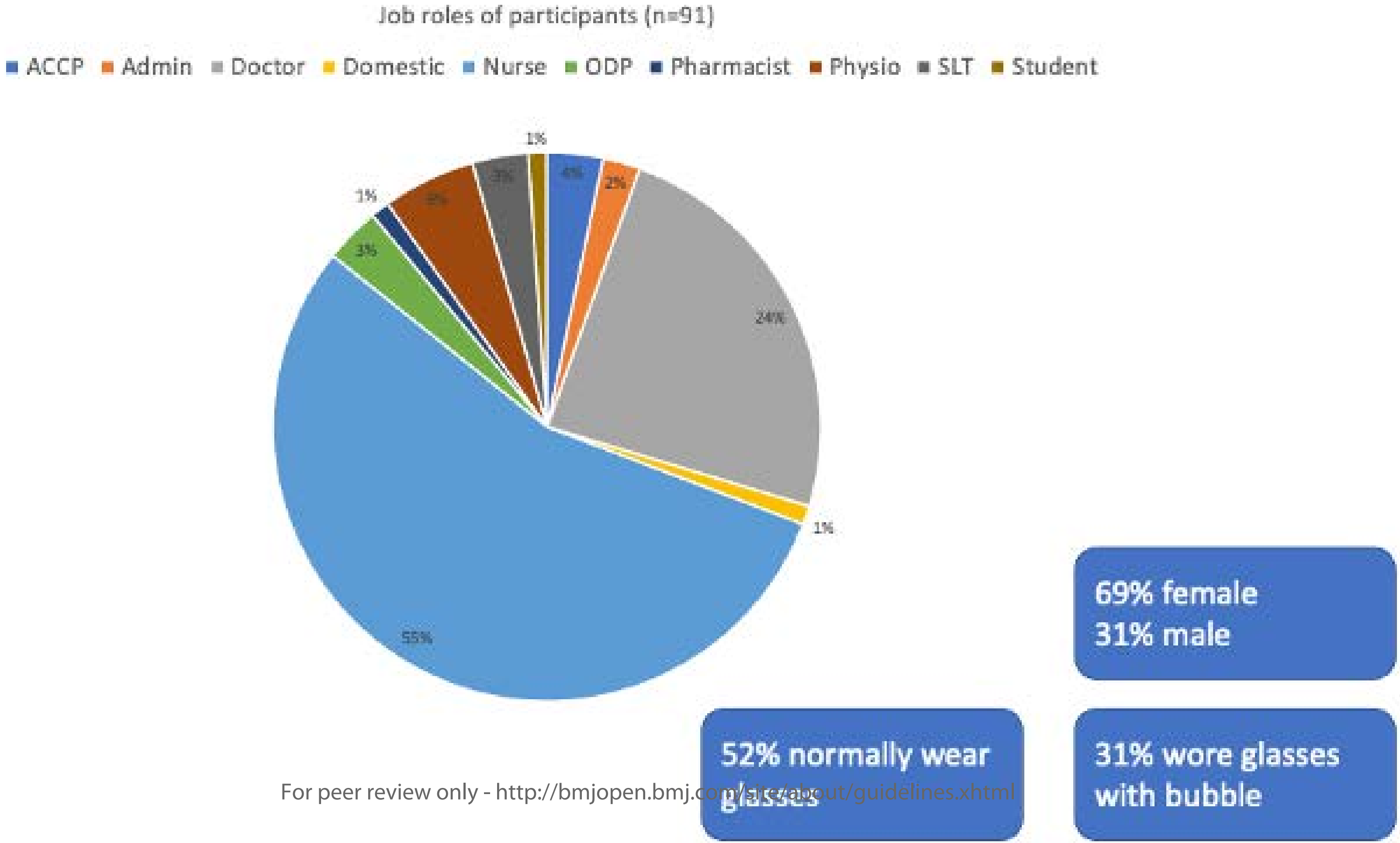

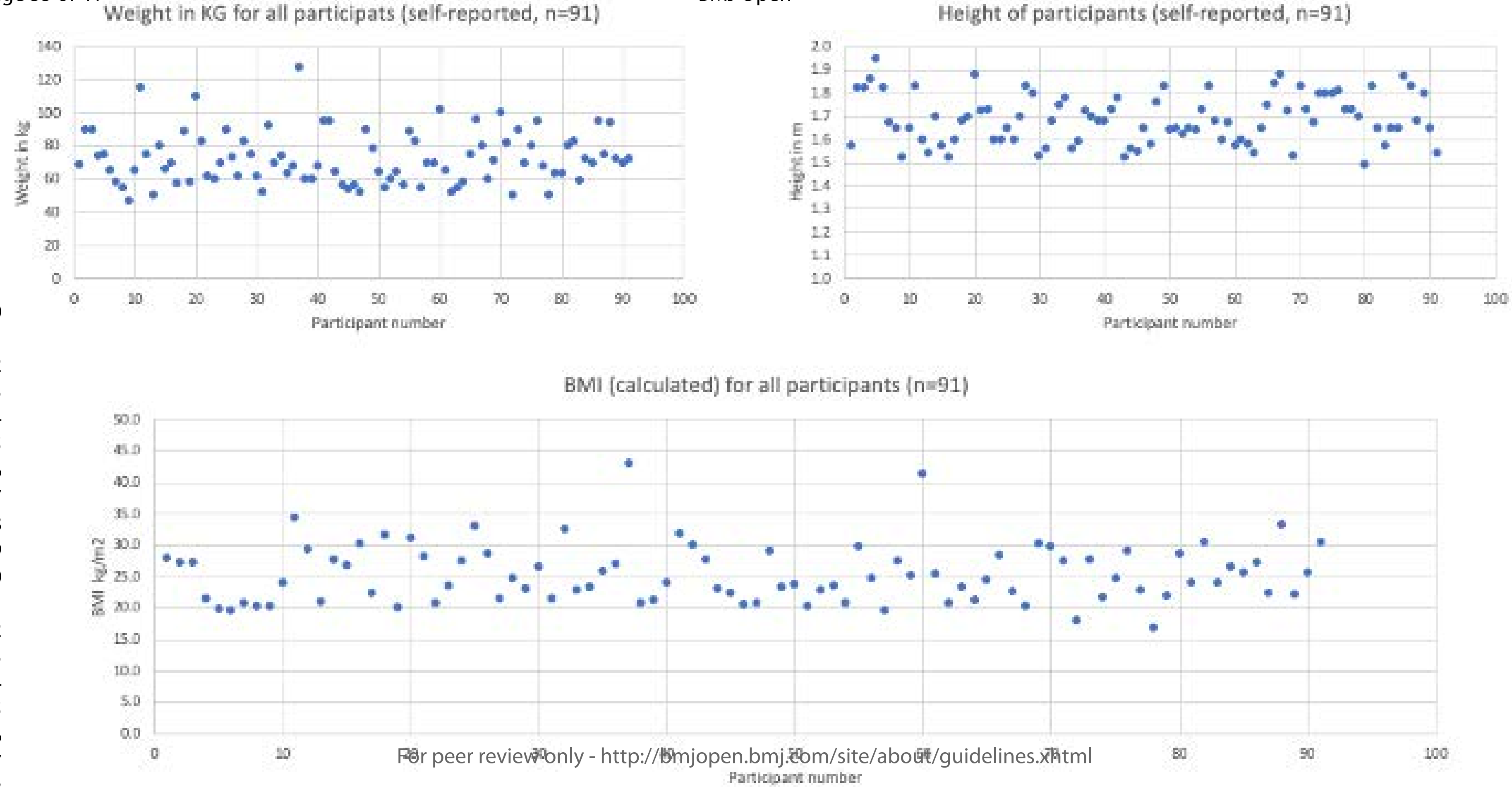


For peer review only - http://bmjopen.bmj.com/site/about/guidelines.xhtml

Figure S2. (Supplemental) Self-reported weight, height and BMI of staff participants.

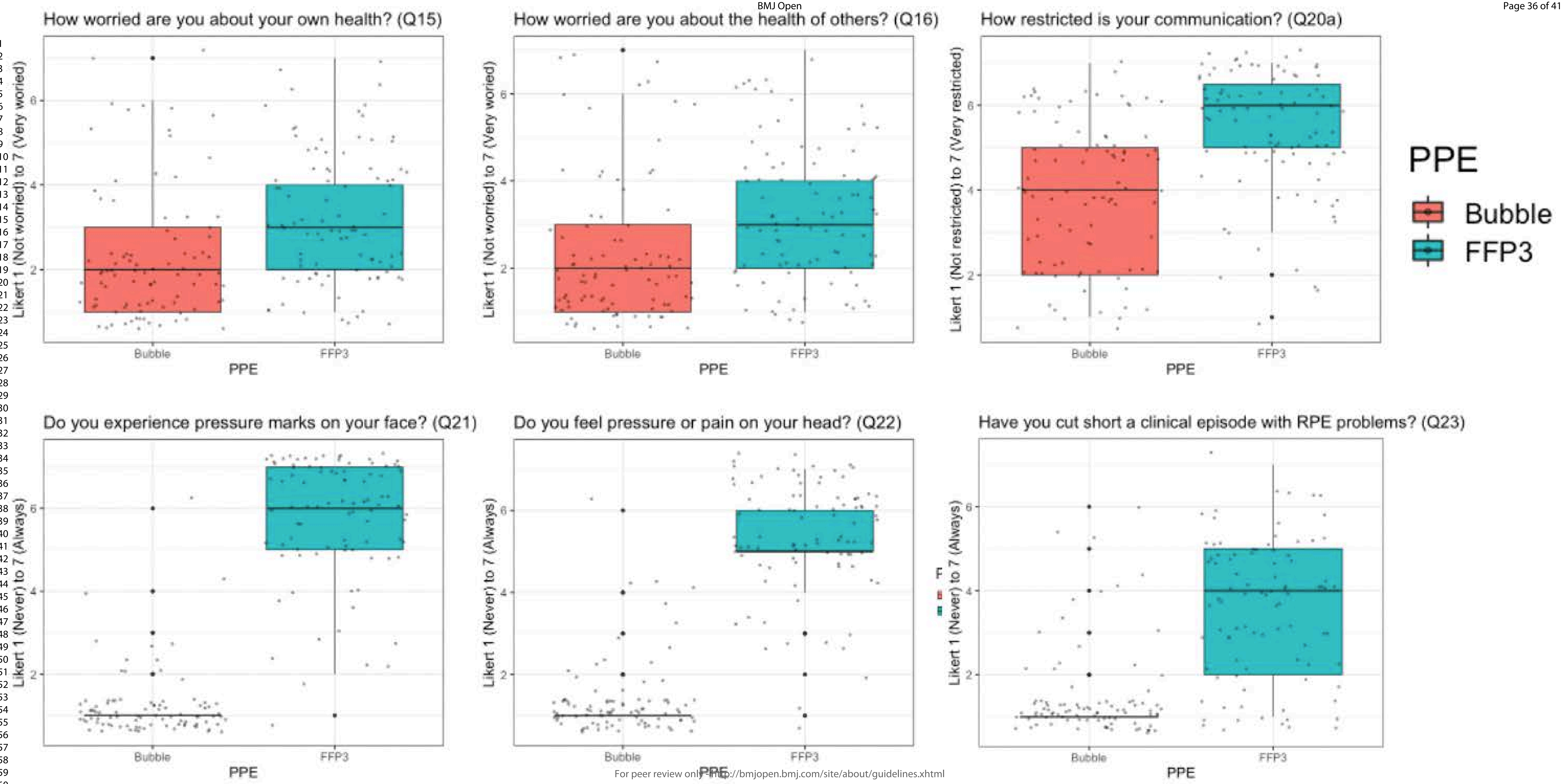


For peer review only - http://bmjopen.bmj.com/site/about/guidelines.xhtml

Figure S3. (Supplemental) Secondary outcomes where a lower Likert scale response was considered better.

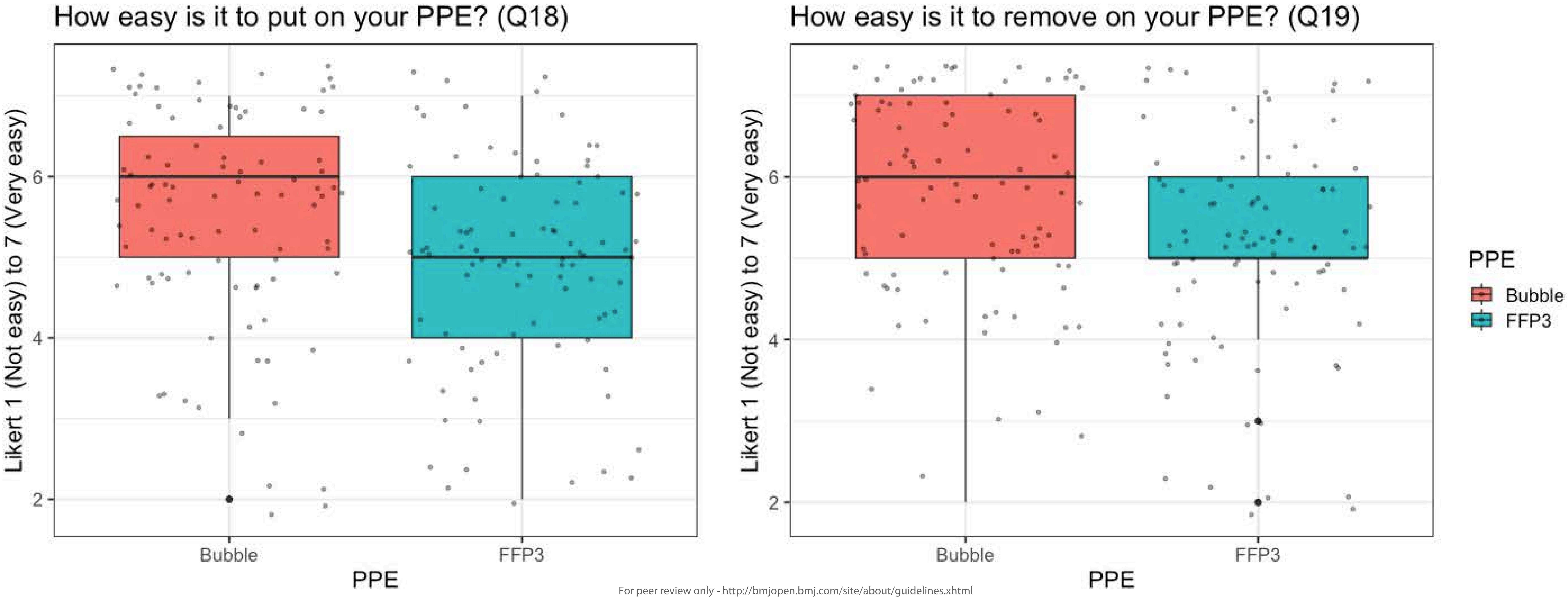




Figure S4. (Supplemental) Ease of donning and doffing of Respiratory Protective Equipment.

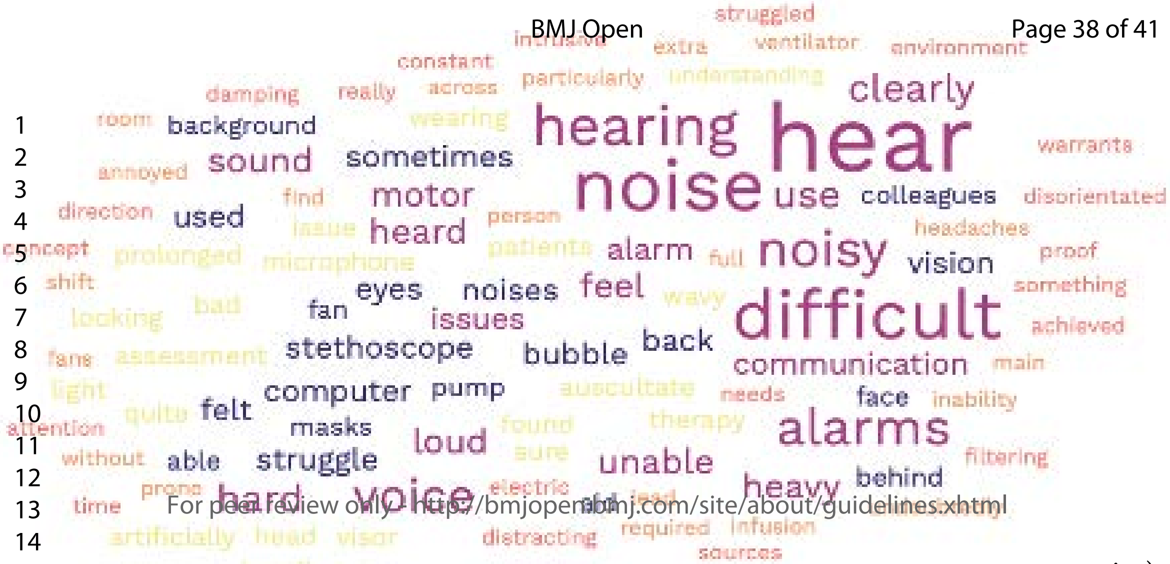


For peer review only - http://bmjopen.bmj.com/site/about/guidelines.xhtml

Figure S5. (Supplemental). Word clouds from the free text feedback. Negative comments (all categories).

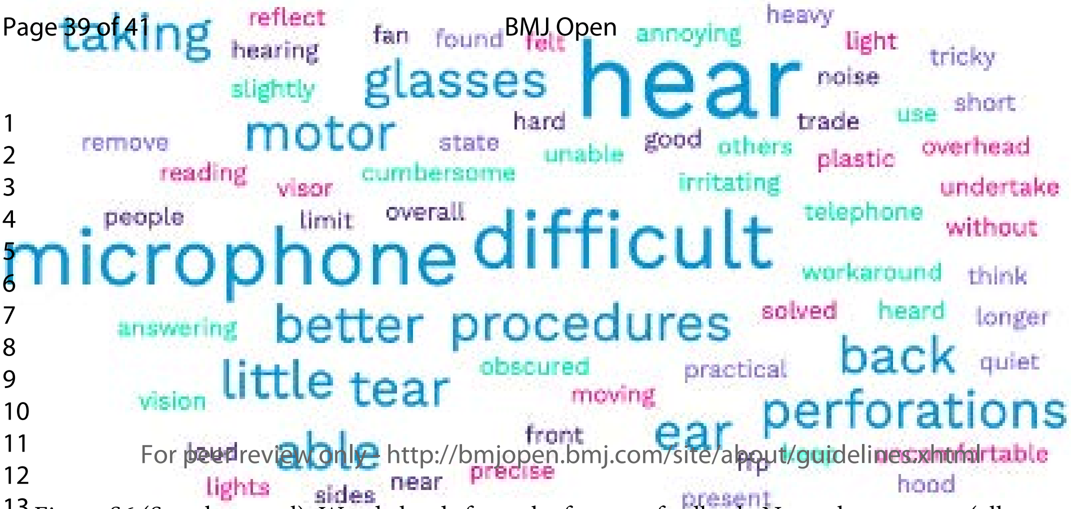




Figure S6 (Supplemental). Word clouds from the free text feedback. Neutral comments (all categories).

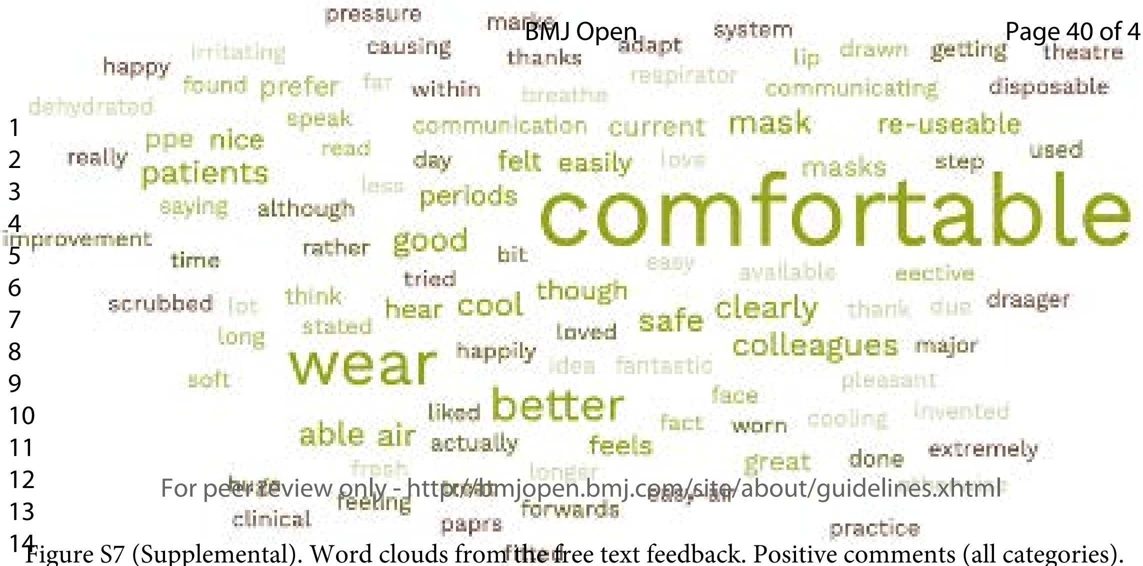




Figure S7 (Supplemental). Word clouds from the free text feedback. Positive comments (all categories).

STROBE Statement—checklist of items that should be included in reports of observational studies

| | Item No | Recommendation | Page No |
|---|---|---|---|
| **Title and abstract** | 1 | (*a*) Indicate the study's design with a commonly used term in the title or the abstract | 1 |
| | | (*b*) Provide in the abstract an informative and balanced summary of what was done and what was found | 2 |
| **Introduction** | | | |
| Background/rationale | 2 | Explain the scientific background and rationale for the investigation being reported | 3 |
| Objectives | 3 | State specific objectives, including any prespecified hypotheses | 4 |
| **Methods** | | | |
| Study design | 4 | Present key elements of study design early in the paper | 5 |
| Setting | 5 | Describe the setting, locations, and relevant dates, including periods of recruitment, exposure, follow-up, and data collection | 5 |
| Participants | 6 | (*a*) *Cohort study*—Give the eligibility criteria, and the sources and methods of selection of participants. Describe methods of follow-up<br>*Case-control study*—Give the eligibility criteria, and the sources and methods of case ascertainment and control selection. Give the rationale for the choice of cases and controls<br>*Cross-sectional study*—Give the eligibility criteria, and the sources and methods of selection of participants | 6 |
| | | (*b*) *Cohort study*—For matched studies, give matching criteria and number of exposed and unexposed<br>*Case-control study*—For matched studies, give matching criteria and the number of controls per case | |
| Variables | 7 | Clearly define all outcomes, exposures, predictors, potential confounders, and effect modifiers. Give diagnostic criteria, if applicable | 6 and Supplemental Material |
| Data sources/ measurement | 8* | For each variable of interest, give sources of data and details of methods of assessment (measurement). Describe comparability of assessment methods if there is more than one group | 6 |
| Bias | 9 | Describe any efforts to address potential sources of bias | 5 |
| Study size | 10 | Explain how the study size was arrived at | 6 |
| Quantitative variables | 11 | Explain how quantitative variables were handled in the analyses. If applicable, describe which groupings were chosen and why | 6 |
| Statistical methods | 12 | (*a*) Describe all statistical methods, including those used to control for confounding | 6 |
| | | (*b*) Describe any methods used to examine subgroups and interactions | N/A |


For peer review only - http://bmjopen.bmj.com/site/about/guidelines.xhtml

| | | | |
|---|---|---|---|
| | | (*c*) Explain how missing data were addressed | Intention to treat. No missing data. |
| | | (*d*) *Cohort study*—If applicable, explain how loss to follow-up was addressed<br>*Case-control study*—If applicable, explain how matching of cases and controls was addressed<br>*Cross-sectional study*—If applicable, describe analytical methods taking account of sampling strategy | |
| | | (*e*) Describe any sensitivity analyses | N/A |

| **Results** | | | |
|---|---|---|---|
| Participants | 13* | (a) Report numbers of individuals at each stage of study—eg numbers potentially eligible, examined for eligibility, confirmed eligible, included in the study, completing follow-up, and analysed | 7 |
| | | (b) Give reasons for non-participation at each stage | 7 |
| | | (c) Consider use of a flow diagram | Not required |
| Descriptive data | 14* | (a) Give characteristics of study participants (eg demographic, clinical, social) and information on exposures and potential confounders | 7 |
| | | (b) Indicate number of participants with missing data for each variable of interest | N/A |
| | | (c) *Cohort study*—Summarise follow-up time (eg, average and total amount) | 7 |
| Outcome data | 15* | *Cohort study*—Report numbers of outcome events or summary measures over time | *7* |
| | | *Case-control study*—Report numbers in each exposure category, or summary measures of exposure | |
| | | *Cross-sectional study*—Report numbers of outcome events or summary measures | |
| Main results | 16 | (*a*) Give unadjusted estimates and, if applicable, confounder-adjusted estimates and their precision (eg, 95% confidence interval). Make clear which confounders were adjusted for and why they were included | 7 |
| | | (*b*) Report category boundaries when continuous variables were categorized | 7 |
| | | (*c*) If relevant, consider translating estimates of relative risk into absolute risk for a meaningful time period | 7 |
| Other analyses | 17 | Report other analyses done—eg analyses of subgroups and interactions, and sensitivity analyses | 8 |
| **Discussion** | | | |
| Key results | 18 | Summarise key results with reference to study objectives | 9 |
| Limitations | 19 | Discuss limitations of the study, taking into account sources of potential bias or imprecision. Discuss both direction and magnitude of any potential bias | 9 |
| Interpretation | 20 | Give a cautious overall interpretation of results considering objectives, limitations, multiplicity of analyses, results from similar studies, and other relevant evidence | 9 |
| Generalisability | 21 | Discuss the generalisability (external validity) of the study results | 10,11 |
| **Other information** | | | |
| Funding | 22 | Give the source of funding and the role of the funders for the present study and, if applicable, for the original study on which the present article is based | 12 |


For peer review only - http://bmjopen.bmj.com/site/about/guidelines.xhtml
